# Structure–Activity Relationship and Crystallographic Study of New Monobactams

**DOI:** 10.1021/acs.jmedchem.5c02427

**Published:** 2026-02-03

**Authors:** Vid Kavaš, Carlos Contreras-Martel, Stane Pajk, Damijan Knez, Alexandre Martins, Thomas A. Gould, David I. Roper, Irena Zdovc, Andréa Dessen, Martina Hrast Rambaher, Stanislav Gobec

**Affiliations:** † Department of Pharmaceutical Chemistry, Faculty of Pharmacy, 37663 University of Ljubljana, Aškerčeva cesta 7, 1000 Ljubljana, Slovenia; ‡ 55543 University Grenoble Alpes, CNRS, CEA, Institut de Biologie Structurale (IBS), 38044 Grenoble, France; § School of Life Sciences, 2707 University of Warwick, Gibbet Hill Road, Coventry CV4 7AL, U.K.; ∥ Institute of Microbiology and Parasitology, Veterinary Faculty, University of Ljubljana, Gerbičeva 60, 1000 Ljubljana, Slovenia

## Abstract

Monobactams, a subclass of β-lactam antibiotics with a monocyclic scaffold, are uniquely resistant to hydrolysis by metallo-β-lactamases, providing a distinct therapeutic advantage. Here, we report an *in silico*-based structure–activity relationship (SAR) investigation of aztreonam-related monobactams. A focused library of monobactam derivatives was synthesized and evaluated for inhibition of penicillin-binding proteins (PBPs) and antibacterial activity. Ten compounds, including aztreonam, were crystallized with truncated PBP1b from *Streptococcus pneumoniae*, used as a model PBP. Potent PBP1b inhibitors were developed, although high enzymatic potency was not always reflected in strong antibacterial activity. Certain derivatives showed activity against *Staphylococcus aureus*, which is typically resistant to monobactams. 2D similarity search identified potent inhibitors active against *Escherichia coli*, *Klebsiella pneumoniae*, and *Acinetobacter baumannii*. Crystal structures revealed previously unrecognized binding interactions, including a halogen bond with a conserved threonine residue, underscoring the potential of these interactions to support the development of more potent PBP inhibitors.

## Graphical abstract

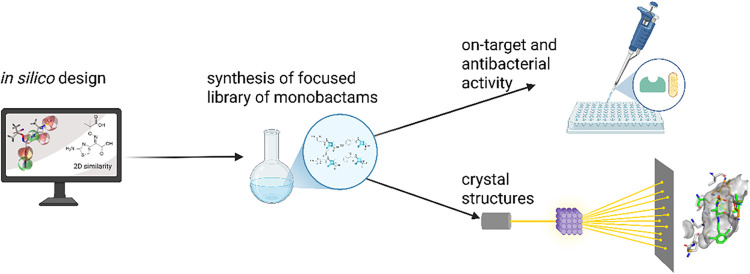

## 1 Introduction

Antimicrobial resistance (AMR) is one of the biggest global public health challenges. Infections are on the rise and are becoming increasingly difficult to treat with current therapies.^1,2^ Drug-resistant bacteria are associated with approximately 4.7 million deaths per year, a number that is expected to rise to 8.2 million by 2050.^3^ To successfully combat resistant bacteria, investment in research and development of new antibacterial agents, particularly those with novel chemical structures or mechanisms of action, is needed. Despite ongoing efforts, the global antibiotic pipeline faces challenges due to limited innovation and worldwide availability of both new and existing treatments.^4–6^

Bacterial peptidoglycan has a dual function: it provides structural integrity and enables bacteria to withstand intracellular pressure. At the same time, it helps maintain a well-defined cell shape that is inherited across generations.^7,8^ Peptidoglycan undergoes a complex biosynthesis involving approximately 30 enzymatic reactions.^9^ Among these components, penicillin-binding proteins (PBPs) play a crucial role in later stages as they catalyze the cross-linking of glycan chains by transpeptidation. The active site interacts with the d-Ala-d-Ala fragment of the peptidoglycan structure, which is then attacked by the amino group of a neighboring muropeptide chain. As a result, cross-linking generally occurs between the fourth amino acid of one peptide stem and the third amino acid of another (4 → 3 cross-linking).^7,8^ PBPs are validated targets for antibacterial drug discovery as they are inhibited by β-lactam antibiotics.^10,11^

The transpeptidase domain of PBPs consists of an α-β subdomain and an all-α subdomain, with the active site located between them. The transpeptidase activity depends on specific residues within three conserved motifs, SxxK, SxN and KTGT, which are mainly located in the active site. In particular, the serine residue in the SxxK motif plays a key role in the two-step cross-linking reaction.^14^

β-Lactam antibiotics are a diverse family of bactericidal drugs classified into several subgroups, including penicillins, cephalosporins, carbapenems and monobactams, which inhibit PBPs by targeting the transpeptidase domain. The strained β-lactam ring attacks the serine residue within the transpeptidase domain, resulting in the acylation by the antibiotic, as shown in Scheme 1. These stable covalent complexes effectively block the enzyme and prevent it from catalyzing peptidoglycan cross-linking. Structural and physicochemical differences between the subgroups influence their spectrum of activity; compounds with higher lipophilicity tend to be more effective against Gram-positive bacteria, while hydrophilic ones have greater activity against Gram-negative bacteria.^9,15,16^

**Scheme 1:**
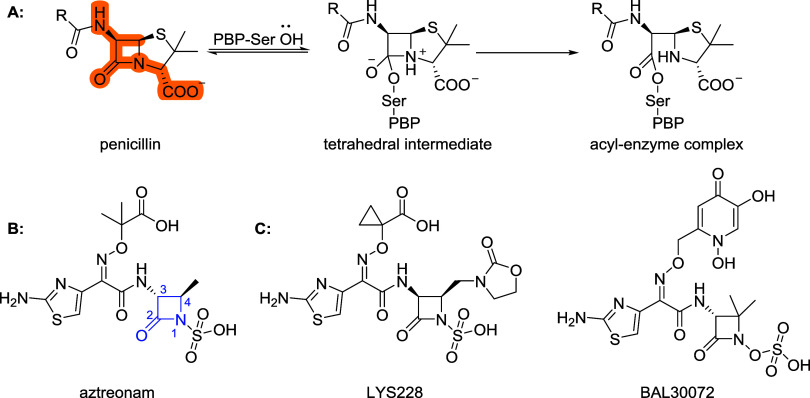
(A) Mode of Action of β-Lactam Antibiotics, the Highlighted Structure Shows the Part That Resembles the d-Ala-d-Ala Fragment. (B) Aztreonam, the Only Registered Monobactam, with the Numbering of β-Lactam Ring, (C) Monocyclic β-Lactams in Clinical Trials^12,13^

Aztreonam, the only approved monobactam, has a monocyclic β-lactam structure with an *N*1-sulfonate, as shown in Scheme 1. It selectively targets aerobic enteric bacteria and *Pseudomonas aeruginosa*, while it has negligible activity against anaerobes and Gram-positive strains such as *Staphylococcus aureus*, *Streptococcus pneumoniae* and *Enterococcus faecalis*. Aztreonam binds potently to PBP3 of Gram-negative bacteria, with lower affinity for PBP1a. It is intrinsically stable against common β-lactamases (e.g., TEM, OXA-2 and SHV-1), but its efficacy against multidrug-resistant, β-lactamase-producing organisms has declined due to the emergence of extended-spectrum β-lactamases (ESBLs) and serine carbapenemases. However, the monobactam core resists hydrolysis by metallo-β-lactamases (MBLs), which is a unique advantage over other β-lactam antibiotics.^17,18^ Following the discovery of aztreonam, researchers have synthesized several monobactams by introducing various substitutions at the 3- or 4-position of the β-lactam ring or by modifying the oxime side chain. These structural modifications aim to broaden antibacterial activity and to improve resistance against β-lactamases. The best-known candidates include carumonam (developed by Takeda), BAL30072 (developed by Basilea Pharmaceutica), LYS228 (developed by Novartis) and AIC499 (developed by AiCuris). Of these, LYS228 has reached Phase II clinical trials (NCT03377426) for the treatment of complicated urinary tract infections. The promising effect of LYS228 emphasizes the importance of ongoing research in this area and strengthens the potential for the discovery of a new antibacterial agent that is effective against drug-resistant infections.^12,13,18–20^

Recently, the design of monobactams bearing simpler C3 side chains (e.g., benzyl) replacing the bulky aztreonam substituent has been proposed to broaden antibacterial activity. The bulky C3 moiety of aztreonam reduces PBP1b inhibition due to the lack of corresponding interactions in PBP1b compared to PBP3.^21^ Targeting PBP1b was selected in preference to PBP3, as inhibition of PBP1b is less likely to trigger the bacterial SOS response, which can enable bacterial survival and evasion of β-lactam–mediated killing.^21^ Although aztreonam is a relatively weak PBP1b inhibitor, its crystal structure in complex with the enzyme provides a valuable foundation for structure-based design of improved inhibitors. To test this hypothesis and to obtain structural information needed for structure-based drug design, we designed and synthesized a focused library of monobactams. The target compounds were evaluated for their inhibition of *S. pneumoniae* PBP1b, and 10 crystal structures were solved at high resolution.

## 2 Results and Discussion

### 2.1 In Silico Design

Our design strategy was based on the use of the commercially available (2*S*,3*S*)-3-amino-2-methyl-4-oxoazetidine-1-sulfonic acid (**1**) as a synthetic starting point. Our goal was to identify new side chains using two *in silico* strategies: pharmacophore modeling and 2D similarity search.

First, we generated three different structure-based pharmacophore models based on a crystal structure of aztreonam covalently bound to PBP1b of *Escherichia coli* (PDB ID: 5HLB).^21^ We created a monobactam database by forming an amide bond between the 3-amino group of the monobactam core and in-house library of carboxylic acids using MarvinSketch (Marvin 23.12.0, 2023, ChemAxon (http://www.chemaxon.com)) and the KNIME Analytics platform.^22^ We then performed a virtual screening of the three pharmacophore models using LigandScout 4.4.9 (Inte:Ligand GmbH)^23^ and identified 26 compounds (Table S1) that were hits in all models. We synthesized 6 compounds (**2–7**) based on combined score and chemical diversity (Scheme 2, **A**). With this approach, we aimed to find structurally diverse and potent inhibitors of the PBP transpeptidase domain. In the next iteration, we built on the most potent hit **6** of this series, a derivative of phenylacetic acid, which is also present in penicillins such as benzylpenicillin, ampicillin, and amoxicillin.^18^ Therefore, we decided to create a series of monobactam analogues **8–21** and investigate their structure–activity relationship by introducing different 2-arylacetic acids (Scheme 2, **B**).

**Scheme 2:**
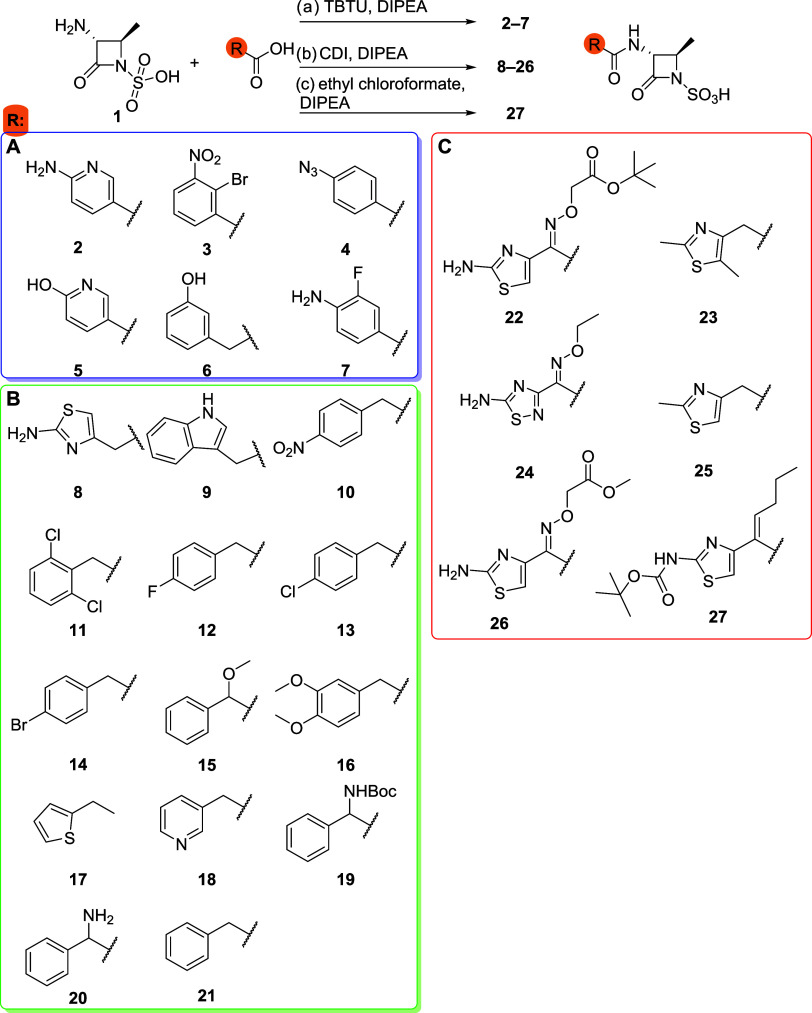
Commercially Available Monobactam 1 and Syntheses of Amide Derivatives: (a) Carboxylic Acid, TBTU, DIPEA, 50 °C, 15 min, Then Core 1, 50 °C, 24 h, (b) Carboxylic Acid, CDI, 1 h, rt, Then 1 and DIPEA, 50 °C, 16 h, (c) Ethyl Chloroformate, Carboxylic Acid, DIPEA, 0 °C, 1 h, Then DIPEA and 1, rt, 24 h^a^

a (A) Compounds based on the pharmacophore, (B) arylacetic acid analogues based on compound 6, (C) compounds based on 2D similarity.

In parallel, we performed a 2D similarity search of the carboxylic acid (*Z*)-2-((((2-aminothiazol-4-yl)­(carboxy)­methylene)­amino)­oxy)-2-methylpropanoic acid (ATMO), which is present in aztreonam, using commercially available carboxylic acids from three vendors (BLDPharm, ApolloScientific, AstaTech). We selected the hits with the highest similarity that had not yet been reported, resulting in compounds **22–27** (Scheme 2C).

### 2.2 Chemistry

We synthesized 26 monobactams using three different coupling reagents, modifying the 3-amino group of the monobactam core (**1**) to form amides. For the first series of compounds, we used various carboxylic acids and 2-(1*H*-benzotriazol-1-yl)-1,1,3,3-tetramethyluronium tetrafluoroborate (TBTU), followed by the addition of the monobactam core and *N*,*N*-diisopropylethylamine (DIPEA), as described in Method a (Scheme 2). Compounds with an arylacetic acid side chain (**8–21**) were synthesized using carbonyldiimidazole (CDI) as the activating agent (Method b, Scheme 2), which proved to be most effective for this type of acids. Compounds **22–27** were partially synthesized using method b, but also using ethyl chloroformate (Method c, Scheme 2) as an alternative approach. Purification was performed in two runs by reversed-phase flash chromatography: first with a 50 mM potassium phosphate buffer (pH 3) and methanol, followed by a second separation with water and acetonitrile to obtain pure compounds as either DIPEA or potassium salts as annotated by each compound..

### 2.3 Biological Evaluation

#### 2.3.1 PBP1b Inhibition Assays and *In Vitro* Antibacterial Activities

The study focused on enzyme inhibition of PBP1b from *S. pneumoniae*, as it is a well-established PBP model enzyme.^24^ It allows rapid biochemical evaluation and the generation of cocrystal structures with inhibitors. Enzyme inhibition was measured using the BOCILLIN FL assay following 30 min preincubation of PBP1b and compounds.^25,26^ Residual activity (RA) was measured at 100 μM, and IC_50_ values were determined for compounds with an RA lower than 10% at 100 μM concentration. Only compound **6** from the first series showed significant inhibition, as shown in Table 1. Further extension of the series of phenylacetic acid analogues yielded compound **11**, which is the most potent inhibitor of PBP1b with an IC_50_ of 42 nM. Almost all compounds showed inhibition in the submicromolar range, while some were in the low micromolar range. The exceptions were the benzoic acid derivatives (**2**–**5**, **7**), which showed only modest inhibition of PBP1b. PBP1b inhibition of most of the synthesized inhibitors was more potent than that of aztreonam (IC_50_ = 3.2 μM). To exclude possible effect of the counterion, i.e., potassium or DIPEA, on PBP1b inhibition, seven compounds (**9–10**, **12–16**) were tested with different counterions and no significant difference in inhibitory potencies were observed (Table 1). Larger electron-withdrawing groups at the *para* position were favored over smaller electron-donating groups. In the case of **12**–**14**, it can be seen that a derivative with *p*-bromo substituent has slightly stronger PBP1b inhibition and more potent antibacterial activity than *p*-chloro and *p*-fluoro derivatives. The addition of substituents at the α-carbon of phenylacetic acids in compounds **15** and **19** decreases the antibacterial activity, and in the case of **15**, even on-target potency. Electron-donating groups (**16**) reduce the antibacterial activity while the inhibition of PBP1b is maintained. Additionally, inhibition of *E. coli* PBP3 was evaluated at 150 μM after a 30 min preincubation, and residual activities were determined for all compounds using the thioester assay.^26^ Dose–response inhibition was then measured for five selected compounds (aztreonam, **6**, **11**, **24**, and **26**) that showed promising initial inhibition and represented good chemical diversity. Compound **11** displayed a lower IC_50_ value than compound **6**, consistent with trends observed for *S. pneumoniae* PBP1b (Figure S2). Notably, aztreonam and the 2D-designed analogues showed stronger inhibition than the smaller, simpler monobactams. IC_50_ values could not be determined for aztreonam, **24**, and **26** because they remained strongly inhibitory even at the lowest compound concentrations tested, with residual activities of 3.0% for aztreonam, 26% for **24**, and 52% for **26**.

**Table 1:** Inhibition of PBP1b from *S. pneumoniae* and PBP3 from *E. coli* by Monobactams **2**–**21** ^a^

compound	residual activity [%] or IC_50_ [μM] PBP1b	residual activity [%] or IC_50_ [μM] PBP3	compound	IC_50_ [μM] PBP1b	residual activity [%] or IC_50_ [μM] PBP3
**2**	30%	78%	**12b**	1.9 ± 0.19 μM	35%
**3**	34%	20%	**13a**	0.16 ± 0.02	6.4%
**4**	26%	78%	**13b**	0.22 ± 0.02	21%
**5**	45%	83%	**14a**	0.13 ± 0.03	12%
**6**	0.82 ± 0.10 μM	26.3 ± 2.8 μM	**14b**	0.24 ± 0.02	9.6%
**7**	27%	62%	**15a**	1.8 ± 0.18	24%
**8**	0.88 ± 0.09 μM	92%	**15b**	2.8 ± 0.28	38%
**9a**	0.33 ± 0.03 μM	29%	**16a**	0.36 ± 0.04	5.2%
**9b**	0.25 ± 0.02 μM	23%	**16b**	0.44 ± 0.04	12%
**10a**	0.138 ± 0.01 μM	27%	**17**	0.25 ± 0.02	3.8%
**10b**	0.145 ± 0.01 μM	47%	**18**	2.5 ± 0.25	21%
**11**	0.042 ± 0.004 μM	7.6 ± 0.7 μM	**19**	0.112 ± 0.01	2.9%
**12a**	0.31 ± 0.03 μM	11%	**21**	0.52 ± 0.05	9.3%

a Compounds obtained with both counterions were numbered as “a”—potassium and “b”—DIPEA counterion.

We determined the minimum inhibitory concentrations (MICs) of our compounds against 10 bacterial strains, including ESKAPE pathogens (*S. aureus*, MRSA, *E. coli*, *E. coli* D22, *E. coli* N43, *A. baumannii*, *E. faecalis*, *E. faecium*, *P. aeruginosa* and *K. pneumoniae*), as shown in Table S2. Compound **6** showed modest effect against *S. aureus*, which was also demonstrated for its phenylacetic acid analogues (**7**–**21**) and is unusual for monobactams. No inhibition of MRSA was observed for any of the compounds. The lack of inhibition may result from poor binding of the synthesized monobactams to PBP2a or from MRSA-specific cell wall characteristics that limit permeability and increase efflux.^27–29^ Compound **17** was the only compound from the first series that inhibited *E. coli* growth. Compounds **17**, **20** and **21** have been reported previously, but enzyme inhibition has not been determined and MICs have only been tested on a very limited number of bacterial strains.^30–32^ In the case of **20**, stability is a problem as it was reported that the side chain amino group can attack the β-lactam core intramolecularly and was therefore not biologically evaluated.^32^ Series designed by using a 2D similarity search showed, on average, more potent inhibition of PBP1b than aztreonam, as shown in Table 2. Compound **26** was the most promising inhibitor with MICs comparable to aztreonam and was most potent against *E. coli* and *K. pneumoniae*. Two compounds, **24** and **26**, showed stronger activity against *A. baumannii* compared to aztreonam, but were less effective against *P. aeruginosa*. As mentioned earlier, the free carboxylic acid in the side chain plays a crucial role in activity against *P. aeruginosa*.^33^ All oxime-containing compounds (**22**, **24**, **26**), including **27**, which contains alkene isostere of oxime, showed antimicrobial activity against Gram-negative bacteria, but none against Gram-positive bacteria (Table S3).

**Table 2:** Inhibition of PBP1b from *S. pneumoniae*, PBP3 from *E. coli* and Minimal Inhibitory Concentrations for New Monobactams.^a^ ^,^ ^b^

			MIC [μg/mL]						
Cpd.	IC_50_ PBP1b [μM]	residual activity [%] PBP3	*S. aureus*	*E. coli*	EC D22	EC N43	*A. baumannii*	*P. aeruginosa*	*K. pneumoniae*
***22***	*0.29 ± 0.03*	*0%*	*128*	*4*	*1*	*0.063*	*64*	*>128*	*2*
**23**	0.72 ± 0.07	66%	>128	>128	>128	>128	>128	>128	>128
***24***	*3.5 ± 0.3*	*26% RA at* *1 μM*	*>128*	*4*	*1*	*0.5*	*16*	*128*	*2*
**25**	5.9 ± 0.8	49%	>128	>128	>128	>128	>128	>128	>128
***26***	*0.41 ± 0.04*	*52% at* *1 μM*	*128*	*0.5*	*0.25*	*0.063*	*16*	*>128*	*0.25*
**27**	2.2 ± 0.2	0.3%	>128	16	4	1	>128	>128	4
aztreonam	3.2 ± 0.3	3.0% at μM	>128	0.5	0.5	1	32	16	0.25

a Includes bacterial strains: *E. coli* ATCC 25922, *A. baumannii* 8C6 GES-14, *K. pneumoniae* (RDK 070A; ATCC 51503), *P. aeruginosa* RDK 184 (DSM 939; ATCC 15442), *E. coli* N43 (CGSC no. 5583) and *E. coli* D22 (CGSC no. 5163).

b N.D.—not determined.

Strong target-based inhibition does not necessarily translate into potent antibacterial activity. This discrepancy may arise from limited permeation or active efflux by bacterial transport systems. Several strategies could be used to address these barriers, including siderophore conjugation or designing molecules in line with permeability guidelines such as eNTRy or PASsagE. However, such optimization requires a robust target inhibitor as the starting point, which remains the prerequisite for achieving antibacterial efficacy.^34,35^ *E. coli* D22 is a strain with a more permeable outer membrane, while *E. coli* N43 is a strain in which AcrA, a component of notorious efflux pumps, is knocked out. Based on the MICs of these two strains, we can determine whether our compounds have difficulties with either permeability or pump efflux. This was the case for compounds **17–18**, **22**, **24** and **26–27**, as the MIC values for these two strains were 2 to 16-fold lower than for the wild-type *E. coli*. It appears that for this set of compounds, membrane permeation is less of an obstacle than active efflux via bacterial efflux pumps.

To confirm the presence of potassium and sodium counterions, elemental analysis and atomic emission spectroscopy (AES) were performed on representative compounds (**5** and **11**). We observed a difference in MIC values for the same compounds with potassium or DIPEA counterions (compounds **9–10** and **12–16**) by one or two dilutions in favor of potassium (Table S2). As MIC values are reported in mass concentrations (μg/mL), higher mass of the counterion results in lower percentage of the active monobactam moiety in the tested mass. When recalculated to molar concentrations (Table S4), the difference was lower and can be attributed to experimental error.

### 2.4 Crystallographic Study

We have obtained several crystal structures of acyl-enzyme complexes with *S. pneumoniae* PBP1b* to elucidate the binding mode of our new monobactams (PDB ID: 9SG9 compound **6**, 9SG5 (**9**), 9SG6 (**10**), 9SG7 (**11**), 9SG8 (**15**), 9SGA (**21**), 9SGB (**22**), 9SGC (**24**), 9SGD (**26**), 9SGE (aztreonam)). This enzyme differs from the one used in our *in vitro* inhibition assays as it is a truncated variant and contains specific mutations, as previously described.^8^ We have successfully solved structures of complexes formed by the hydrolyzed compounds **6**, **9**, **10**, **11**, **15**, **21**, **22**, **24**, **26** and aztreonam with PBP1b*.

As shown in Figure 1, compound **22**, which contains an aminothiazole and an oxime moiety in the side chain, forms similar interactions to aztreonam. The aminothiazole moiety of aztreonam forms a water-bridged hydrogen bond with Thr655 in *S. pneumoniae* PBP1b*. In compound **22**, this hydrogen bond is not formed, but one is formed with Gly656, closely mimicking the binding orientation of aztreonam. The main difference lies in the orientation of the oxime side chain: in compound **22** it is it is preferentially directed toward the solvent and forms an additional water-bridged hydrogen bond with Met497. Notably, aztreonam bound to *E. coli* PBP1b (PDB ID: 5HLB)^21^ engages in two hydrogen bonds, one with the amide nitrogen of Asn703 and another with the hydroxyl group of Ser507. Interestingly, Gly656 in *S. pneumoniae* PBP1b* occupies a structurally analogous position to Asn703 in *E. coli* PBP1b, indicating a conserved interaction site despite species-specific variations. We observed a different orientation of the essential Thr654, part of the conserved KTGT motif, for both compound **22** and aztreonam, accompanied by two possible rotamers of Thr652. This highlights the structural adaptability of the PBP1b* catalytic site. Even for the larger monobactam derivatives with side chains more similar to those of aztreonam (**22**, **24**, **26**), we observed a residual electron density that could indicate the presence of a second conformation with low occupancy, as observed for the simpler monobactams described below.

**Figure 1:**
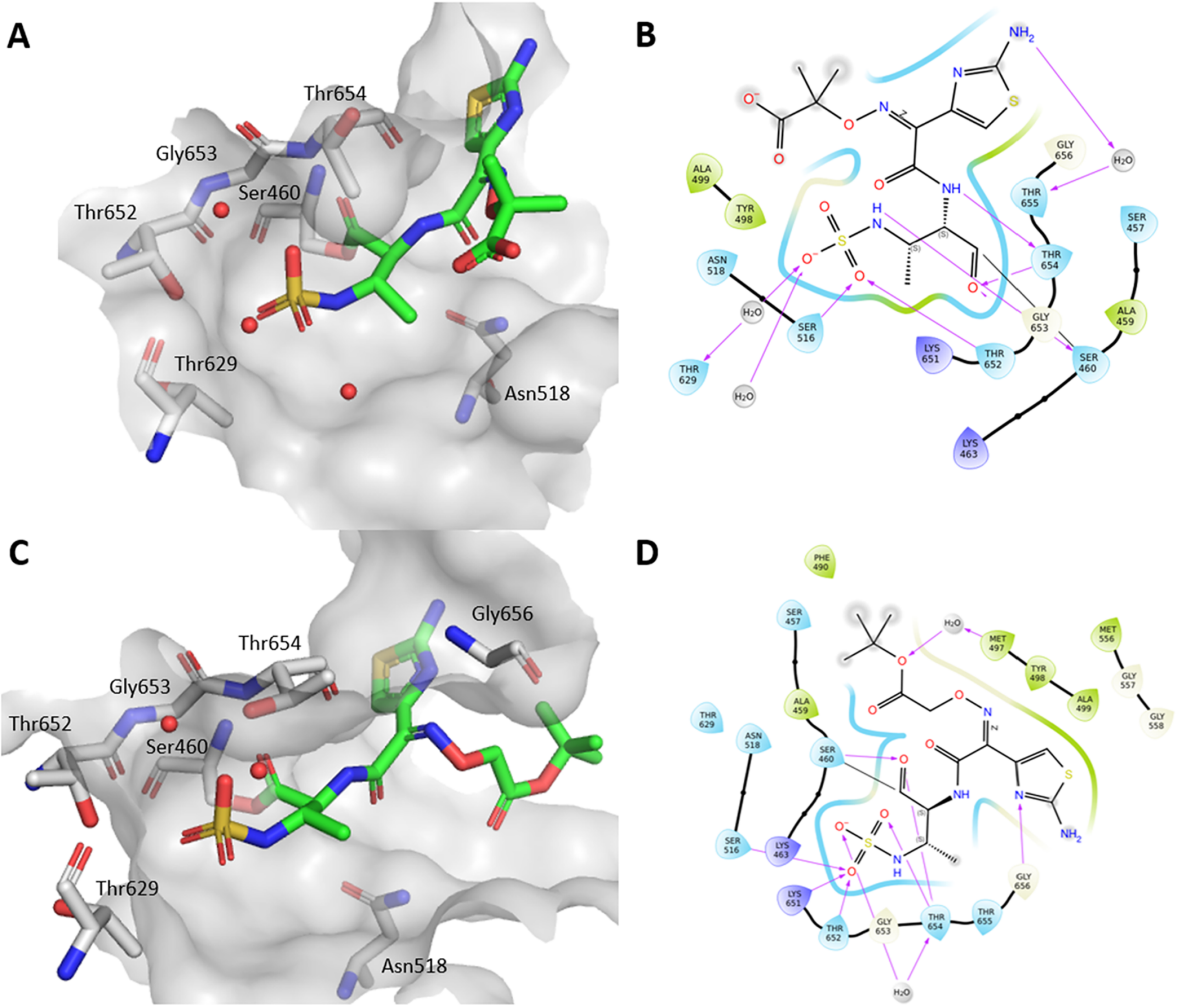
Binding poses of the complex formed by hydrolyzed aztreonam and compound **22** in the active site of PBP1b*. (A) 3D conformation of aztreonam in the active site, (B) 2D representation of aztreonam with interactions shown, (C) 3D conformation of compound **22** in the active site, (D) 2D representation of **22** with interactions shown. Covalent bonds with Ser460 are marked with a black line, hydrogen bonds with pink arrows, solvent exposed areas with gray highlight.

The compounds with an arylacetic acid side chain bind very similarly and all occupy a binding pose like aztreonam, with the only difference being the orientation of the aromatic ring. Monobactams **9**, **10**, **15** and **21** bind in two different conformations, with the orientation of the aminosulfonic acid moiety occupying two distinct binding conformations. In compound **9**, for example, one conformation of the aminosulfonic acid is stabilized directly by Ser516, Thr652 and Thr654, forming a total of 4 hydrogen bonds, as shown in Figure 2. In the other conformation, the aminosulfonic acid moiety forms one direct hydrogen bond with Thr654 and five water-bridged bonds with Ala499, Thr654, Thr655, Gly656 and Asp658. The aromatic ring is oriented toward the solvent and the side chain is similarly oriented in both conformations. The *O*-methoxy group of compounds **15** adopts a similar orientation to that of the oxime in aztreonam. However, the aromatic ring of **15** does not form any interactions with the binding site, so it is oriented differently from the aminothiazole in aztreonam. We hypothesize that the presence of two different conformations is due to the lack of interactions with the monobactam side chain, allowing the enzyme to open the ring from two different sides. Interestingly, these compounds show a stronger inhibition of PBP1b compared to aztreonam.

**Figure 2:**
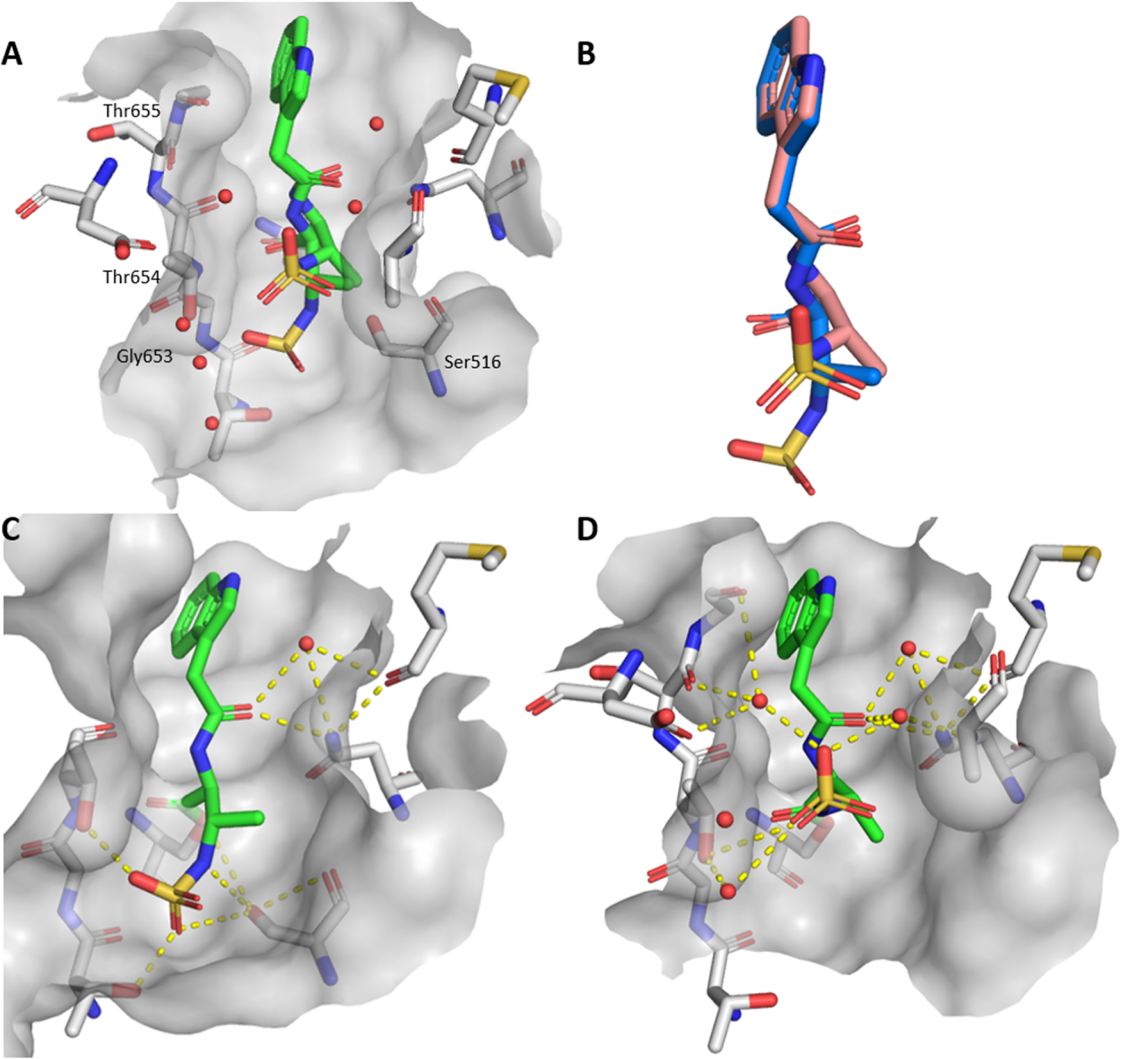
Two binding poses of the complex formed by hydrolyzed monobactam **9** in the active site of PBP1b*. (A) binding of both poses in the active site, (B) poses of bound ligand, (C and D) each pose showcasing hydrogen bonding in the active site.

We found two exceptions that form additional interactions within the monobactam side chain, as shown in Figure 3. Compound **10**, which contains a *p*-nitro group, forms a hydrogen bond with Ser553 via a hydroxyl group of the side chain, resulting in strong inhibition of the enzyme, and shows moderate MICs on *S. aureus*. Alignment of structures with aztreonam-bound PBPs was performed using Maestro (Schrödinger Suite, 2025–2; Schrödinger, LLC, New York, NY, 2025). The root-mean-square deviations (RMSDs) of the active sites compared to PBP1b* of *S. pneumoniae* bound to aztreonam are 1.00 Å for *E. coli* PBP1b (PDB ID: 5HLB), 1.59 Å for *P. aeruginosa* PBP3 (PDB ID: 3PBS), and 1.05 Å for *A. baumannii* PBP1a (PDB ID: 3UE0), indicating that the active sites are highly similar. We found that PBP1a from *A. baumannii* (PDB ID: 3UE0 ^36^) and PBP3 from *P. aeruginosa* (PDB ID: 3PBS^37^) have threonine residues at a similar position to Ser553 (PBP1b*, *S. pneumoniae*). This could be used for the design of simpler monobactams targeting the above-mentioned PBPs.

**Figure 3:**
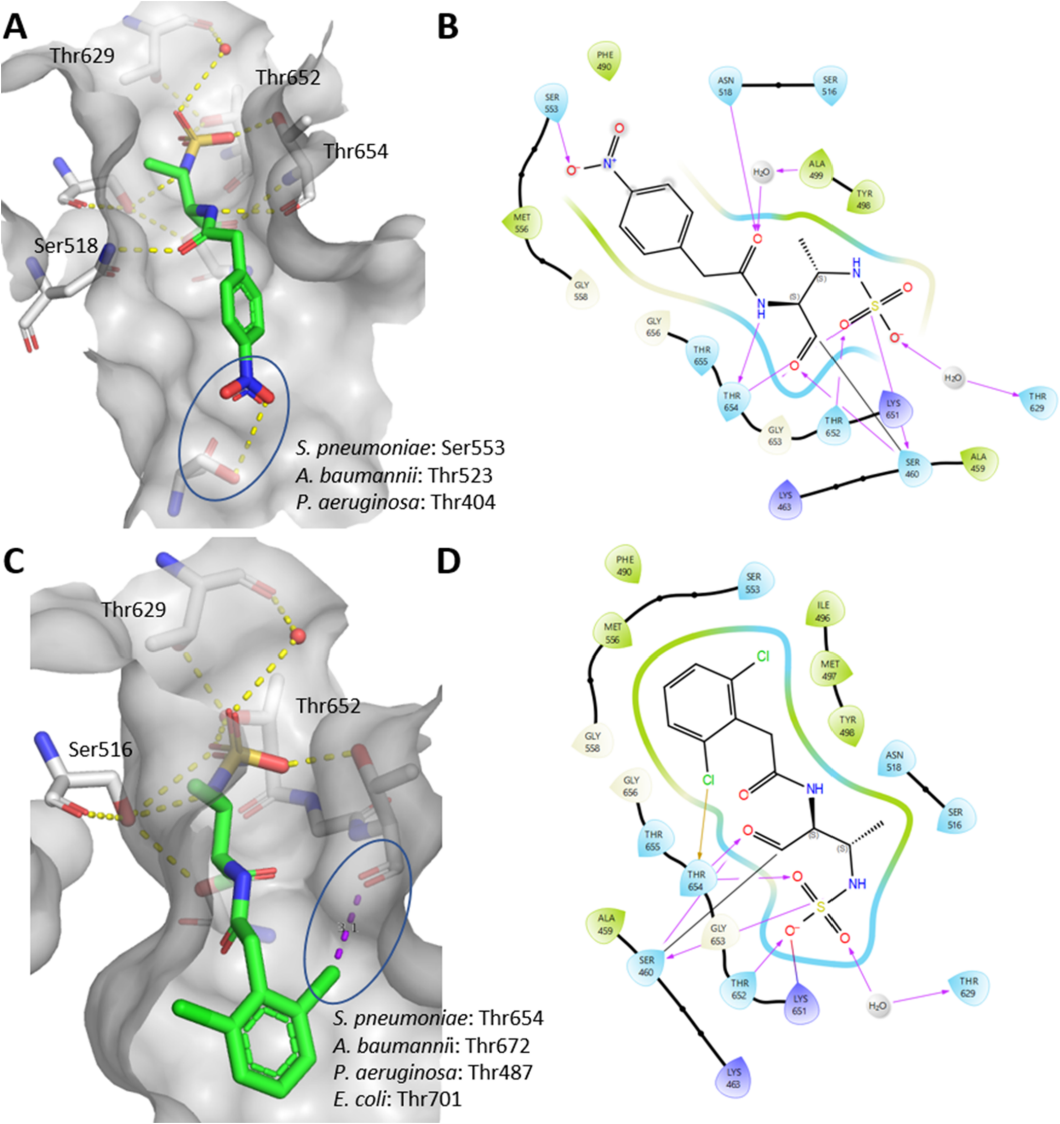
(A) Crystal structure of the complex formed by hydrolyzed compound **10** and PBP1b* from *S. pneumoniae*, with labeled amino acid residues of PBP1a from *A. baumannii* (PDB ID: 3UE0) and PBP3 from *P. aeruginosa* (PDB ID: 3PBS) located similarly to the serine involved in hydrogen bonding, (B) 2D representation of **10** with interactions shown, (C) Crystal structure of the complex formed by hydrolyzed compound **11** and PBP1b* from *S. pneumoniae* with labeled amino acid residues of PBP1a from *A. baumannii* (PDB ID: 3UE0), PBP3 from *P. aeruginosa* (PDB ID: 3PBS) and PBP1b from *E. coli* (PDB ID: 5HLB) located at the site of threonine forming the halogen bond, (D) 2D representation of **11** with interactions shown. Covalent bonds with Ser460 are marked with a black line, hydrogen bonds with pink arrows, halogen bond with a yellow arrow, solvent exposed areas with gray highlight.

In addition, we have identified a previously unknown halogen-bonding interaction of **11** formed by a 2,6-dichlorophenyl moiety with the backbone carbonyl of the essential Thr654, leading to a nanomolar inhibitor of PBP1b. Aztreonam is primarily known to inhibit PBP3 of *P. aeruginosa* and *E. coli*, but generally binds poorly to other PBPs such as PBP1b. The only PBP1b-specific β-lactam currently on the market is cefsulodin, which has a phenylacetic acid side chain with an additional sulfonic acid at an α-carbon, a structure quite similar to our inhibitors. Structural comparisons of PBP1a from *A. baumannii*, PBP3 from *P. aeruginosa* and PBP1b from *E. coli* with PBP1b from *S. pneumoniae* show that the threonine backbone carbonyl is positioned only 1.0 Å away in *A. baumannii* and 1.6 Å away in *E. coli* and *P. aeruginosa*. This suggests that the optimization of monobactams to form halogen bonds could enhance the inhibition of PBP1b and extend their spectrum of activity to other PBPs, especially since it involves a threonine that is a part of the conserved KTGT motif.

## 3 Conclusions

We have designed, synthesized and biologically evaluated a library of novel monobactams based on the commercially available compound **1**. More than 20 sub- or low-micromolar inhibitors of PBP1b from *S. pneumoniae* were synthesized, of which compound **11** proved to be the most potent (IC_50_ = 42 nM). Inhibitors **6** and **8**–**21**, which have an arylacetic acid side chain, showed potent inhibition of PBP1b and moderate antibacterial activity against Gram-positive bacteria. Compounds **22**–**27**, identified by 2D similarity screening based on the ATMO side chain, showed potent inhibition of PBP1b. Inhibitors **22**, **24** and **26** also showed strong antibacterial activity against most Gram-negative strains. We obtained nine crystal structure complexes formed by hydrolyzed novel monobactam inhibitors and PBP1b*, revealing the potential for additional hydrogen bonding interactions with the hydroxyl group of Ser553 or halogen bonding interactions with the backbone carbonyl of Thr654. As Thr654 is part of the conserved KTGT active site motif, these interactions could be utilized to enhance inhibition of a broad range of PBPs from different bacterial species. This is particularly important as, to our knowledge, only three different monobactams have been crystallized in complex with PBPs. Overall, our results provide new structural insights into monobactam binding and offer valuable guidance for future structure-based drug design efforts targeting PBPs.

## 4 Experimental Section

### 4.1 PBP1b Purification

We employed a vector expressing PBP1b from *S. pneumoniae* (designated pGEX-GST-PBP1b) to transform chemically competent *E. coli* NiCo21­(DE3) (obtained from New England Biolabs, USA), following previously established protocols.^8,26^ The transformed cells were cultured at 37 °C and 250 rpm in LB broth supplemented with 100 μg/mL ampicillin until reaching an OD_600_ of approximately 1. Expression of PBP1b was induced by adding 1 mM IPTG, and the culture was further incubated at 16 °C for an additional 20 h. After induction, cells were harvested by centrifugation (10 min at 3000*g*, 4 °C), and the resulting cell pellets were stored at −80 °C for subsequent purification steps. To purify PBP1b, the cell pellet was resuspended in buffer A (composed of 50 mM, Tris-HCl, 200 mM, NaCl, 1 mM EDTA, 1 mM DTT, pH 8.0) and lysed using sonication on ice. Cell debris were removed by centrifugation (30 min at 16,000*g*, 4 °C, repeated twice). The cleared lysate was loaded onto two interconnected 1 mL GSTrap HP columns (Cytiva, USA), which had been pre-equilibrated with buffer A. Following loading, the column was washed with buffer A, and the protein of interest (PBP1b) was eluted using buffer B (containing 50 mM Tris, 200 mM NaCl, mM EDTA, 1 mM DTT, 10 mM reduced glutathione, pH 8.0). The eluted PBP1b was subsequently transferred to buffer C (composed of 50 mM HEPES, 100 mM NaCl, 1 mM EDTA, and 10% glycerol, pH 7.0) through buffer exchange. Finally, the purified protein was concentrated using a 50-kDa molecular weight cutoff filter (Ultra-4 centrifugal filter units; Amicon), aliquoted, flash-frozen in liquid nitrogen, and stored at −80 °C. Protein purity was checked by SDS-PAGE, and the concentration was determined fluorometrically with Invitrogen Qubit (Thermo Fisher).

### 4.2 Assay for Inhibition of PBP1b

We investigated the inhibition of *S. pneumoniae* PBP1b using an assay with BOCILLIN FL, as reported previously.^26^ To measure fluorescence anisotropy, we employed 60 nM purified PBP1b and 30 nM BOCILLIN FL in a 100 mM sodium phosphate buffer (pH 7.0) containing 0.01% Triton X-100. The inclusion of Triton X-100 helped reduce promiscuous inhibitor detection and protein binding to the plate. The assay was conducted in triplicate, using a 50 μL volume in black flat-bottom, 384-well microplates at 30 °C. We quantified the change in fluorescence anisotropy using a Biotek Synergy H4 Hybrid microplate reader equipped with polarizing filters (excitation wavelength λ = 482 nm, emission wavelength λ = 530 nm). The calculated fluorescence anisotropy (FA) followed this equation: (FA) = (*F*_para_ – *F*_perp_)/(*F*_para_ + 2*F*_perp_), where (*F*_para_) represents the fluorescence intensity parallel to the excitation plane, and (*F*_perp_) represents the fluorescence intensity perpendicular to the excitation plane. Additionally, we determined residual activities by preincubating the test compound (100 μM) and the protein for 1 h at 30 °C before initiating the reaction with BOCILLIN FL. To assess residual activity, we compared the change in FA after 30 min to the uninhibited (1% v/v DMSO) control.

### 4.3 PBP3 Purification

A plasmid construct for *E. coli* PBP3 expression as a soluble fragment (residues 60–588), excluding the N-terminal transmembrane helix was used in these experiments.^38^ The construct was transformed into BL21­(DE3) Star.pRosetta and protein overexpression was carried out in auto induction 2-YT media (Formedium) using a bioreactor at room temperature for 24 h. The N-terminal His6-tagged enzyme was purified in 20 mM Tris-HCl (pH 8.0), 400 mM NaCl and 20 mM Imidazole by reverse Nickel affinity chromatography using recombinant HRV 3C protease for N-terminal His tag cleavage. The protein was subsequently injected into a 26/60 HiLoadTM Superdex 200 column (GE Healthcare) and eluted in 20 mM Tris-HCl (pH 8.0) and 400 mM NaCl. Pure fractions were combined and concentrated to 5.0 mg/mL.

### 4.4 PBP3 Inhibition Assay Using Ellman Reagent

Inhibition of *E. coli* PBP3 was determined spectrophotometrically by monitoring the formation of the 2-nitro-5-thiobenzoate anion (TNB^2–^). Residual activities were calculated based on the ability of each compound to inhibit the hydrolysis of the thioester substrate analog 2-{[(benzoyl-d-alanyl)-thio]-acetic acid}, following a previously described protocol.^39^

PBP3 (1 μM) was incubated with the test compound (final concentration 150 μM) in 10 mM sodium phosphate buffer (pH 7.0) containing 100 mM d-alanine, 0.01 mg/mL BSA, and 0.01% Triton X-100. The mixture was preincubated for 30 min at 25 °C. The reaction was then initiated by the addition of 5,5′-dithiobis­(2-nitrobenzoic acid) (DTNB, Ellman’s reagent) and the thioester substrate to yield final concentrations of 1 mM and 5 mM, respectively, in a total reaction volume of 150 μL. Triton X-100 was included to reduce the likelihood of detecting nonspecific (promiscuous) inhibition.

The initial rate of thioester hydrolysis was measured by monitoring absorbance at 412 nm for 30 min in 96-well microtiter plates using a BioTek Synergy H4 Hybrid microplate reader (BioTek Instruments, USA). Control reactions were performed in the absence of inhibitor, containing 1% (v/v) DMSO. All experiments were conducted in triplicate. Residual activity (RA) was calculated as the ratio of the reaction rate in the presence of inhibitor (*v*_i_) to the reaction rate in its absence (*v*_0_), expressed as a percentage according to the following equation: RA = [(*v*_i_ – *b*)/(*v*_0_ – *b*)] × 100, where *b* represents the blank value corresponding to the initial rate of spontaneous thioester hydrolysis in the presence of inhibitor but without PBP3. IC_50_ values were determined by measuring reaction rates across seven inhibitor concentrations and fitting the data using a four-parameter nonlinear regression model in GraphPad Prism 10.5.0 (GraphPad Inc., USA).

### 4.5 Bacterial Susceptibility Test

Minimum inhibitory concentrations (MICs) were assessed using broth microdilution method in 96-well U plates, following the guidelines set by the Clinical and Laboratory Standards Institute (CLSI) and the European Committee on Antimicrobial Susceptibility Testing. We prepared bacterial suspensions of specific strains including *S. aureus* ATCC 29213, MRSA QA-11.7, *E. coli* ATCC 25922, *A. baumannii* 8C6 GES-14 (strain obtained from a European reference laboratory, EURL-AMR, DTU, Copenhagen, Denmark), *K. pneumoniae* (RDK 070A; ATCC 51503), *P. aeruginosa* RDK 184 (DSM 939; ATCC 15442), *E. faecalis* ATCC 29212, *E. faecium* *(30088/46)*, *E. coli* N43 (CGSC no. 5583) and *E. coli* D22 (CGSC no. 5163) corresponding to the 0.5-McFarland turbidity standard. These suspensions were then diluted with cation-adjusted Mueller-Hinton broth containing TES to achieve an end inoculum of 5 × 10^5^ CFU/mL for the assay. The compounds, dissolved in DMSO, were mixed with the bacterial inoculum and incubated at 35 °C for 18–24 h. The MIC values were determined visually as the lowest dilution of the compounds that did not exhibit turbidity. Tetracycline served as a positive control on each test plate, and all experiments were conducted in duplicate.

### 4.6 PBP1b* Purification and Crystallization

PBP1b* was purified mostly as previously described.^24^ PBP1b* crystals were grown by the vapor diffusion method at 20 °C using a hanging-drop setup. PBP1b* was crystallized by mixing 1 μL of protein sample (5–7 mg/mL, 20 mM HEPES pH 7.0, 100 mM NaCl, 1 mM EDTA) and 1 μL of reservoir solution (3 M NaCl, 0.6–0.9 M ammonium sulfate, 50 mM HEPES pH 7.2). Crystal soaks were performed by slowly adding ligands prepared in DMSO (to a final concentration of 5%) directly to the crystallization drop. Crystals were mounted in cryo loops and flash-frozen under liquid nitrogen.

### 4.7 Data Collection and Structure Solution

All data were collected under a cold nitrogen stream at 100 K at the European Synchrotron Radiation Facility (Grenoble) on beamline MASSIF-1 (ID30A-1),^40^ and were indexed and scaled using the XDS program package.^41^ ADXV^42^ and XDSGUI^43^ were used to perform data quality analysis and STARANISO^44^ was used for resolution cutoff check-up.^45,46^ The reduced X-ray diffraction data sets were imported into the CCP4–8.0 program suite.^47^ Structures were solved by molecular replacement using PHASER^48^ and employing the coordinates of the unliganded PBP1b* (PDB: 2BG1)^8^ lacking residues 460, 516–518 and 650–661 as a search model. The models were then rebuilt *de novo* to remove bias using ARP/wARP 8.0.^49^ The structures were completed by cycles of manual model building with COOT 0.8.9.2.^50^ Water molecules were added to the residual electron density map as implemented in ARP/wARP and COOT. Ligand restraints libraries were generated with JLigand.^51^ Crystallographic macromolecular refinement was performed with REFMAC 5.8.^52^ Several cycles of manual model building and refinement were performed until *R*_work_ and *R*_free_ converged. At this point TLS definition^53^ was determined and validated using the TLSMD and PARVATI servers.^54,55^ The stereochemical quality of the refined models was verified with MOLPROBITY,^56^ as implemented in COOT, and with PROCHECK.^57^ Due to the high concentration of NaCl employed in crystallization as well as optimal H-bonding parameters, Cl^–^ ions were modeled into spheres of high electron density.^58^ Secondary structure assignment was performed by DSSP^59^ and STRIDE.^60^ Data collection and refinement statistics are included in Table S5. All solved structures displayed between 99.0 and 99.3% of the nonglycine residues in the most favored and allowed regions of the Ramachandran plot. Figures displaying protein structures were generated with PyMOL Molecular Graphics System, Version 3.1 Schrödinger, LLC. Final refined model coordinates and structure factors have been deposited in the Protein Data Bank (PDB, http://www.rcsb.org), ID codes: 9SG5, 9SG6, 9SG7, 9SG8, 9SG9, 9SGA, 9SGB, 9SGC, 9SGD, 9SGE. Authors will release the atomic coordinates and experimental data upon article publication (https://doi.esrf.fr/10.15151/ESRF-DC-2258945928).

### 4.8 Computational Design

#### 4.8.1 Library Formation and Structure-Based Pharmacophore Models

KNIME^22^ and MarvinSketch (Marvin 23.12.0, 2023, ChemAxon http://www.chemaxon.com) were used to generate monobactams bearing amides with our in house library of carboxylic acids. This was then screened against structure-based pharmacophore models (as depicted in Figure 4) using LigandScout 4.4.9 (Inte:Ligand GmbH.).^23^ A selection of compounds that were found as hits in all three models were then synthesized, as described below. The pharmacophore fit and corresponding interactions of the synthesized compounds across all three models are presented in Figure S1 and combined scores are presented in Table S1.

**Figure 4:**
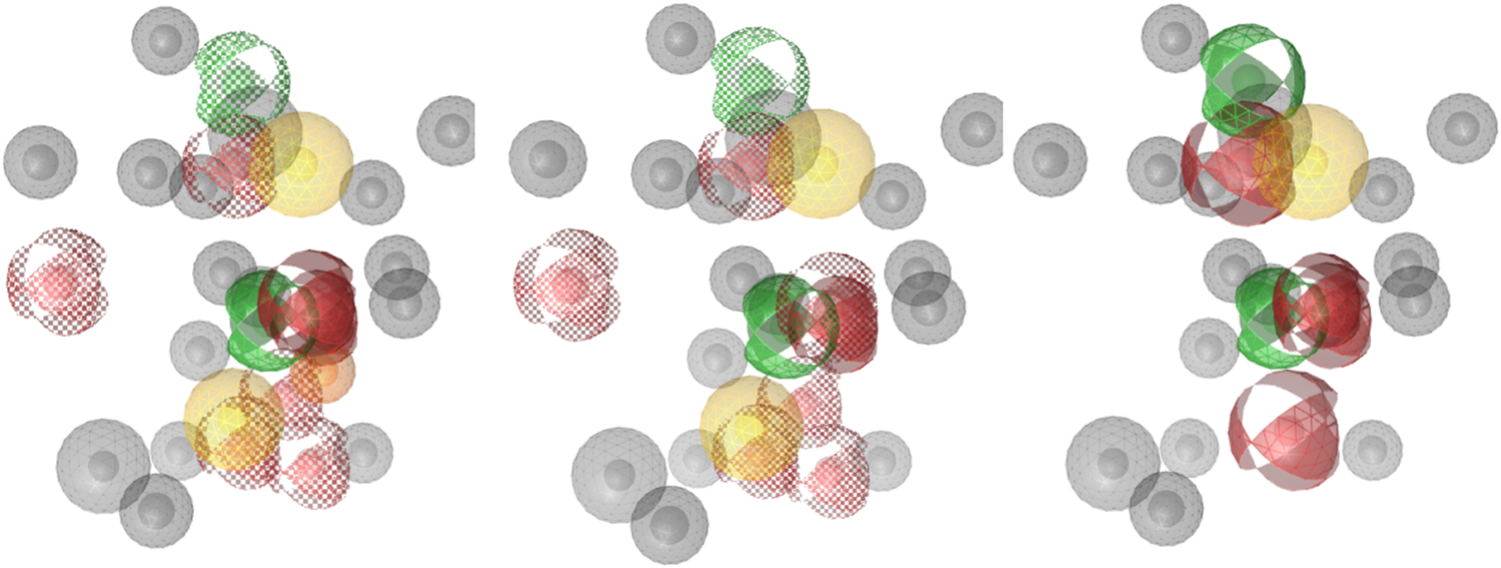
Pharmacophore models used for screening. They were based of aztreonam in complex with PBP1b of *E. coli* (PDB ID: 5HLB), with different pharmacophores used as optional, with and without binding point pharmacophore.

#### 4.8.2 2D Fingerprint-Based (FP2 Fingerprints) Similarity Search

We selected ATMO side chain, present on aztreonam’s C3 spot to screen against library of commercially available acids. The screening was performed using OpenBabel 3.1.1. and based on the Tanimoto index. We obtained 20 hits above our cutoff value (>0.4) but focused on the top scored on Tanimoto index. We assessed the in-stock availability of compounds and subsequently purchased 6 diverse carboxylic acids.

### 4.9 Chemistry

Reagents and solvents were purchased from commercial suppliers and were used without further purification. Reaction progress was monitored using analytical thin layer chromatography (TLC) performed on Silica Gel 60F_254_ plates (Merck) or via LC–MS analyses performed on an Agilent Technologies 1260 Infinity II LC System (Agilent Technologies, Inc., Santa Clara, CA, USA) coupled to an ADVION expression CMSL mass spectrometer (Advion Inc., Ithaca, USA) fitted with Waters XBridge C18 column (3.5 μm, 4.6 mm × 150 mm); solvent A: 0.1% formic acid (v/v) in HPLC-grade water, solvent B: 1% acetonitrile (v/v) in HPLC-grade water. HRMS were recorded on a Thermo Scientific Q Exactive mass spectrometer (Thermo Fisher Scientific Inc., Waltham, USA). ^1^H and ^13^C NMR spectra were recorded at 400 and 101 MHz, respectively, on a Bruker AVANCE III 400 spectrometer (Bruker Corporation, Billerica, MA, USA) in solvents indicated, using Me_4_Si (TMS) as an internal standard. The general method used a Waters Acquity UPLC BEH C18 column (2.1 × 50 mm, 1.7 μm) thermostated at 40 °C, with injection volume, 1 μL; sample, 0.1–0.3 mg/mL in 0.1% TFA in water; flow rate, 0.4 mL/min; detector λ, 220 and 254 nm; mobile phase A: 0.1% TFA (v/v) in water; mobile phase B: MeCN. Gradient: 0–2 min, 2% B, 2–5 min, 2%–90% B; 5–8 min, 90% B. Purity of all compounds is >95% by HPLC analysis unless otherwise indicated. AES was performed by dissolving samples in 50 mL of Milli-Q water and further diluted 5- or 10-fold, depending on the expected ion concentration. Subsequently, 1 mL of hydrochloric acid was added to each solution. Emission intensities were measured using a flame (acetylene/air) at 589.2 nm for sodium and 766.5 nm for potassium. Calibration curves for both ions were generated over a concentration range of 1–4 mg/L.

#### 4.9.1 General Procedure 1 (GP1)—RP-CC Purification

Compounds were purified using reversed-phase column chromatography (RP-CC) (Isolera Biotage One Flash Chromatography system or puriFlash 5.250 LC system from Advion Interchim Scientific; Biotage Sfär C18 Duo 100 Å 30 μm column, 30 g) using a gradient of 50 mM KH_2_PO_4_ buffer pH = 3 in deionized water and methanol as eluent (gradient 0% MeOH 1 column volume (45 mL), 0–60% MeOH in 8 column volumes (360 mL)). Fractions were combined and the solvent was evaporated *in vacuo*, to afford product mixture with KH_2_PO_4_, which was removed with a second reversed-phase column chromatography on the system mentioned above, using a gradient of deionized water and acetonitrile as eluent (gradient 0% MeCN 1 column volume (45 mL), 0–100% MeCN in 8 column volumes (360 mL)). Fractions were combined and the solvent was evaporated *in vacuo*, to afford pure product as either DIPEA or potassium salts as annotated by each compound.

#### 4.9.2 General Procedure 2 (GP2)—Amide Formation Using TBTU

Carboxylic acid (1.0 mmol, 1.0 equiv) was dissolved in tetrahydrofuran (THF) (10 mL per mmol) under argon, then TBTU (464 mg, 1.5 mmol, 1.5 equiv) and DIPEA (692 μL, 4.0 mmol. 4.0 equiv) were added. After 15 min, (2*S*,3*S*)-3-amino-2-methyl-4-oxoazetidine-1-sulfonic acid **1** (180 mg, 1.0 mmol, 1.0 equiv) was added, and the reaction was stirred for 16 h. Then, the solvent was removed under reduced pressure and the compound was purified by reversed-phase column chromatography, as described in GP1.

#### 4.9.3 General Procedure 3 (GP3)—Formation of Amide Using CDI

To a stirred solution of carboxylic acid (1.2 mmol, 1.2 equiv) in THF (10 mL per mmol) or DMF (5 mL per mmol), 1,1′-carbonyldiimidazole (CDI) was added (178 mg, 1.1 mmol, 1.1 equiv) and stirred for 1 h at room temperature. Subsequently, (2*S*,3*S*)-3-amino-2-methyl-4-oxoazetidine-1-sulfonic acid **1** (180 mg, 1.0 mmol, 1.0 equiv) and DIPEA (346 μL, 2.0 mmol, 2.0 equiv) were added and the reaction was stirred for 16 h at 50 °C. Then, the solvent was removed under reduced pressure and the compound was purified by reversed-phase column chromatography, as described in GP1.

#### 4.9.4 Potassium (2*S*,3*S*)-3-(6-Aminonicotinamido)-2-methyl-4-oxoazetidine-1-sulfonate (**2**)

Potassium (2*S*,3*S*)-3-(6-aminonicotinamido)-2-methyl-4-oxoazetidine-1-sulfonate was prepared following GP2 from 6-aminonicotinic acid (138 mg, 1.0 mmol) and purified following GP1. That afforded the desired product **2** as a white solid (67 mg, 19.8%). ^1^H NMR (400 MHz, DMSO-*d*_6_) δ 1.40 (d, *J* = 6.1 Hz, 3H), 3.75 (qd, *J* = 6.1, 2.7 Hz, 1H), 4.57 (dd, *J* = 8.2, 2.7 Hz, 1H), 6.96 (d, *J* = 9.4 Hz, 1H), 8.25 (dd, *J* = 9.3, 2.2 Hz, 1H), 8.46 (d, *J* = 2.2 Hz, 1H), 9.31 (d, *J* = 8.2 Hz, 1H); ^13^C NMR (101 MHz, DMSO-*d*_6_) δ 18.04, 57.02, 60.80, 112.66, 112.67, 118.03, 138.33, 141.50, 155.09, 162.44, 162.48; HRMS: (ESI−), *m*/*z* calc. for C_10_H_11_O_5_N_4_S [M–H]^−^ 299.04556, found 299.04510.

#### 4.9.5 Potassium (2*S*,3*S*)-3-(2-Bromo-3-nitrobenzamido)-2-methyl-4-oxoazetidine-1-sulfonate (**3**)

Potassium (2*S*,3*S*)-3-(2-bromo-3-nitrobenzamido)-2-methyl-4-oxoazetidine-1-sulfonate was prepared following GP2 from 2-bromo-3-nitrobenzoic acid (246 mg, 1.0 mmol) and purified following GP1. That afforded the desired product **3** as a pale-yellow solid (26 mg, 5.9%). ^1^H NMR (400 MHz, D_2_O) δ 1.61 (d, *J* = 6.2 Hz, 3H), 4.35 (dd, *J* = 6.2, 2.8 Hz, 1H), 4.69 (d, *J* = 2.8 Hz, 1H), 7.66 (t, *J* = 7.8 Hz, 1H), 7.73 (dd, *J* = 7.7, 1.7 Hz, 1H), 8.01 (dd, *J* = 8.1, 1.7 Hz, 1H); ^13^C NMR (101 MHz, D_2_O) δ 16.85, 58.41, 61.54, 110.95, 127.04, 129.29, 131.82, 139.03, 150.21, 165.04, 169.47; HRMS: (ESI−), *m*/*z* calc. for C_11_H_9_O_7_N_3_SBr [M–H]^−^ 405.93391, found 405.93480.

#### 4.9.6 Potassium (2*S*,3*S*)-3-(4-Azidobenzamido)-2-methyl-4-oxoazetidine-1-sulfonate (**4**)

Potassium (2*S*,3*S*)-3-(4-azidobenzamido)-2-methyl-4-oxoazetidine-1-sulfonate was prepared following GP2 from 4-azidobenzoic acid (163 mg, 1.0 mmol) and purified following GP1. That afforded the desired product **4** as a brown solid (64 mg, 17.6%). ^1^H NMR (400 MHz, D_2_O) δ 1.57 (d, *J* = 6.1 Hz, 3H), 4.26 (qd, *J* = 6.1, 2.8 Hz, 1H), 4.55 (d, *J* = 2.9 Hz, 1H), 7.00–7.06 (m, 2H), 7.67–7.73 (m, 2H); ^13^C NMR (101 MHz, D_2_O) δ 16.91, 58.50, 61.90, 119.01, 128.23, 129.05, 144.04, 166.10, 169.05; HRMS: (ESI−), *m*/*z* calc. for C_11_H_10_O_5_N_5_S [M–H]^−^ 324.04081, found 324.04051.

#### 4.9.7 Sodium (2*S*,3*S*)-3-(6-Hydroxynicotinamido)-2-methyl-4-oxoazetidine-1-sulfonate (**5**)

Sodium (2*S*,3*S*)-3-(6-hydroxynicotinamido)-2-methyl-4-oxoazetidine-1-sulfonate was prepared following GP2 from 6-hydroxynicotinic acid (139 mg, 1.0 mmol) and purified following GP1. Next, we added 200 mg of Dowex 50WX8 Na^+^ form to a water solution of our product. After stirring for 24 h, we filtered the reaction mixture and evaporated the solvent under reduced pressure. That afforded the desired product **5** as colorless oil (73 mg, 22.6%). ^1^H NMR (400 MHz, D_2_O) δ 1.56 (d, *J* = 6.2 Hz, 3H), 4.28 (qd, *J* = 6.2, 2.8 Hz, 1H), 4.56 (d, *J* = 2.9 Hz, 1H), 6.63 (dd, *J* = 9.6, 0.7 Hz, 1H), 7.97 (dd, *J* = 9.6, 2.7 Hz, 1H), 8.11 (dd, *J* = 2.7, 0.7 Hz, 1H); ^13^C NMR (101 MHz, D_2_O) δ 16.83, 58.49, 61.76, 114.16, 118.89, 137.79, 140.52, 164.99, 166.05, 166.53; HRMS: (ESI−), *m*/*z* calc. for C_10_H_10_O_6_N_3_S [M–H]^−^ 300.02848, found 300.03003; AES: Na calculated 7.11%, determined 7.5%; elemental analysis: calcd. for hydrate C_10_H_12_N_3_NaO_7_S: C, 35.20; H, 3.54; N, 12.31; determined C, 35.61; H, 3.38; N, 12.30.

#### 4.9.8 Potassium (2*S*,3*S*)-3-(2-(3-Hydroxyphenyl)­acetamido)-2-methyl-4-oxoazetidine-1-sulfonate (**6**)

Potassium (2*S*,3*S*)-3-(2-(3-hydroxyphenyl)­acetamido)-2-methyl-4-oxoazetidine-1-sulfonate was prepared following GP2 from 2-(3-hydroxyphenyl)­acetic acid (152 mg, 1.0 mmol) and purified following GP1. That afforded the desired product **6** as a yellow solid (14 mg, 4.0%). ^1^H NMR (400 MHz, D_2_O) δ 1.49 (d, *J* = 6.2 Hz, 3H), 3.58 (s, 2H), 4.13 (qd, *J* = 6.2, 2.8 Hz, 1H), 4.41 (d, *J* = 2.8 Hz, 1H), 6.79–6.88 (m, 3H), 7.27 (t, *J* = 7.8 Hz, 1H); ^13^C NMR (101 MHz, D_2_O) δ 16.74, 41.72, 58.59, 61.45, 114.29, 116.04, 121.33, 130.29, 136.21, 155.72, 165.92, 174.69; HRMS: (ESI−), *m*/*z* calc. for C_12_H_13_O_6_N_2_S [M–H]^−^ 313.04998, found 313.04968.

#### 4.9.9 Potassium (2*S*,3*S*)-3-(4-Amino-3-fluorobenzamido)-2-methyl-4-oxoazetidine-1-sulfonate (**7**)

Potassium (2*S*,3*S*)-3-(4-amino-3-fluorobenzamido)-2-methyl-4-oxoazetidine-1-sulfonate was prepared following GP2 from 4-amino-3-fluorobenzoic acid (155 mg, 1.0 mmol) and purified following GP1. That afforded the desired product **7** as an off-white solid (60 mg, 16.9%). ^1^H NMR (400 MHz, D_2_O) δ 1.55 (d, *J* = 6.2 Hz, 3H), 4.25 (qd, *J* = 6.2, 2.9 Hz, 1H), 4.52 (d, *J* = 2.8 Hz, 1H), 6.86 (t, *J* = 8.7 Hz, 1H), 7.32–7.41 (m, 2H); ^13^C NMR (101 MHz, D_2_O) δ 16.86, 58.60, 61.91, 114.42 (d, *J* = 20.2 Hz), 116.70 (d, *J* = 4.3 Hz), 121.70 (d, *J* = 6.1 Hz), 124.41 (d, *J* = 2.8 Hz), 139.34 (d, *J* = 13.1 Hz), 150.73 (d, *J* = 239.3 Hz), 166.38, 168.91 (d, *J* = 2.3 Hz); HRMS: (ESI−), *m*/*z* calc. for C_11_H_11_O_5_N_3_SF [M–H]^−^ 316.04089, found 316.04050.

#### 4.9.10 (2*S*,3*S*)-3-(2-(2-Aminothiazol-4-yl)­acetamido)-2-methyl-4-oxoazetidine-1-sulfonic acid, DIPEA Salt (**8**)

(2*S*,3*S*)-3-(2-(2-Aminothiazol-4-yl)­acetamido)-2-methyl-4-oxoazetidine-1-sulfonic acid DIPEA salt was prepared following GP3 from (2-aminotiazol-4-yl)­acetic acid (189 mg, 1.2 mmol) and purified following GP1. That afforded the desired product **8** as a white solid (19 mg, 4.2%). ^1^H NMR (400 MHz, D_2_O) δ 1.50 (d, *J* = 6.2 Hz, 3H), 3.67 (s, 2H), 4.17 (qd, *J* = 6.0, 3.1 Hz, 1H), 4.41–4.43 (m, 1H), 6.60 (s, 1H); ^13^C NMR (101 MHz, D_2_O) δ 16.74, 54.33, 58.38, 61.47, 105.91, 134.56, 165.81, 170.46, 170.93; HRMS: (ESI-), *m*/*z* calc. for C_9_H_11_O_5_N_4_S_2_ [M–H]^−^ 319.01763, found 319.01794.

#### 4.9.11 Potassium (2*S*,3*S*)-3-(2-(1*H*-Indol-3-yl)­acetamido)-2-methyl-4-oxoazetidine-1-sulfonate (**9**)

Potassium (2*S*,3*S*)-3-(2-(1*H*-indol-3-yl)­acetamido)-2-methyl-4-oxoazetidine-1-sulfonate was prepared following GP3 from 2-(1-*H*-indol-3-yl)­acetic acid (210 mg, 1.2 mmol) and purified following GP1. That afforded the desired product **9** as an off-white solid (111 mg, 29.7%). ^1^H NMR 400 MHz, D_2_O δ 1.37 (d, *J* = 6.2 Hz, 3H), 3.66 (d, *J* = 0.7 Hz, 2H), 4.04 (qd, *J* = 6.2, 2.8 Hz, 1H), 4.19 (d, *J* = 2.9 Hz, 1H), 7.10 (ddd, *J* = 8.0, 7.0, 1.1 Hz, 1H), 7.17–7.23 (m, 2H), 7.45 (dt, *J* = 8.1, 0.9 Hz, 1H), 7.50 (dt, *J* = 7.9, 1.1 Hz, 1H); ^13^C NMR (101 MHz, MeOD) δ 13.16, 17.28, 18.28, 18.72, 33.73, 33.77, 43.80, 55.85, 59.93, 59.96, 62.83, 62.93, 109.01, 112.32, 112.37, 119.35, 119.99, 122.56, 124.98, 128.56, 138.11, 165.62, 175.08; HRMS: (ESI−), *m*/*z* calc. for C_14_H_14_O_5_N_3_S [M–H]^−^ 336.06596, found 336.06633.

#### 4.9.12 Potassium (2*S*,3*S*)-2-Methyl-3-(2-(4-nitrophenyl)­acetamido)-4-oxoazetidine-1-sulfonate (**10**)

Potassium (2*S*,3*S*)-2-methyl-3-(2-(4-nitrophenyl)­acetamido)-4-oxoazetidine-1-sulfonate was prepared following GP3 from 2-(4-nitrophenyl)­acetic acid (224 mg, 1.2 mmol) and purified following GP1. That afforded the desired product **10** as a light-yellow solid (70 mg, 18.0%). ^1^H NMR (400 MHz, DMSO-*d*_6_) δ 1.35 (d, *J* = 6.1 Hz, 3H), 3.58 (qd, *J* = 6.1, 2.6 Hz, 1H), 3.64 (s, 2H), 4.33 (dd, *J* = 8.0, 2.6 Hz, 1H), 7.51–7.55 (m, 2H), 8.16–8.20 (m, 2H), 9.02 (d, *J* = 8.0 Hz, 1H); ^13^C NMR (101 MHz, DMSO**-** *d*_6_) δ 18.02, 41.47, 57.32, 60.67, 123.34, 130.47, 143.99, 146.32, 162.64, 169.20; HRMS: (ESI−), *m*/*z* calc. for C_12_H_12_O_7_N_3_S [M–H]^−^ 342.04014, found 342.04016.

#### 4.9.13 Potassium (2*S*,3*S*)-3-(2-(2,6-Dichlorophenyl)­acetamido)-2-methyl-4-oxoazetidine-1-sulfonate (**11**)

Potassium (2*S*,3*S*)-3-(2-(2,6-dichlorophenyl)­acetamido)-2-methyl-4-oxoazetidine-1-sulfonate was prepared following GP3 from 2-(2,6-dichlorophenyl)­acetic acid (246 mg, 1.2 mmol) and purified following GP1. That afforded the desired product **11** as a white solid (38 mg, 9.5%). ^1^H NMR (400 MHz, DMSO-*d*_6_) δ 1.35 (d, *J* = 6.2 Hz, 3H), 3.55 – 3.63 (m, 1H), 3.82 (s, 2H), 4.30–4.34 (m, 1H), 7.31 (dd, *J* = 8.6, 7.5 Hz, 1H), 7.45 (d, *J* = 8.1 Hz, 2H), 8.97 (d, *J* = 7.8 Hz, 1H); ^13^C NMR (101 MHz, DMSO-*d*_6_) δ 18.01, 37.22, 57.33, 60.81, 128.11, 129.34, 132.18, 135.53, 162.63, 167.80; HRMS: (ESI−), *m*/*z* calc. for C_12_H_11_O_5_N_2_Cl_2_S [M–H]^−^ 364.97712, found 364.97718; AES: K calculated 9.65%, determined 8.9%; Elemental analysis: calcd. for C_12_H_11_Cl_2_KN_2_O_5_S: C, 35.56; H, 2.74; N, 6.91; determined C, 35.51; H, 2.37; N, 6.71.

#### 4.9.14 Potassium (2*S*,3*S*)-3-(2-(4-Fluorophenyl)­acetamido)-2-methyl-4-oxoazetidine-1-sulfonate (**12**)

Potassium (2*S*,3*S*)-3-(2-(4-fluorophenyl)­acetamido)-2-methyl-4-oxoazetidine-1-sulfonate was prepared following GP3 from 2-(4-fluorophenyl)­acetic acid (185 mg, 1.2 mmol) and purified following GP1. That afforded the desired product **12** as a white solid (112 mg, 31.7%). ^1^H NMR (400 MHz, DMSO-*d*_6_) δ 1.34 (d, *J* = 6.1 Hz, 3H), 3.44 (s, 2H), 3.57 (qd, *J* = 6.1, 2.6 Hz, 1H), 4.32 (dd, *J* = 8.0, 2.6 Hz, 1H), 7.09–7.16 (m, 2H), 7.25–7.31 (m, 2H), 8.90 (d, *J* = 8.1 Hz, 1H); ^13^C NMR (101 MHz, DMSO-*d*_6_) δ 18.03, 40.90, 57.33, 60.63, 114.93 (d, *J* = 21.1 Hz), 130.87 (d, *J* = 8.0 Hz), 132.10 (d, *J* = 3.1 Hz), 161.05 (d, *J* = 242.0 Hz), 162.80, 170.21; ^19^F NMR (376 MHz, DMSO-*d*_6_) δ 45.79; HRMS: (ESI−), *m*/*z* calc. for C_12_H_12_O_5_N_2_FS [M–H]^−^ 315.04564, found 315.04570.

#### 4.9.15 Potassium (2*S*,3*S*)-3-(2-(4-Chlorophenyl)­acetamido)-2-methyl-4-oxoazetidine-1-sulfonate (**13**)

Potassium (2*S*,3*S*)-3-(2-(4-chlorophenyl)­acetamido)-2-methyl-4-oxoazetidine-1-sulfonate was prepared following GP3 from 2-(4-chlorophenyl)­acetic acid (205 mg, 1.2 mmol) and purified following GP1. That afforded the desired product **13** as a white solid (118 mg, 31.9%). ^1^H NMR (400 MHz, DMSO-*d*_6_) δ 1.34 (d, *J* = 6.2 Hz, 3H), 3.45 (s, 2H), 3.56 (qd, *J* = 6.1, 2.5 Hz, 1H), 4.32 (dd, *J* = 8.0, 2.6 Hz, 1H), 7.25–7.31 (m, 2H), 7.33–7.39 (m, 2H), 8.92 (d, *J* = 8.1 Hz, 1H); ^13^C NMR (101 MHz, DMSO-*d*_6_) δ 18.02, 41.04, 57.34, 60.61, 128.15, 130.93, 131.15, 134.96, 162.69, 169.91; HRMS: (ESI−), *m*/*z* calc. for C_12_H_12_O_5_N_2_ClS [M–H]^−^ 331.01609, found 331.01639.

#### 4.9.16 Potassium (2*S*,3*S*)-3-(2-(4-Bromophenyl)­acetamido)-2-methyl-4-oxoazetidine-1-sulfonate (**14**)

Potassium (2*S*,3*S*)-3-(2-(4-bromophenyl)­acetamido)-2-methyl-4-oxoazetidine-1-sulfonate was prepared following GP3 from 2-(4-bromophenyl)­acetic acid (205 mg, 1.2 mmol) and purified following GP1. That afforded the desired product **14** as a white solid (22 mg, 5.4%). ^1^H NMR (400 MHz, D_2_O) δ 1.48 (d, *J* = 6.2 Hz, 3H), 3.61 (s, 2H), 4.12 (qd, *J* = 6.3, 2.9 Hz, 1H), 4.42 (d, *J* = 2.9 Hz, 1H), 7.19–7.23 (m, 2H), 7.52–7.56 (m, 2H); ^13^C NMR (101 MHz, D_2_O) δ 16.72, 41.15, 58.61, 61.43, 120.64, 131.08, 131.73, 133.54, 165.83, 174.42; HRMS: (ESI−), *m*/*z* calc. for C_12_H_12_O_5_N_2_BrS [M–H]^−^ 374.96558, found 374.96564.

#### 4.9.17 Potassium (2*S*,3*S*)-3-(2-Methoxy-2-phenylacetamido)-2-methyl-4-oxoazetidine-1-sulfonate (**15**)

Potassium (2*S*,3*S*)-3-(2-methoxy-2-phenylacetamido)-2-methyl-4-oxoazetidine-1-sulfonate was prepared following GP3 from 2-methoxy-2-phenylacetic acid (199 mg, 1.2 mmol) and purified following GP1. That afforded the desired product **15** as a white solid (68 mg, 18.5%). ^1^H NMR (400 MHz, DMSO-*d*_6_) δ 1.32 (d, *J* = 6.2 Hz, 3H), 3.26 (d, *J* = 1.7 Hz, 3H), 3.73 (qd, *J* = 6.2, 2.7 Hz, 1H), 4.31 (dd, *J* = 8.4, 2.7 Hz, 1H), 4.67 (s, 1H), 7.27–7.42 (m, 5H), 8.93 (d, *J* = 8.6 Hz, 1H); ^13^C NMR (101 MHz, DMSO-*d*_6_) δ 17.98, 56.60, 56.66, 60.19, 82.89, 127.11, 128.09, 128.22, 137.68, 162.62, 170.05; HRMS: (ESI−), *m*/*z* calc. for C_13_H_15_O_6_N_2_S [M–H]^−^ 327.06563, found 327.06557.

#### 4.9.18 Potassium (2*S*,3*S*)-3-(2-(3,4-Dimethoxyphenyl)­acetamido)-2-methyl-4-oxoazetidine-1-sulfonate (**16**)

Potassium (2*S*,3*S*)-3-(2-(3,4-dimethoxyphenyl)­acetamido)-2-methyl-4-oxoazetidine-1-sulfonate was prepared following GP3 from 2-(3,4-dimethoxyphenyl)­acetic acid (235 mg, 1.2 mmol) and purified following GP1. That afforded the desired product **16** as a white solid (85 mg, 21.5%). ^1^H NMR (400 MHz, D_2_O) δ 1.46 (d, *J* = 6.2 Hz, 3H), 3.51 (s, 2H), 3.77 (s, 3H), 3.79 (s, 3H), 4.11 (qd, *J* = 6.2, 2.8 Hz, 1H), 4.35 (d, *J* = 2.8 Hz, 1H), 6.80 (dd, *J* = 8.2, 2.0 Hz, 1H), 6.85–6.90 (m, 2H); ^13^C NMR (101 MHz, D_2_O) δ 16.78, 41.36, 55.52, 58.47, 61.51, 111.79, 112.57, 121.87, 127.34, 147.25, 148.07, 165.90, 174.82; HRMS: (ESI−), *m*/*z* calc. for C_14_H_17_O_7_N_2_S [M–H]^−^ 357.07619, found 357.07619.

#### 4.9.19 Potassium (2*S*,3*S*)-2-Methyl-4-oxo-3-(2-(thiophen-2-yl)­acetamido)­azetidine-1-sulfonate (**17**)

Potassium (2*S*,3*S*)-2-methyl-4-oxo-3-(2-(thiophen-2-yl)­acetamido)­azetidine-1-sulfonate was prepared following GP3 from 2-(thiophen-2-yl)­acetic acid (171 mg, 1.2 mmol) and purified following GP1. That afforded the desired product **17** as a white solid (11 mg, 3.2%). ^1^H NMR (400 MHz, D_2_O) δ 1.49 (d, *J* = 6.2 Hz, 3H), 3.87 (s, 2H), 4.14 (dd, *J* = 6.2, 2.9 Hz, 1H), 4.43 (d, *J* = 2.8 Hz, 1H), 6.99–7.02 (m, 1H), 7.02–7.05 (m, 1H), 7.37 (dd, *J* = 5.1, 1.3 Hz, 1H); ^13^C NMR (101 MHz, D_2_O) δ 16.73, 35.87, 58.56, 61.43, 125.80, 127.37, 127.46, 135.52, 165.81, 173.77; HRMS: (ESI−), *m*/*z* calc. for C_10_H_11_O_5_N_2_S_2_ [M–H]^−^ 303.01149, found 303.01159.

#### 4.9.20 Potassium (2*S*,3*S*)-2-Methyl-4-oxo-3-(2-(pyridin-4-yl)­acetamido)­azetidine-1-sulfonate (**18**)

Potassium (2*S*,3*S*)-2-methyl-4-oxo-3-(2-(pyridin-4-yl)­acetamido)­azetidine-1-sulfonate was prepared following GP3 from 2-(pyridin-4-yl)­acetic acid (208 mg, 1.2 mmol) and purified following GP1. That afforded the desired product **18** as a light-yellow solid (16 mg, 4.7%). ^1^H NMR (400 MHz, D_2_O) δ 1.50 (d, *J* = 6.3 Hz, 3H), 4.03 (s, 2H), 4.18 (qd, *J* = 6.2, 2.8 Hz, 1H), 4.44 (d, *J* = 2.8 Hz, 1H), 7.96–8.02 (m, 2H), 8.68–8.75 (m, 2H); ^13^C NMR (101 MHz, D_2_O) δ 16.74, 41.35, 58.35, 61.53, 128.20, 140.77, 156.07, 165.60, 170.65; HRMS: (ESI−), *m*/*z* calc. for C_11_H_12_O_5_N_3_S [M–H]^−^ 298.05031, found 298.05060.

#### 4.9.21 Potassium (2*S*,3*S*)-3-(2-((*tert*-Butoxycarbonyl)­amino)-2-phenylacetamido)-2-methyl-4-oxoazetidine-1-sulfonate (**19**)

Potassium (2*S*,3*S*)-3-(2-((*tert*-butoxycarbonyl)­amino)-2-phenylacetamido)-2-methyl-4-oxoazetidine-1-sulfonate was prepared following GP3 from 2-((*tert*-butoxycarbonyl)­amino)-2-phenylacetic acid (302 mg, 1.2 mmol) and purified following GP1. That afforded the desired product **19** as a white solid (89 mg, 19.7%). ^1^H NMR (400 MHz, D_2_O) δ 1.34 (s, 9H), 1.40 (d, *J* = 6.2 Hz, 3H), 4.09–3.98 (m, 1H), 4.37 (d, *J* = 2.8 Hz, 1H), 5.05 (s, 1H), 7.30–7.38 (m, 5H); HRMS: (ESI−), *m*/*z* calc. for C_17_H_22_O_7_N_3_S [M–H]^−^ 412.11839, found 412.11781.

#### 4.9.22 Potassium (2*S*,3*S*)-3-(2-Amino-2-phenylacetamido)-2-methyl-4-oxoazetidine-1-sulfonate (**20**)

Compound **19** (80 mg, 0.18 mmol) was suspended in dichloromethane (2 mL) and trifluoroacetic acid (100 μL, 1.3 mmol, 7.3 equiv) was added at room temperature. After 16 h, solvent was removed *in vacuo*, and product was purified following GP1, which afforded **20** as white solid (17 mg, 26.9%). Purity: 85.3%, ^1^H NMR (400 MHz, D_2_O) δ 1.34 (d, *J* = 6.2 Hz, 2H), 1.38 (d, *J* = 6.2 Hz, 3H), 4.00–4.11 (m, 2H), 4.23 (d, *J* = 2.9 Hz, 1H), 4.31 (d, *J* = 2.9 Hz, 1H), 5.09 (d, *J* = 4.9 Hz, 2H), 7.35–7.47 (m, 10H); ^13^C NMR (101 MHz, D_2_O) δ 16.68, 56.44, 57.93, 58.24, 61.38, 128.12, 129.74, 130.51, 131.43, 165.27, 165.42, 168.78; HRMS: (ESI−), *m*/*z* calc. for C_12_H_14_O_5_N_3_S [M–H]^−^ 312.06596, found 312.06561.

#### 4.9.23 Potassium (2*S*,3*S*)-2-Methyl-4-oxo-3-(2-phenylacetamido)­azetidine-1-sulfonate (**21**)

Potassium (2*S*,3*S*)-2-methyl-4-oxo-3-(2-phenylacetamido)­azetidine-1-sulfonate was prepared following GP3 from 2-phenylacetic acid (163 mg, 1.2 mmol) and purified following GP1. That afforded the desired product **21** as a white solid (166 mg, 49.3%). ^1^H NMR (400 MHz, D_2_O) δ 1.39 (d, *J* = 6.2 Hz, 3H), 3.54 (s, 2H), 4.04 (qd, *J* = 6.2, 2.8 Hz, 1H), 4.27 (d, *J* = 2.6 Hz, 1H), 7.19–7.37 (m, 5H); ^13^C NMR (101 MHz, D_2_O) δ 16.79, 41.87, 58.50, 61.51, 127.43, 128.98, 129.27, 134.48, 165.98, 174.86; HRMS: (ESI−), [M–H]^−^ calc. for C_12_H_13_O_5_N_2_S 297.05397, found 297.05450.

#### 4.9.24 (2*S*,3*S*)-3-((*Z*)-2-(2-Aminothiazol-4-yl)-2-((2-(*tert*-butoxy)-2-oxoethoxy)­imino)­acetamido)-2-methyl-4-oxoazetidine-1-sulfonic acid, DIPEA Salt (**22**)

(*Z*)-2-(2-aminothiazol-4-yl)-2-((2-(*tert*-butoxy)-2-oxoethoxy)­imino)­acetic acid (301 mg, 1.0 mmol) was disolved in DMF (5 mL) and *N*,*N*′-dicyclohexylcarbodiimide (227 mg, 1.1 mmol, 1.1 equiv) and *N*-hydroxysuccinimide (127 mg, 1.1 mmol, 1.1 equiv) were added. After 2 h water was added and white precipitate was filtered off, dried, and dissolved in dry DMF (7 mL). Next, (2*S*,3*S*)-3-amino-2-methyl-4-oxoazetidine-1-sulfonic acid (180 mg, 1.0 mmol, 1.0 equiv) and DIPEA (173 μL, 1.0 mmol, 1.0 equiv) were added and the reaction was stirred for 16 h at 70 °C. The solvent was evaporated *in vacuo*, and the product was purified following GP1, which afforded desired product **22** as white solid (30 mg, 5.1%). ^1^H NMR (400 MHz, D_2_O) δ 1.22–1.41 (m, 15H), 1.47 (s, 9H), 1.57 (d, *J* = 6.2 Hz, 3H), 3.17 (q, *J* = 7.5 Hz, 2H), 3.69 (p, *J* = 6.7 Hz, 2H), 4.27 (qd, *J* = 6.2, 2.8 Hz, 1H), 4.65 (d, *J* = 2.8 Hz, 1H), 4.69 (s, 2H), 7.01 (s, 1H); ^13^C NMR (101 MHz, D_2_O) δ 12.11, 16.20, 16.82, 17.68, 27.21, 42.52, 54.32, 58.49, 61.11, 71.66, 84.29, 113.89, 138.88, 148.27, 163.43, 164.66, 170.76, 170.83; HRMS: (ESI-), [M–H]^−^ calc. for C_15_H_21_O_8_N_5_S_2_ 462.07478, found 462.07635.

#### 4.9.25 Potassium (2*S*,3*S*)-3-(2-(2,5-Dimethylthiazol-4-yl)­acetamido)-2-methyl-4-oxoazetidine-1-sulfonate (**23**)

Potassium (2*S*,3*S*)-3-(2-(2,5-dimethylthiazol-4-yl)­acetamido)-2-methyl-4-oxoazetidine-1-sulfonate was prepared following GP3 from 2-(2,5-dimethylthiazol-4-yl)­acetic acid (205 mg, 1.2 mmol) and purified following GP1. That afforded the desired product **23** as white oil (18 mg, 4.9%). ^1^H NMR (400 MHz, D_2_O) δ 1.49 (d, *J* = 6.2 Hz, 3H), 2.33 (s, 3H), 2.64 (s, 3H), 3.73 (s, 2H), 4.17 (qd, *J* = 6.2, 2.9 Hz, 1H), 4.40 (d, *J* = 2.9 Hz, 1H); ^13^C NMR (101 MHz, D_2_O) δ 10.29, 16.75, 16.94, 33.92, 58.42, 61.51, 131.59, 140.59, 165.88, 166.39, 172.18; HRMS: (ESI-), [M–H]^−^ calc. for C_11_H_14_O_5_N_3_S_2_ 332.03804, found 332.03760.

#### 4.9.26 (2*S*,3*S*)-3-(*Z*)-2-(5-Amino-1,2,4-thiadiazol-3-yl)-2-(ethoxyimino)­acetamido-2-methyl-4-oxoazetidine-1-sulfonic acid, DIPEA salt (**24**)

(2*S*,3*S*)-3-(*Z*)-2-(5-amino-1,2,4-thiadiazol-3-yl)-2-(ethoxyimino)­acetamido-2-methyl-4-oxoazetidine-1-sulfonic acid, DIPEA salt was prepared following GP3 from (*Z*)-2-(5-amino-1,2,4-thiadiazol-3-yl)-2-(ethoxyimino)­acetic acid (259 mg, 1.2 mmol), with a slight modification of heating during the carboxylic acid activation at 50 °C and purified following GP1. That afforded the desired product **24** as white oil (28 mg, 7.4%). ^1^H NMR (400 MHz, D_2_O) δ 1.14–1.25 (m, 18H), 1.48 (d, *J* = 6.2 Hz, 3H), 1.77 (q, *J* = 3.3 Hz, 2H), 3.07–3.14 (m, 2H), 3.56–3.67 (m, 5H), 4.13 (qd, *J* = 6.2, 2.8 Hz, 1H), 4.22 (q, *J* = 7.0 Hz, 2H), 4.59 (d, *J* = 2.8 Hz, 1H); ^13^C NMR (101 MHz, D_2_O) δ 12.09, 13.70, 16.19, 16.79, 17.66, 24.96, 42.51, 54.32, 58.72, 60.96, 67.80, 72.25, 127.22, 146.22, 161.07, 163.60, 164.61; HRMS: (ESI−), [M–H]^−^ calc. for C_10_H_13_O_6_N_6_S_2_ 377.03325, found 377.03488.

#### 4.9.27 Potassium (2*S*,3*S*)-2-Methyl-3-(2-(2-methylthiazol-4-yl)­acetamido)-4-oxoazetidine-1-sulfonate (**25**)

Potassium (2*S*,3*S*)-2-methyl-3-(2-(2-methylthiazol-4-yl)­acetamido)-4-oxoazetidine-1-sulfonate was prepared following GP3 from 2-(2-methylthiazol-4-yl)­acetic acid (259 mg, 1.2 mmol) and purified following GP1. That afforded the desired product **25** as a white solid (35 mg, 9.8%). ^1^H NMR (400 MHz, D_2_O) δ 1.41 (d, *J* = 6.2 Hz, 3H), 2.58 (s, 3H), 3.67 (s, 2H), 4.08 (qd, *J* = 6.2, 2.9 Hz, 1H), 4.35 (d, *J* = 2.8 Hz, 1H), 7.12 (s, 1H); ^13^C NMR (101 MHz, D_2_O) δ 16.74, 17.58, 36.86, 58.53, 61.47, 117.51, 146.88, 165.92, 169.18, 172.89; HRMS: (ESI−), [M–H]^−^ calc. for C_10_H_12_O_5_N_3_S_2_ 318.02239, found 318.02190.

#### 4.9.28 Potassium (2*S*,3*S*)-3-((*Z*)-2-(2-Aminothiazol-4-yl)-2-((2-methoxy-2-oxoethoxy)­imino)­acetamido)-2-methyl-4-oxoazetidine-1-sulfonate (**26**)

Potassium (2*S*,3*S*)-3-((*Z*)-2-(2-aminothiazol-4-yl)-2-((2-methoxy-2-oxoethoxy)­imino)­acetamido)-2-methyl-4-oxoazetidine-1-sulfonate was prepared following GP3 from (*Z*)-2-(2-aminothiazol-4-yl)-2-((2-methoxy-2-oxoethoxy)­imino)­acetic acid (311 mg, 1.2 mmol), with a slight modification of heating at 50 °C during the carboxylic acid activation and was purified following GP1. That afforded the desired product **26** as a yellow solid (42 mg, 9.1%). ^1^H NMR (400 MHz, D_2_O) δ 1.46 (d, *J* = 6.2 Hz, 3H), 3.69 (s, 3H), 4.18 (qd, *J* = 6.2, 2.8 Hz, 1H), 4.54 (d, *J* = 2.8 Hz, 1H), 4.80 (s, 2H), 7.07 (s, 1H); ^13^C NMR (101 MHz, D_2_O) δ 16.80, 52.72, 58.31, 61.20, 71.72, 112.41, 130.55, 143.87, 160.86, 164.58, 170.59, 171.73; HRMS: (ESI−), [M–H]^−^ calc. for C_12_H_14_O_8_N_5_S_2_ 420.02893, found 420.02856.

#### 4.9.29 (2*S*,3*S*)-3-((*Z*)-2-(2-Aminothiazol-4-yl)­pent-2-enamido)-2-methyl-4-oxoazetidine-1-sulfonic acid DIPEA salt (**27**)

To a stirred solution of (*Z*)-2-(2-aminothiazol-4-yl)-2-((2-methoxy-2-oxoethoxy)­imino)­acetic acid (298 mg, 1.0 mmol) in THF, DIPEA (346 μL, 2.0 mmol) and ethyl chloroformate were added (114 μL, 1.2 mmol) and stirred for 1 h at 0 °C. Subsequently, (2*S*,3*S*)-3-amino-2-methyl-4-oxoazetidine-1-sulfonic acid (180 mg, 1.0 mmol) and DIPEA (173 μL, 1.0 mmol) were added. The reaction was stirred at 50 °C for 24 h. The solvent was removed under reduced pressure, and the product was purified following GP1. This afforded the desired product **27** as an off-white solid (67 mg, 11.4%). ^1^H NMR (400 MHz, D_2_O) δ 0.99 (t, *J* = 7.5 Hz, 3H), 1.22–1.31 (m, 8H), 1.42–1.46 (m, 9H), 1.54 (d, *J* = 6.2 Hz, 3H), 2.19 (p, *J* = 7.1 Hz, 2H), 3.12 (q, *J* = 7.4 Hz, 1H), 3.63 (p, *J* = 6.6 Hz, 1H), 4.23 (qd, *J* = 6.1, 5.5, 3.2 Hz, 1H), 4.56 (d, *J* = 2.8 Hz, 1H), 6.36 (t, *J* = 7.8 Hz, 1H), 6.77 (s, 1H); ^13^C NMR (101 MHz, D_2_O) δ 12.16, 13.17, 16.28, 17.01, 17.74, 22.72, 27.51, 42.52, 54.32, 58.41, 61.43, 83.48, 108.82, 129.84, 136.26, 145.81, 153.45, 160.64, 165.15, 170.39; HRMS: (ESI+), [M + H]^+^ calc. for C_17_H_25_O_7_N_4_S_2_ 461.11592, found 461.11595.

## Supplementary Material











## Notes

Figure S1 depicting interactions of synthetized compounds, Figure S2 with enzyme inhibition curves of PBP3 for compounds **6** and **11**, Table S1 with structures and scores for pharmacophore models, Tables S2−S4 with determined *in vitro* antibacterial activity (MIC values) for compounds **2**−**27** and Table S5 with statistical parameters for determined crystal structures regarding data collection, phasing and structure refinement statistics; It includes NMR spectra and other analytical data for compounds **2**−**27** (PDF)
Molecular string formulas (CSV)
Pharmacophoric Model 1 (PDB)
Pharmacophoric Model 2 (PDB)
Pharmacophoric Model 3 (PDB)

## Abbreviations Used

AMR: antimicrobial resistance
ATMO: 2-amino-4-thiazolyl methoxyimino
DIPEA: *N*,*N*-diisopropylethylamine
ESBL: extended spectrum β-lactamase
MBL: metallo-β-lactamase
MIC: minimum inhibitory concentration
MRSA: methicillin-resistant *Staphylococcus aureus*
PBP: penicillin-binding protein
SAR: structure–activity relationship
