# Supplementary material for: Structure–Activity Relationship and Crystallographic Study of New Monobactams
Source: J Med Chem. 2026 Feb 3;69(4):3887–901. doi: 10.1021/acs.jmedchem.5c02427 (PMC12951460; doi:10.1021/acs.jmedchem.5c02427)
Supplement: Supplementary file 1 [file jm5c02427_si_001.pdf]

SUPPORTING INFORMATION

Structure-activity relationship and crystallographic study of new monobactams

Vid Kavaš[1], Carlos Contreras-Martel[2], Stane Pajk[1], Damijan Knez[1], Alexandre Martins[2], Thomas A Gould[3], David I Roper[3], Irena Zdovc[4], Andréa Dessen[2], Martina Hrast Rambaher[1], Stanislav Gobec[1]*

[1] Department of Pharmaceutical Chemistry, Faculty of Pharmacy, University of Ljubljana, Aškerčeva cesta 7, 1000 Ljubljana, Slovenia.

[2] University Grenoble Alpes, CNRS, CEA, Institut de Biologie Structurale (IBS), 38044 Grenoble, France.

[3] School of Life Sciences, University of Warwick, Gibbet Hill Road, Coventry, CV4 7AL, UK.

[4] Institute of Microbiology and Parasitology, Veterinary Faculty, University of Ljubljana, Gerbičeva 60, 1000 Ljubljana, Slovenia.

*Corresponding Author

Tel: +386 14769585

Email: stanislav.gobec@ffa.uni-lj.si

Contents:

1. ^{1}H and ^{13}C NMR spectra, HPLC and HRMS analysis

2. Interactions of synthetized compounds
3. Enzyme inhibition curves and MIC determination against ESKAPE pathogens
4. Statistical parameters of crystal structures

^{1}H AND ^{13}C NMR SPECTRA, HPLC AND HRMS ANALYSIS

Potassium (2*S*,3*S*)-3-(6-aminonicotinamido)-2-methyl-4-oxoazetidine-1-sulfonate (**2**)

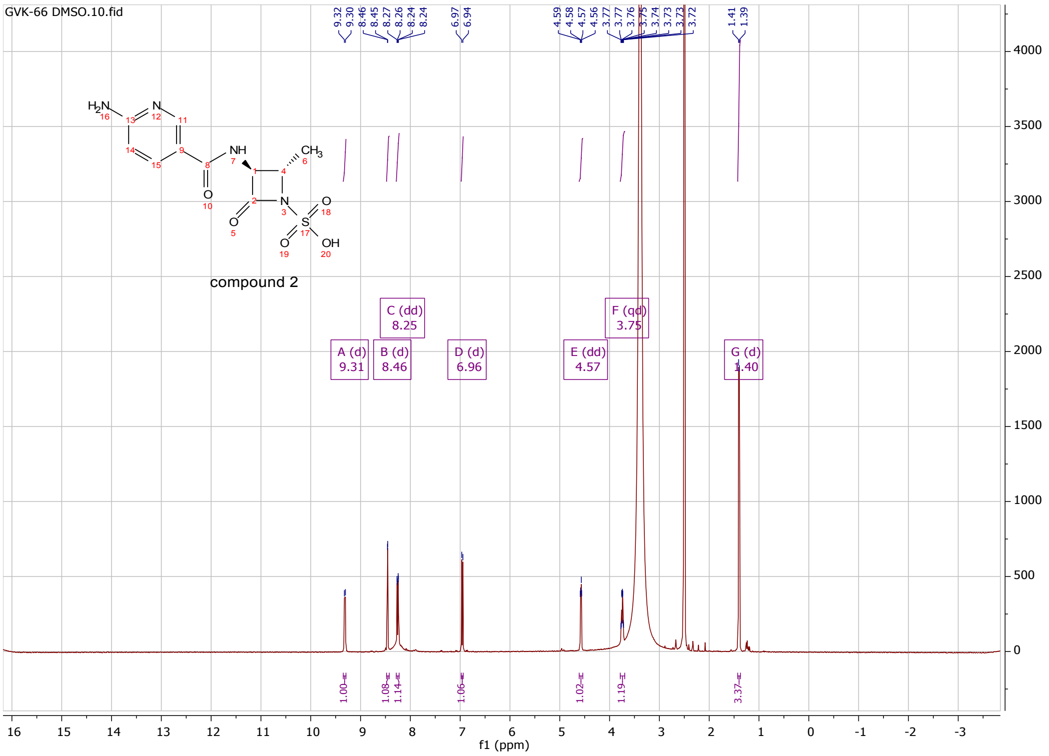

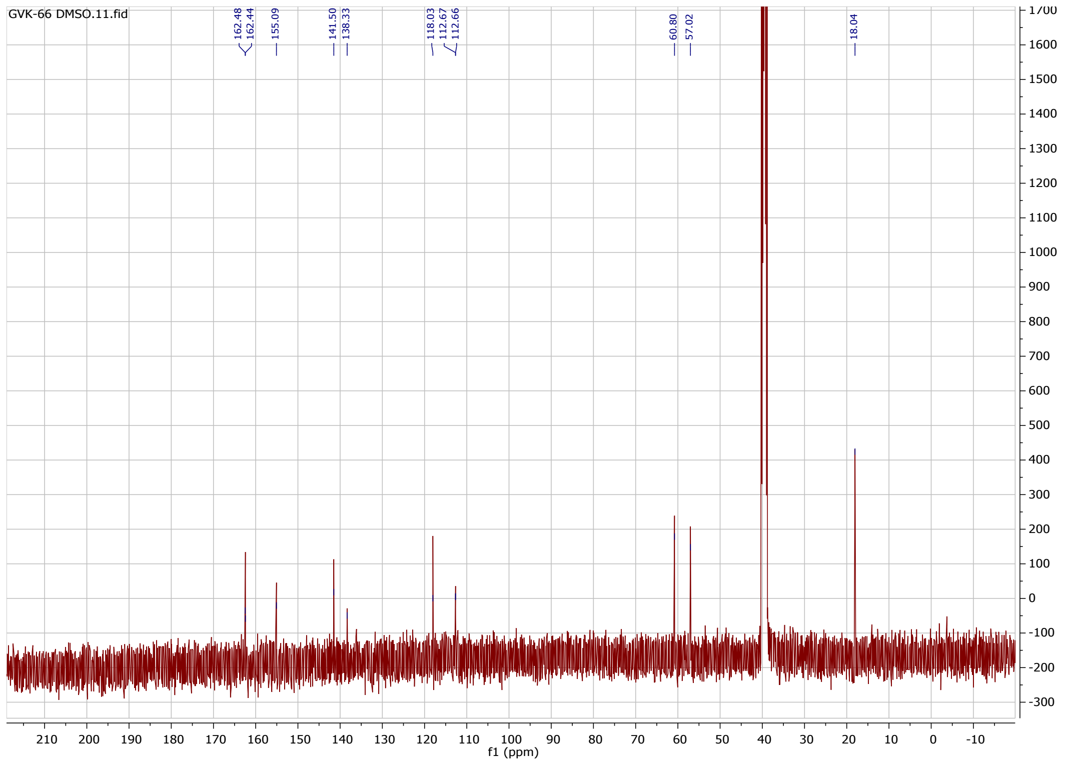

HRMS: (ESI–), *m/z* calc. for $C_{10}H_{11}O_5N_4S$ [M–H]$^-$ 299.04556, found 299.04510.

Potassium (2*S*,3*S*)-3-(2-bromo-3-nitrobenzamido)-2-methyl-4-oxoazetidine-1-sulfonate (**3**)

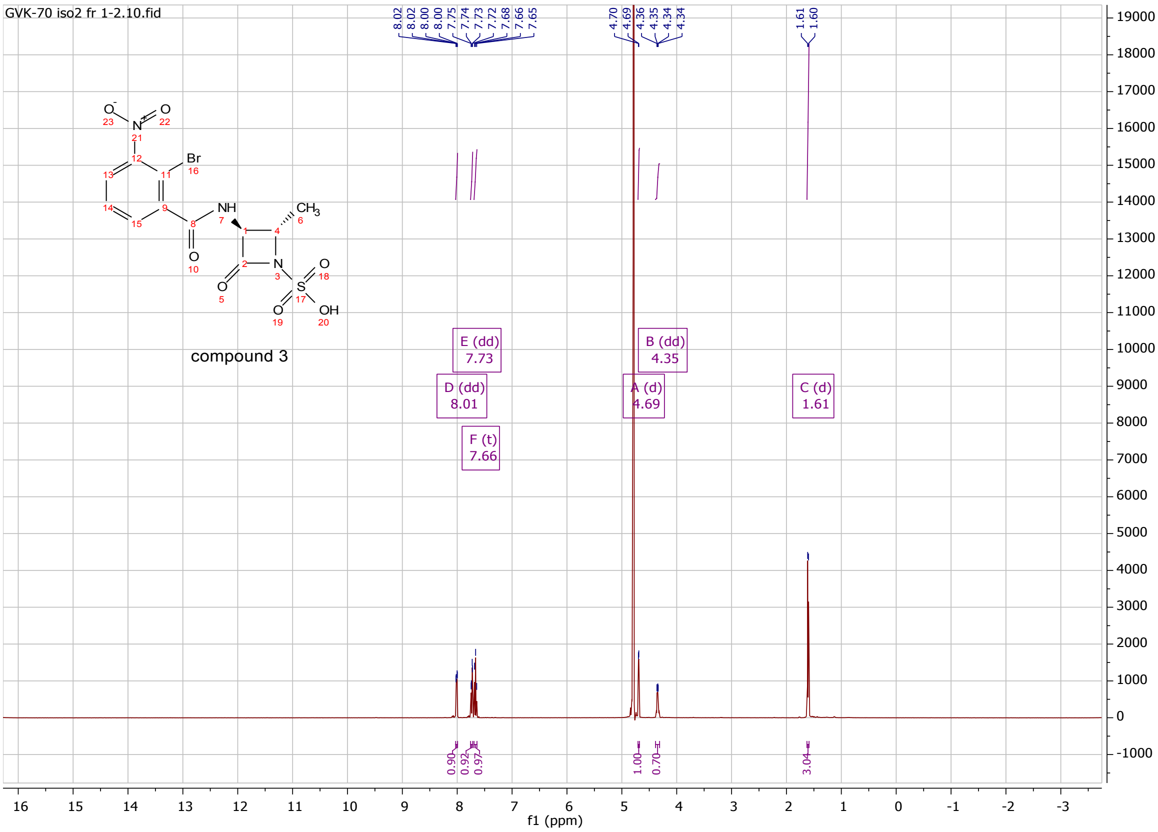

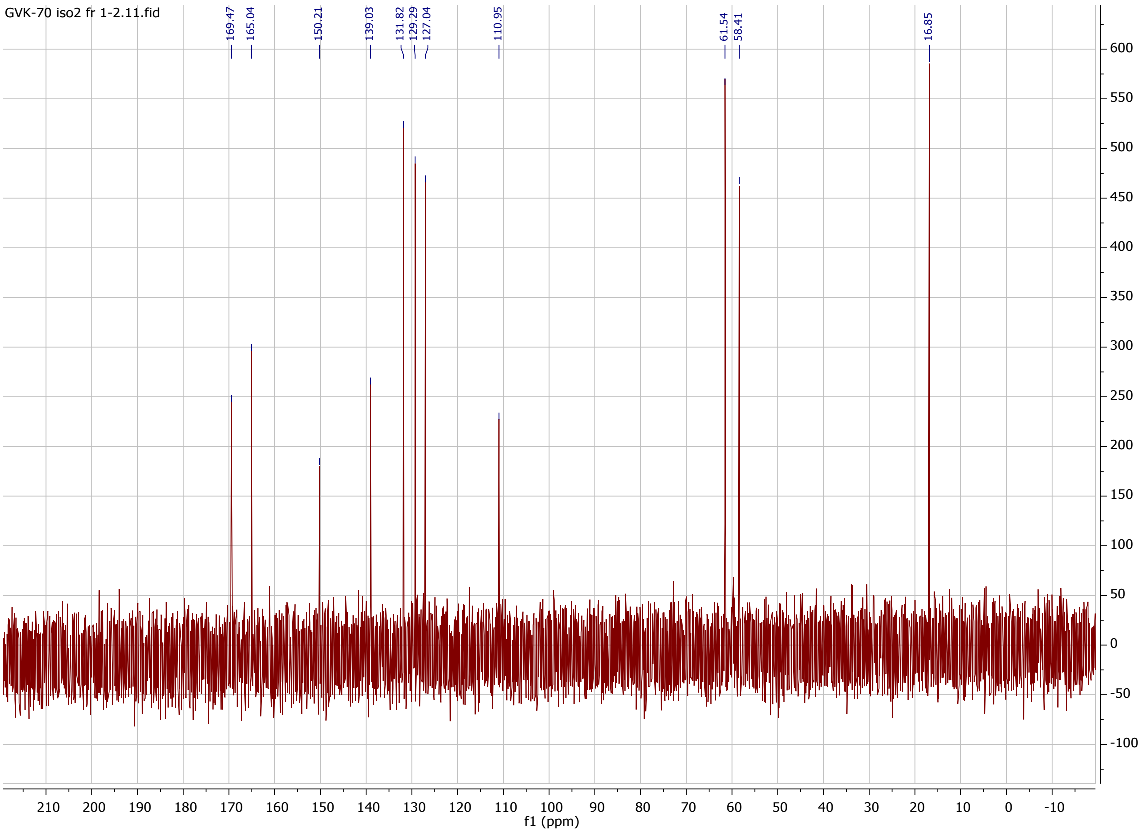

HRMS: (ESI–), *m/z* calc. for $C_{11}H_9O_7N_3SBr$ $[M–H]^-$ 405.93391, found 405.93480.

Potassium (2*S*,3*S*)-3-(4-azidobenzamido)-2-methyl-4-oxoazetidine-1-sulfonate (**4**)

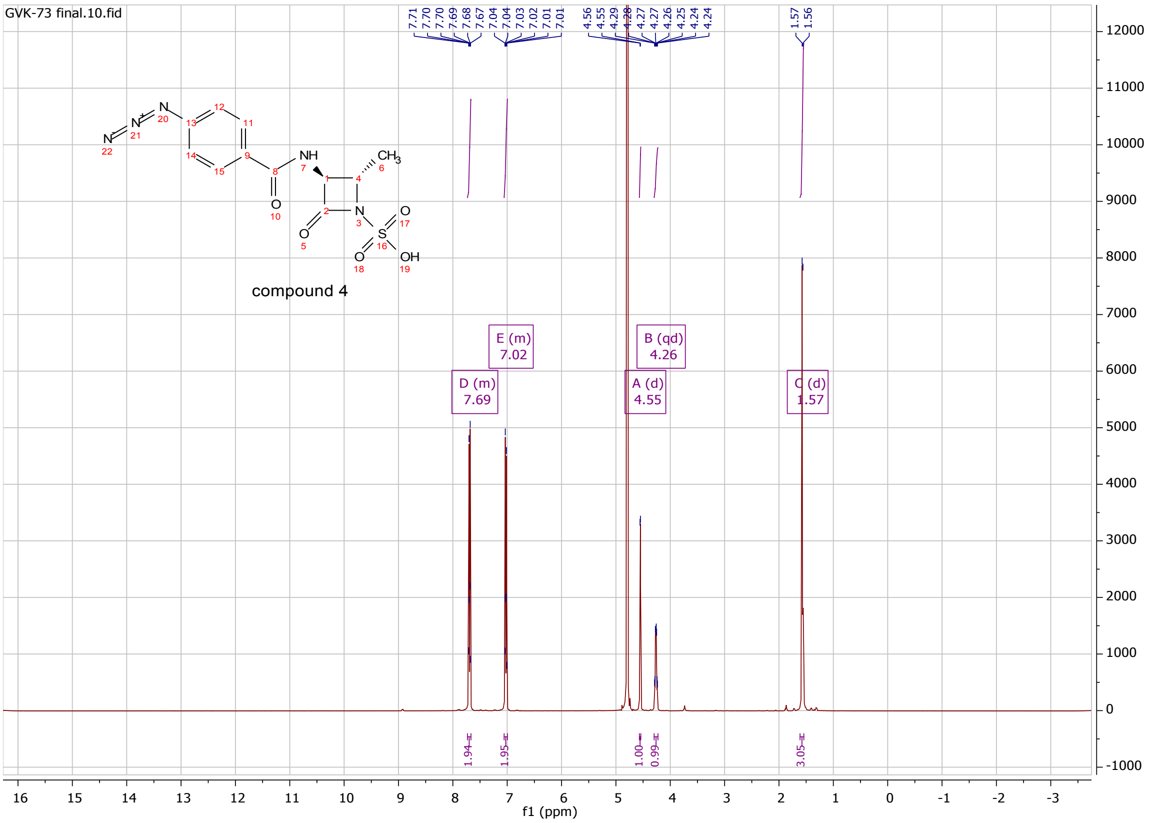

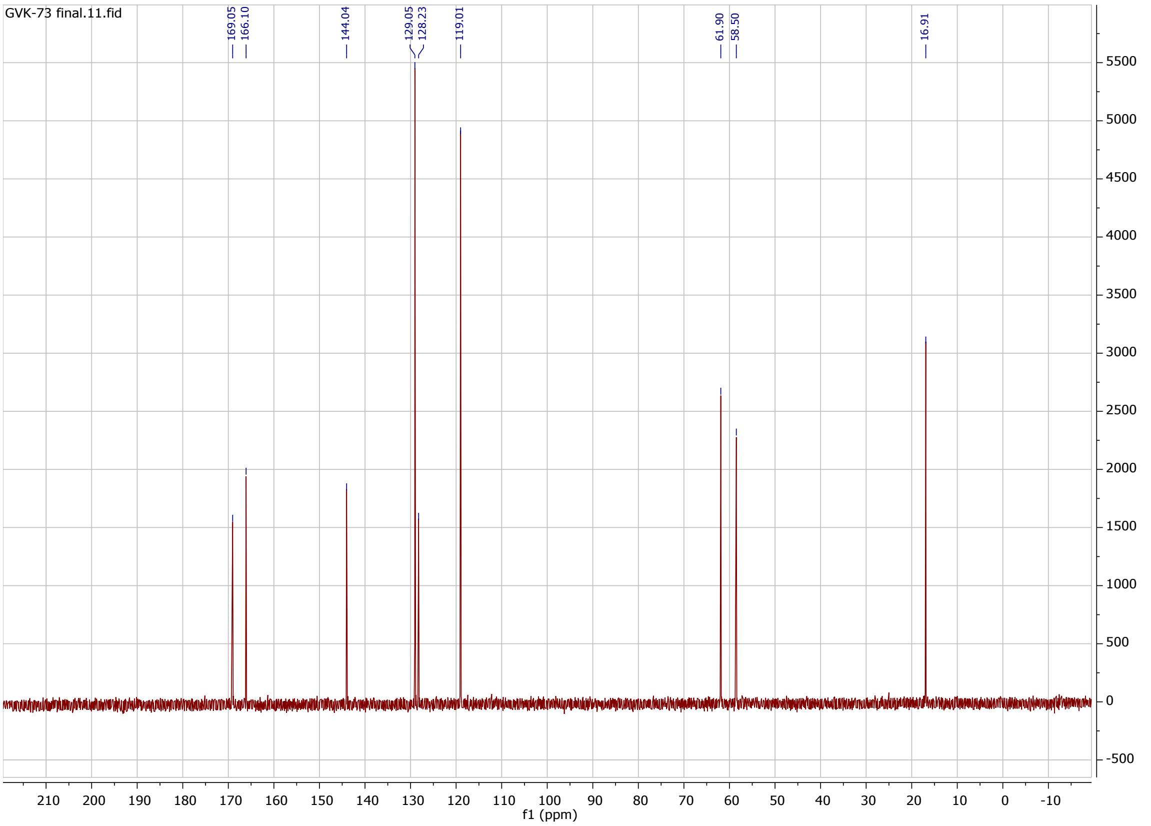

HRMS: (ESI–), *m/z* calc. for $C_{11}H_{10}O_5N_5S$ $[M–H]^-$ 324.04081, found 324.04051.

Sodium (2*S*,3*S*)-3-(6-hydroxynicotinamido)-2-methyl-4-oxoazetidine-1-sulfonate (**5**)

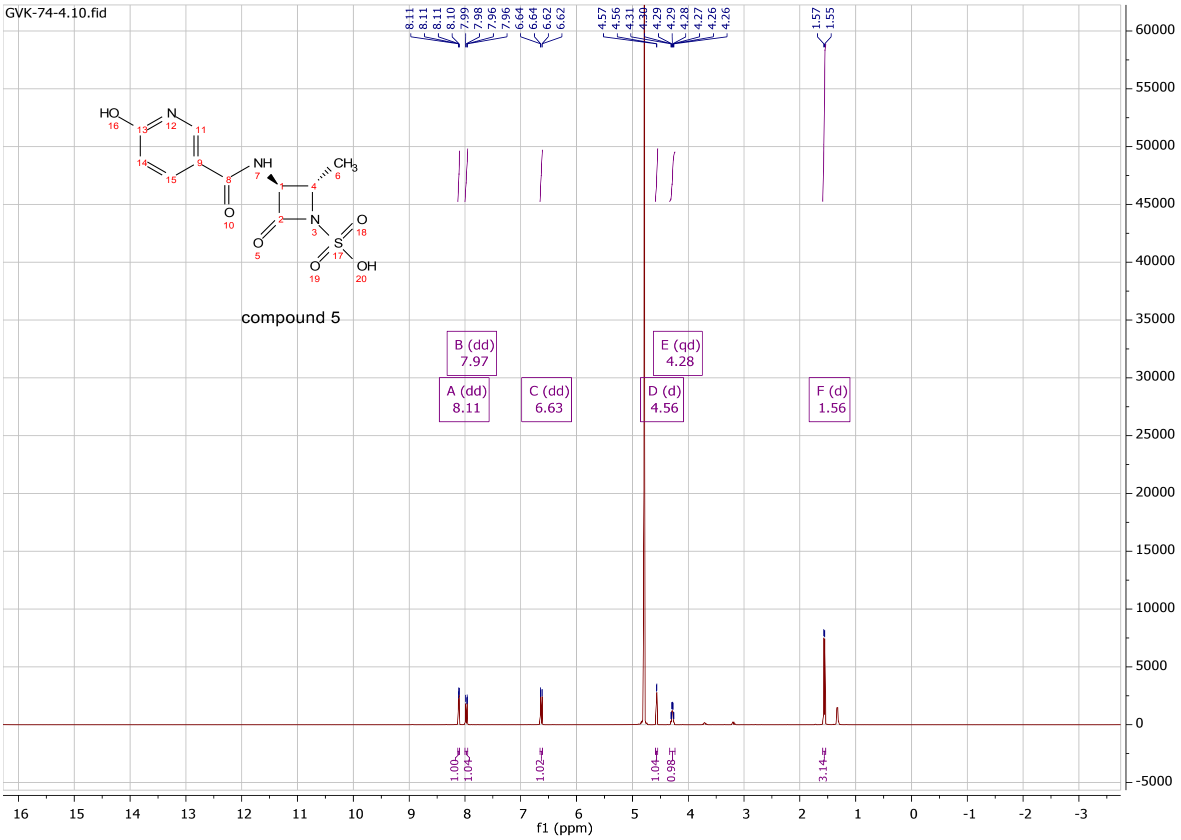

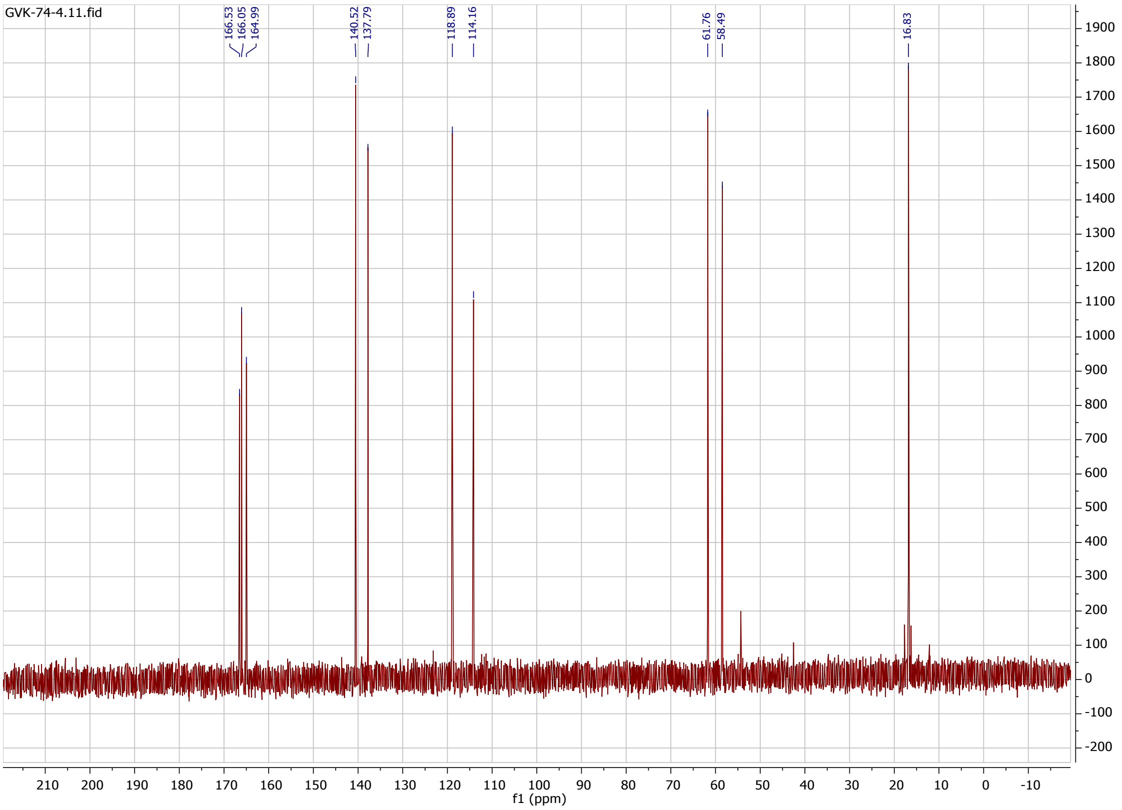

HRMS: (ESI–), *m/z* calc. for $C_{10}H_{10}O_6N_3S$ [M–H]$^-$ 300.02848, found 300.03000.

Potassium (2*S*,3*S*)-3-(2-(3-hydroxyphenyl)acetamido)-2-methyl-4-oxoazetidine-1-sulfonate (**6**)

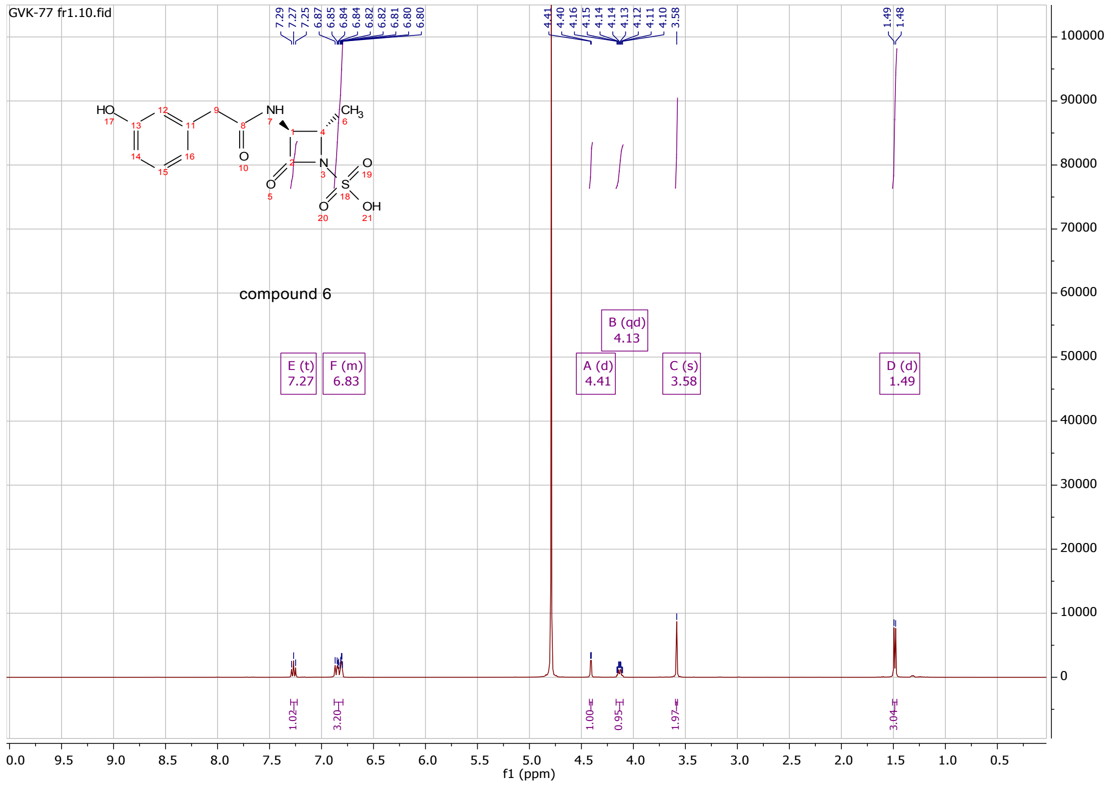

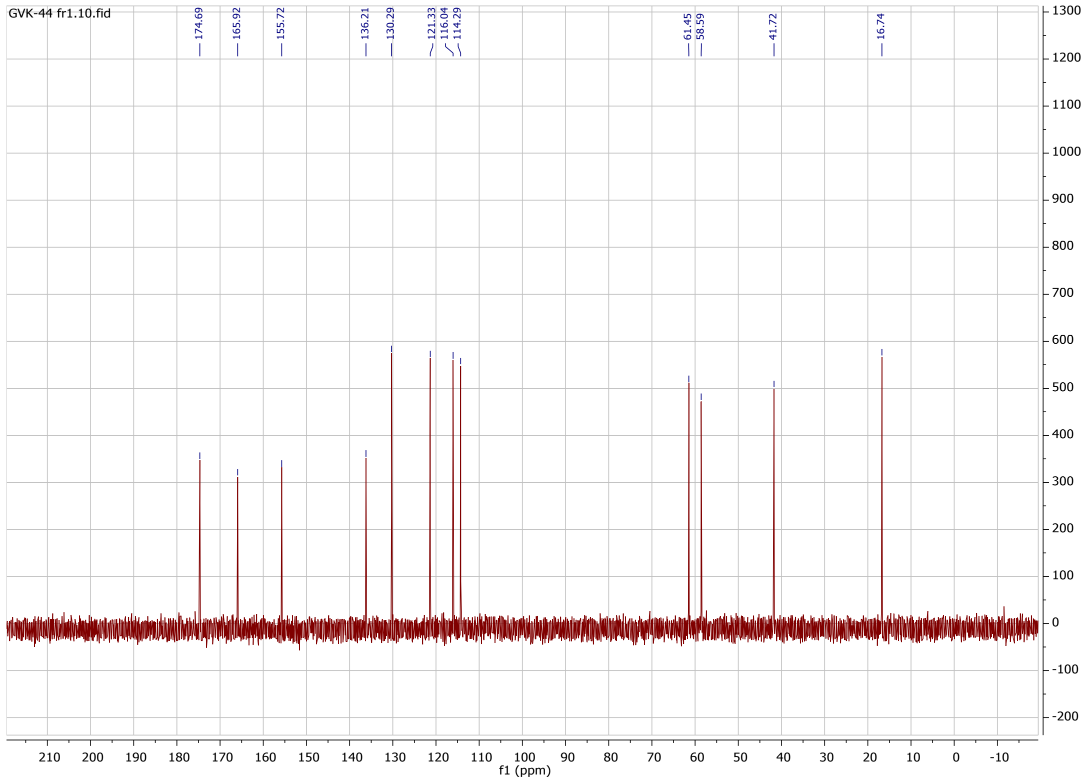

HRMS: (ESI–), *m/z* calc. for $C_{12}H_{13}O_6N_2S$ $[M–H]^-$ 313.04998, found 313.04968.

Potassium (2*S*,3*S*)-3-(4-amino-3-fluorobenzamido)-2-methyl-4-oxoazetidine-1-sulfonate (**7**)

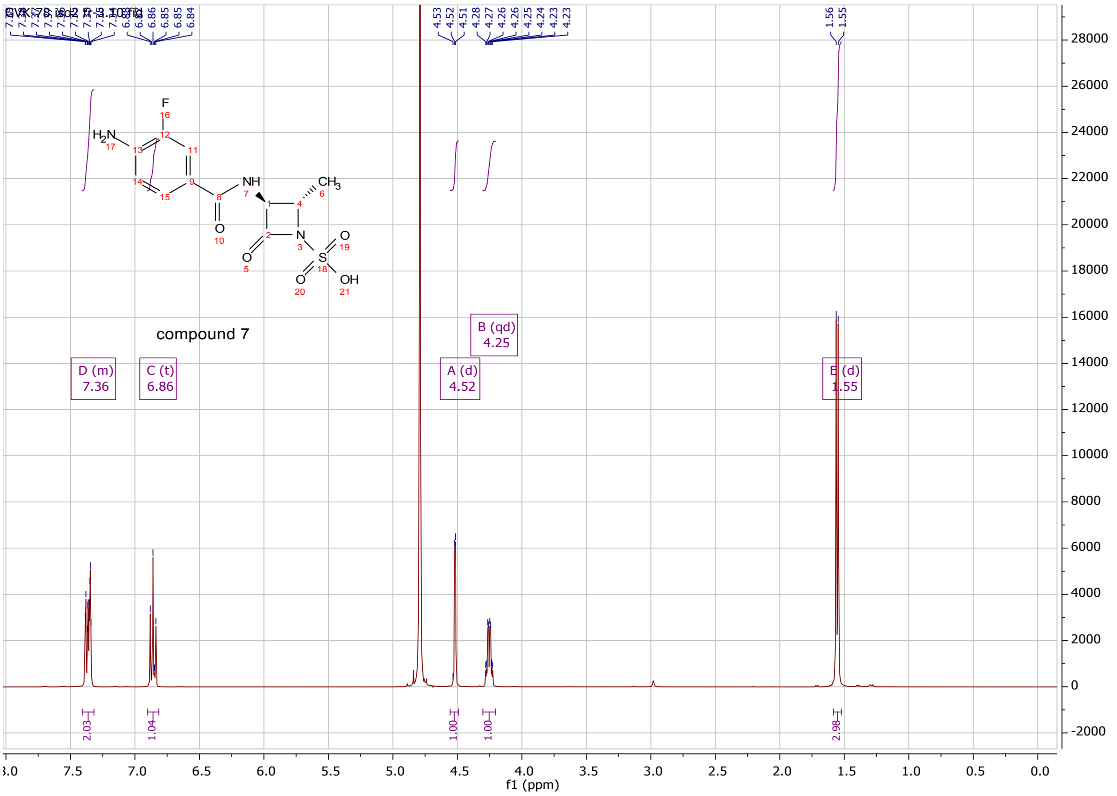

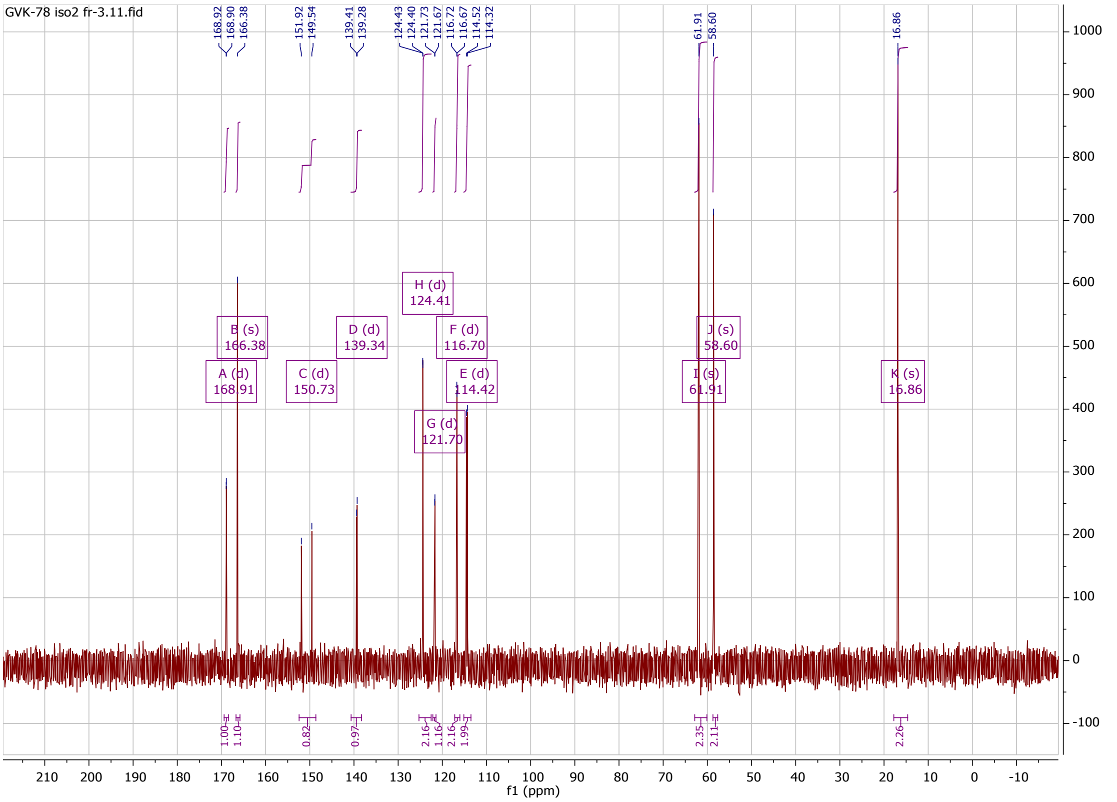

HRMS: (ESI–), *m/z* calc. for $C_{11}H_{11}O_5N_3SF$ $[M–H]^-$ 316.04089, found 316.04050.

(2*S*,3*S*)-3-(2-(2-aminothiazol-4-yl)acetamido)-2-methyl-4-oxoazetidine-1-sulfonic acid DIPEA salt (**8**)

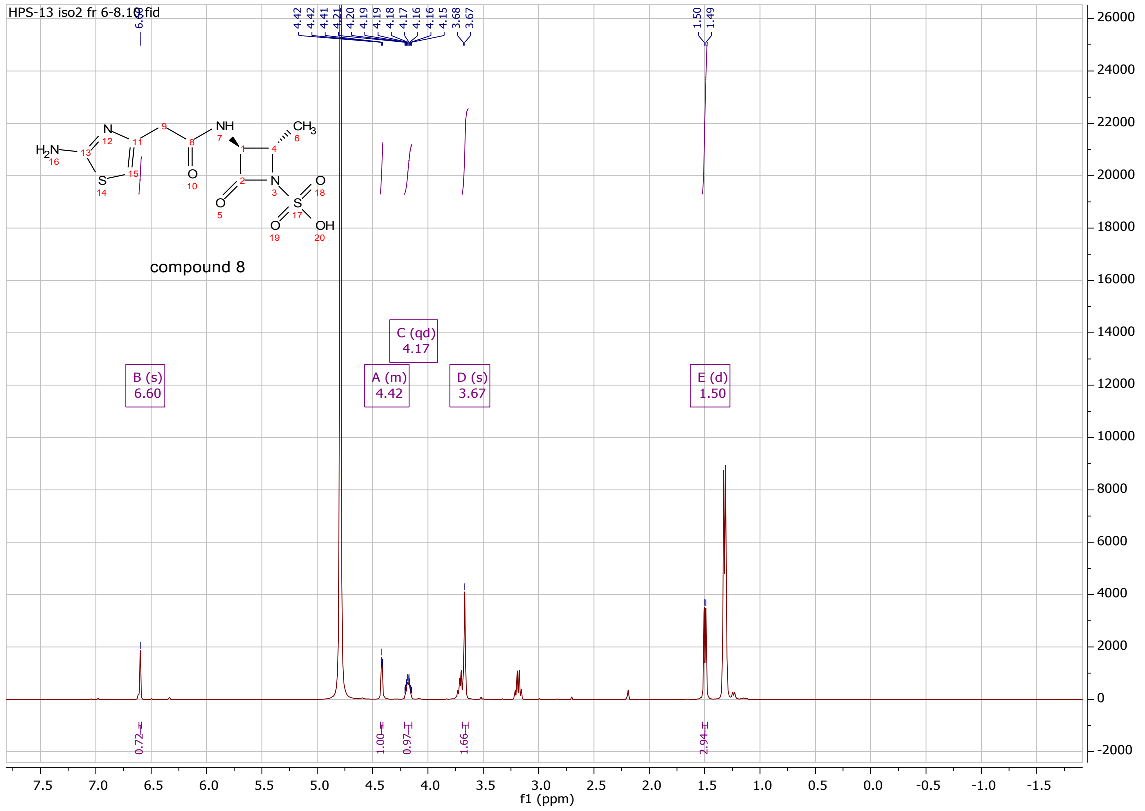

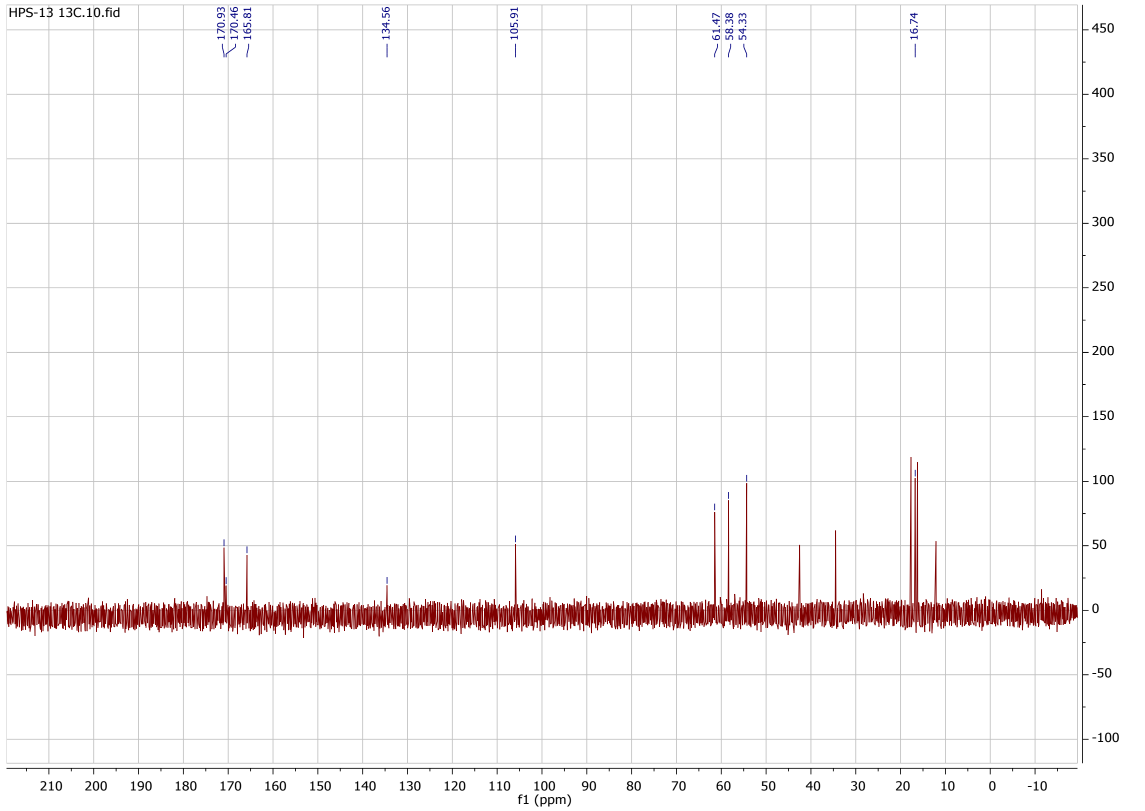

HRMS: (ESI–), *m/z* calc. for $C_9H_{11}O_5N_4S_2$ $[M–H]^-$ 319.01763, found 319.01794.

Potassium (2*S*,3*S*)-3-(2-(1*H*-indol-3-yl)acetamido)-2-methyl-4-oxoazetidine-1-sulfonate (**9**)

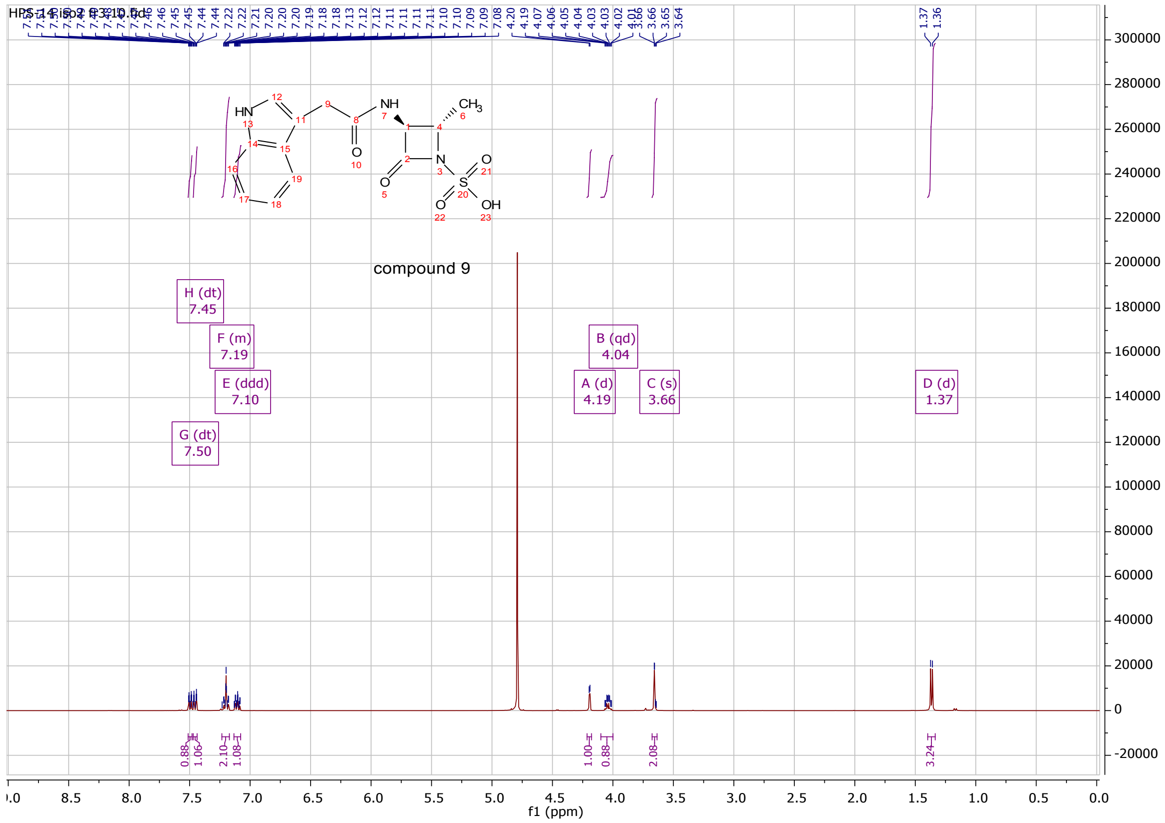

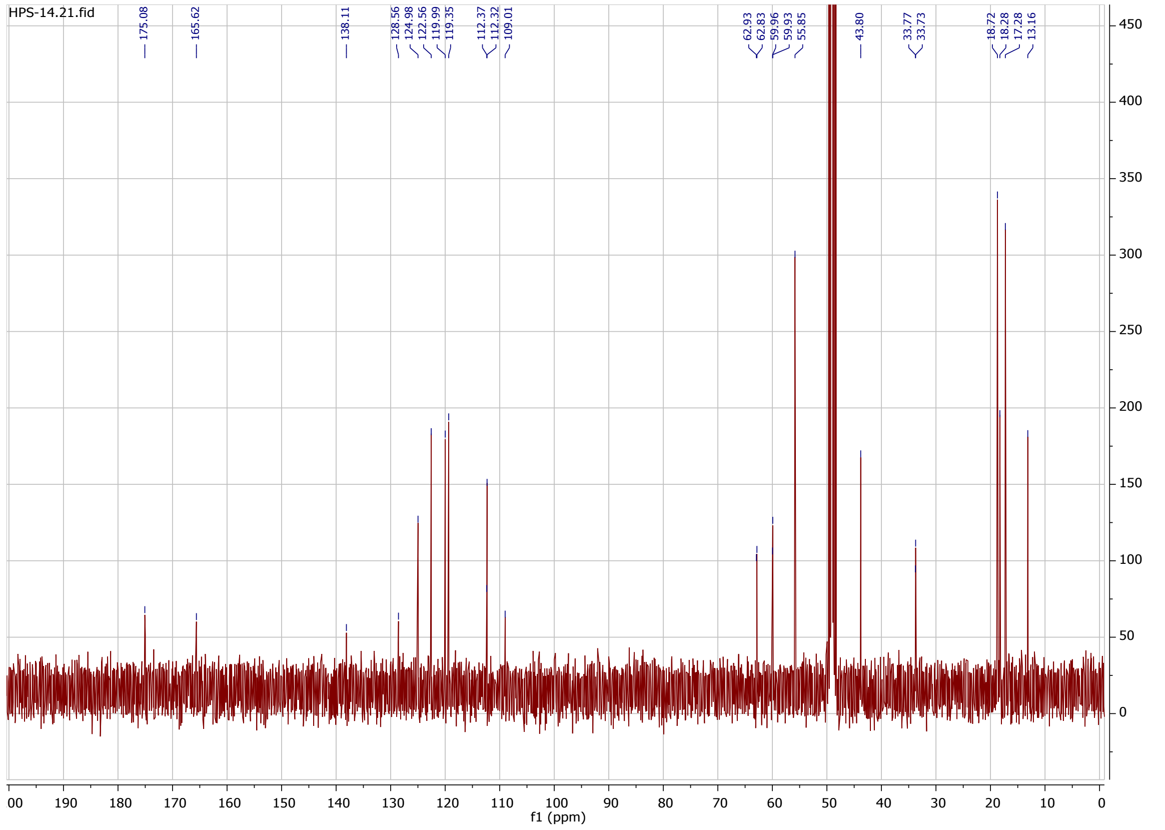

HRMS: (ESI–), *m/z* calc. for $C_{14}H_{14}O_5N_3S$ $[M–H]^-$ 336.06596, found 336.06633.

Potassium (2*S*,3*S*)-2-methyl-3-(2-(4-nitrophenyl)acetamido)-4-oxoazetidine-1-sulfonate (**10**)

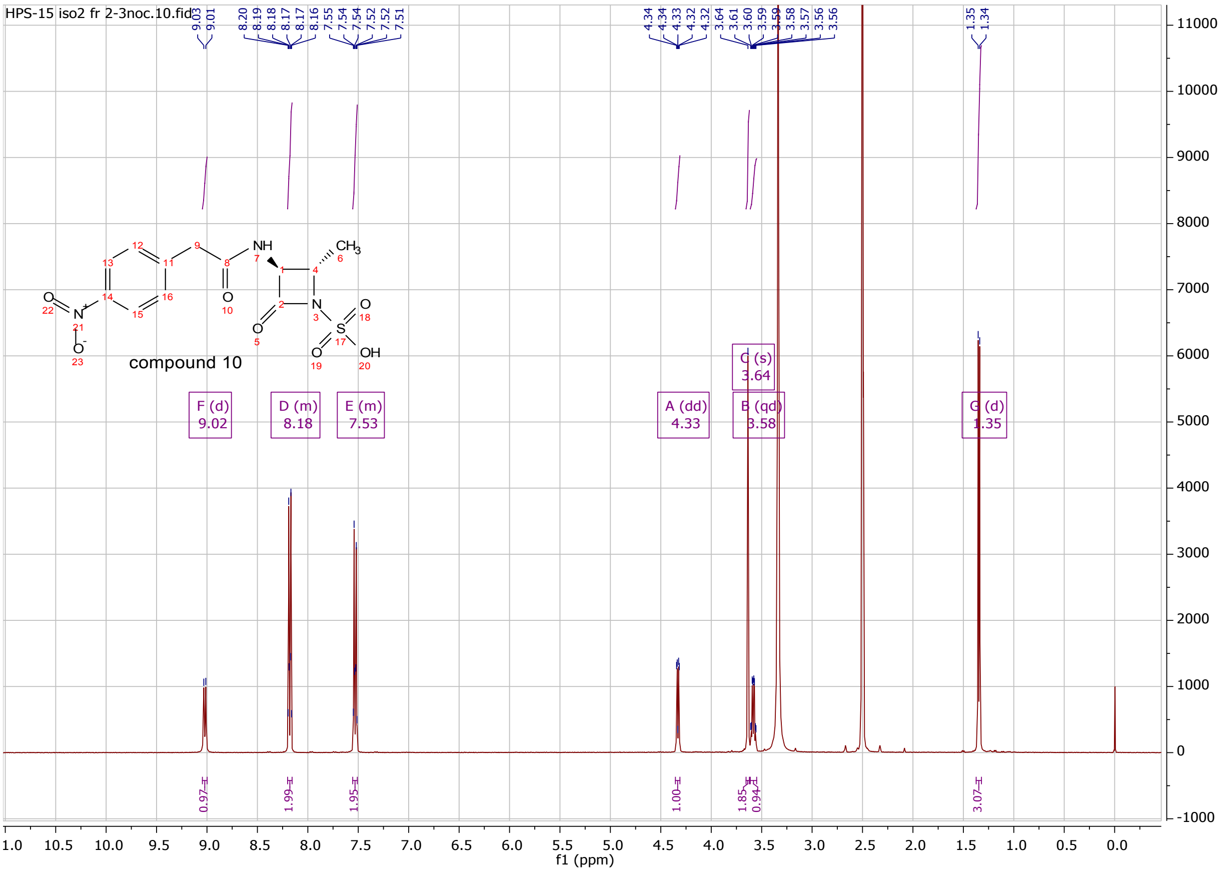

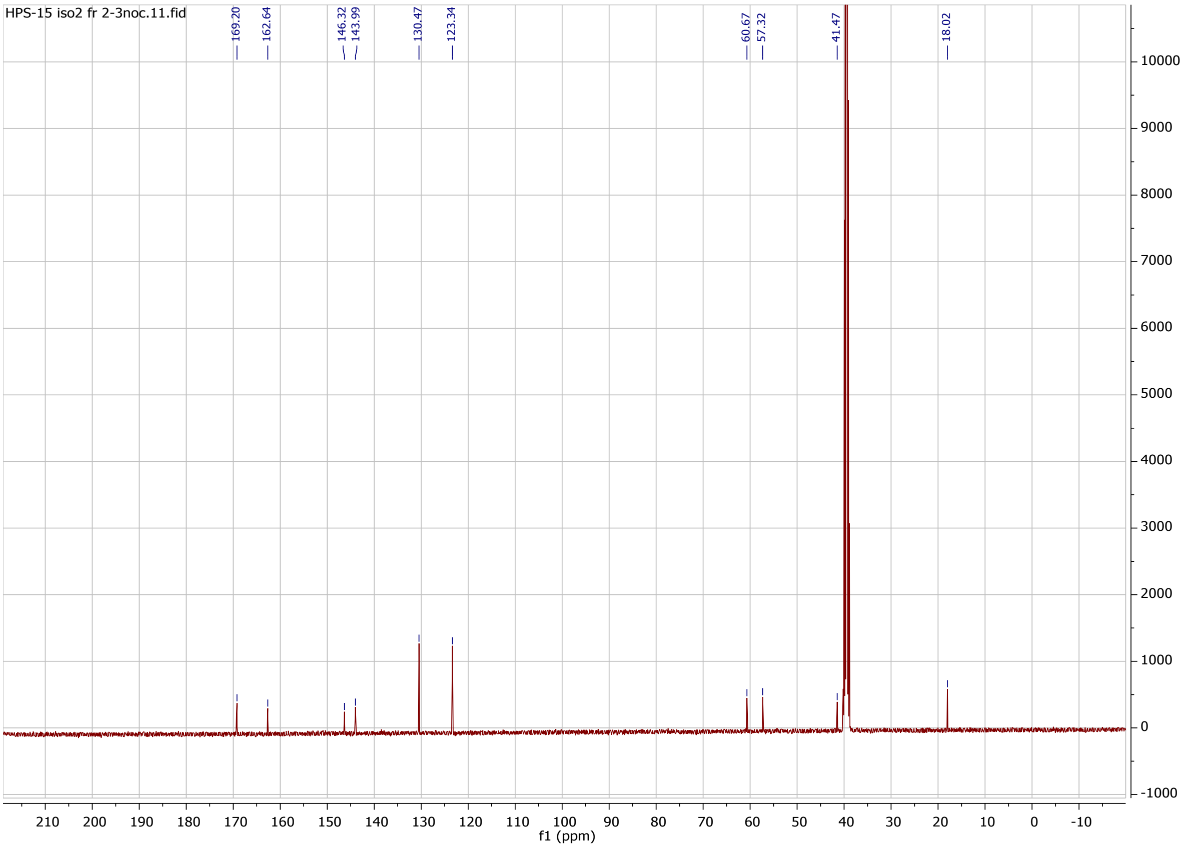

Chromatogram

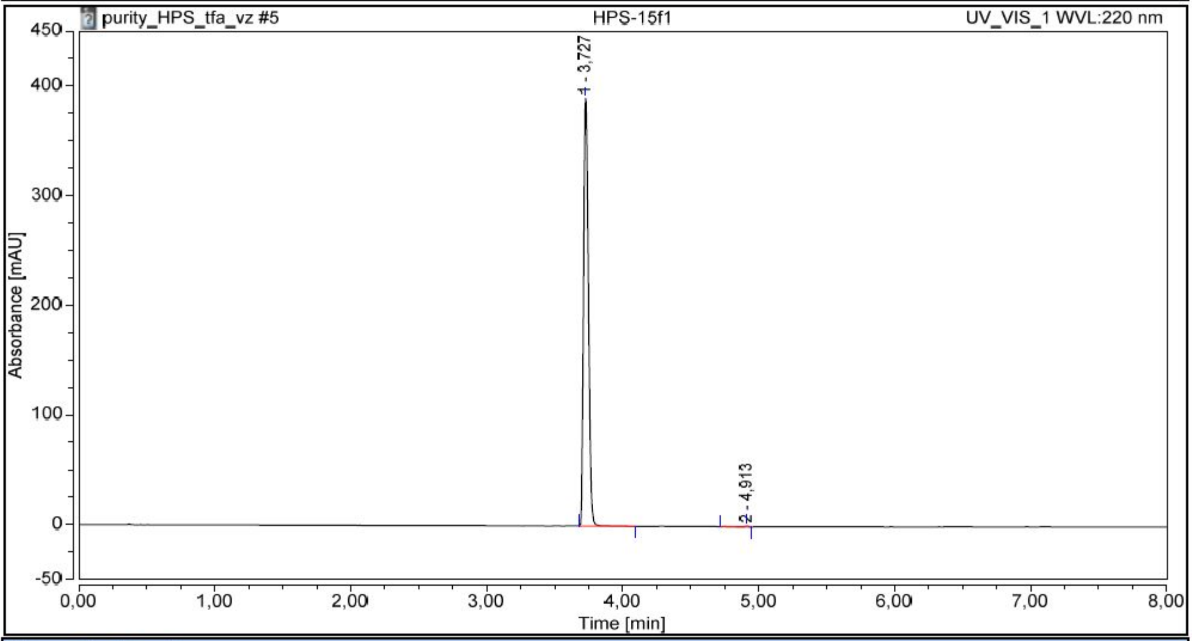

Integration Results

No.	Peak Name	Retention Time min	Area mAU*min	Height mAU	Relative Area %	Relative Height %	Amount n.a.
1		3,727	16,319	389,979	99,58	99,72	n.a.
2		4,913	0,068	1,112	0,42	0,28	n.a.
**Total:**			**16,387**	**391,091**	**100,00**	**100,00**	

HRMS: (ESI–), *m/z* calc. for $C_{12}H_{12}O_7N_3S$ [M–H]$^-$ 342.04014, found 342.04016.

Potassium 3-(2-(2,6-dichlorophenyl)acetamido)-2-methyl-4-oxoazetidine-1-sulfonate (**11**)

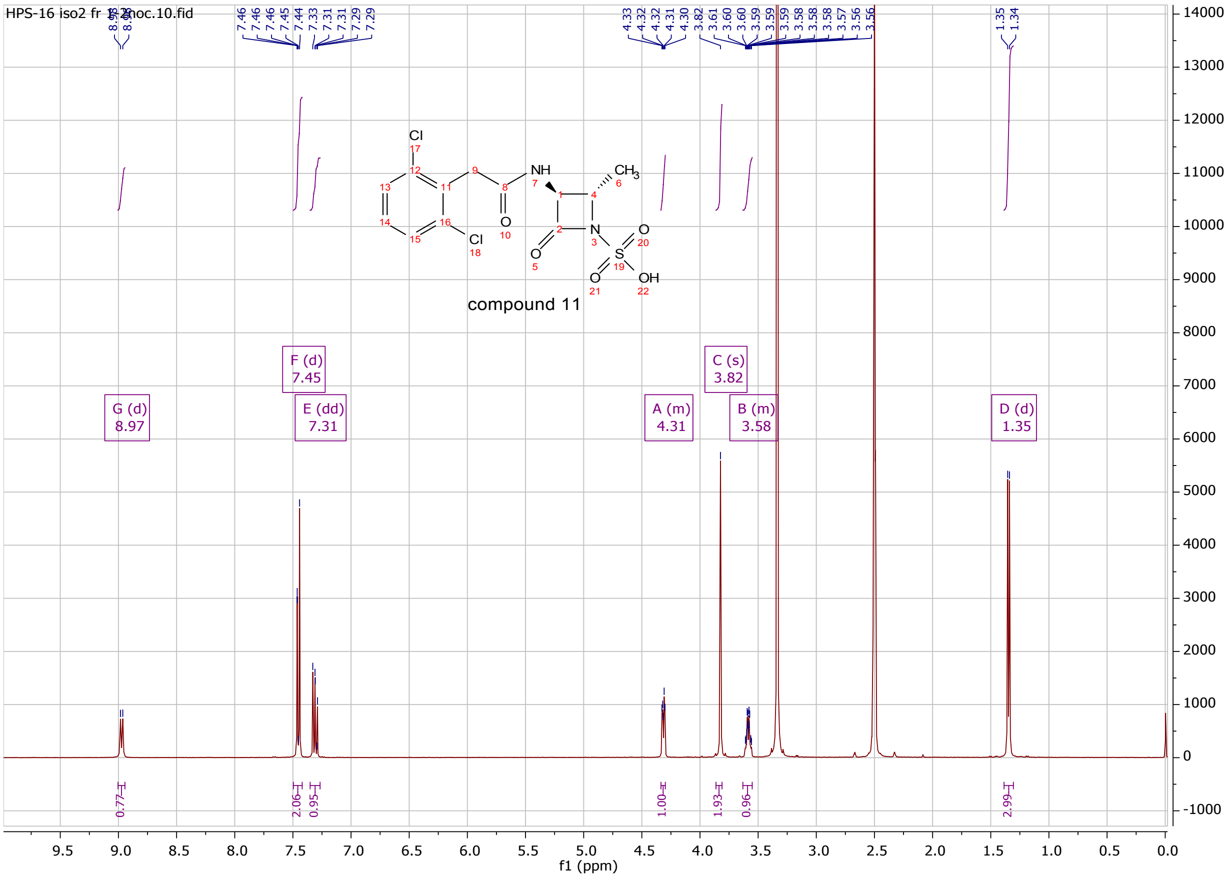

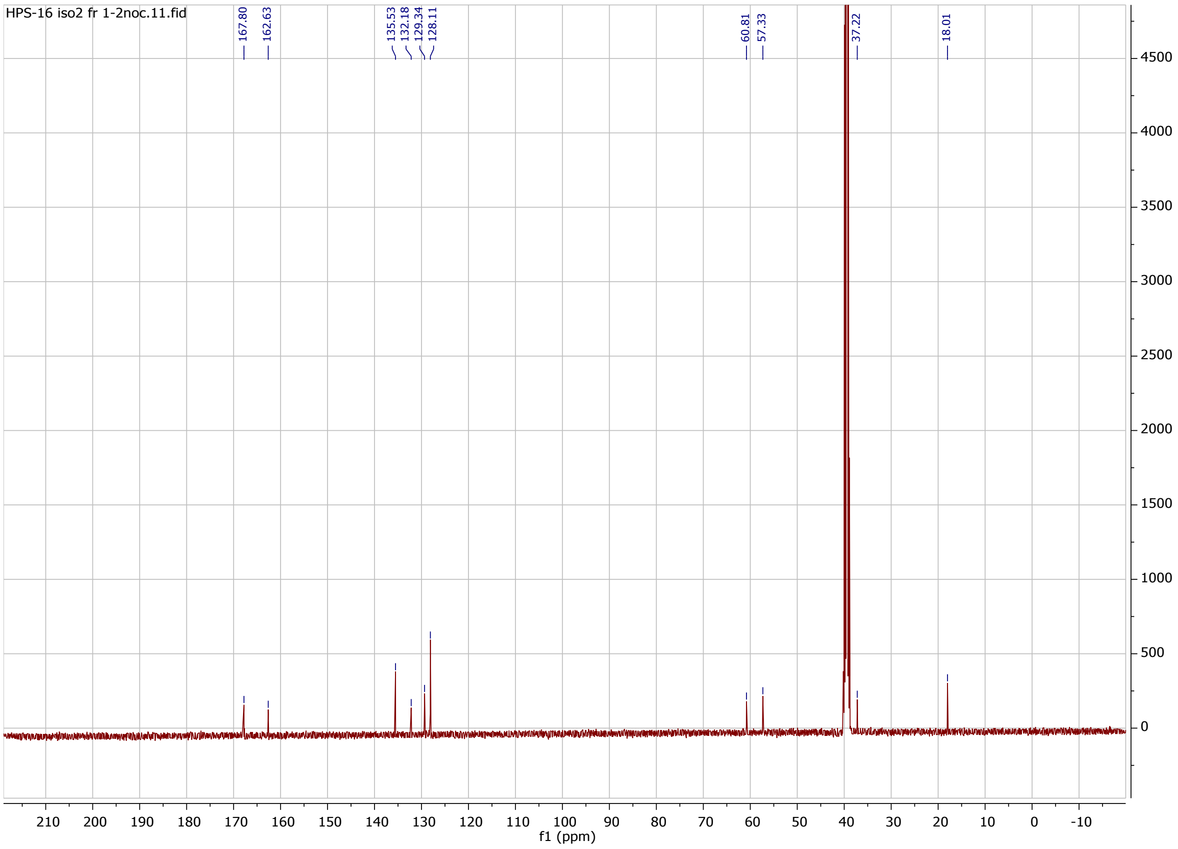

Chromatogram

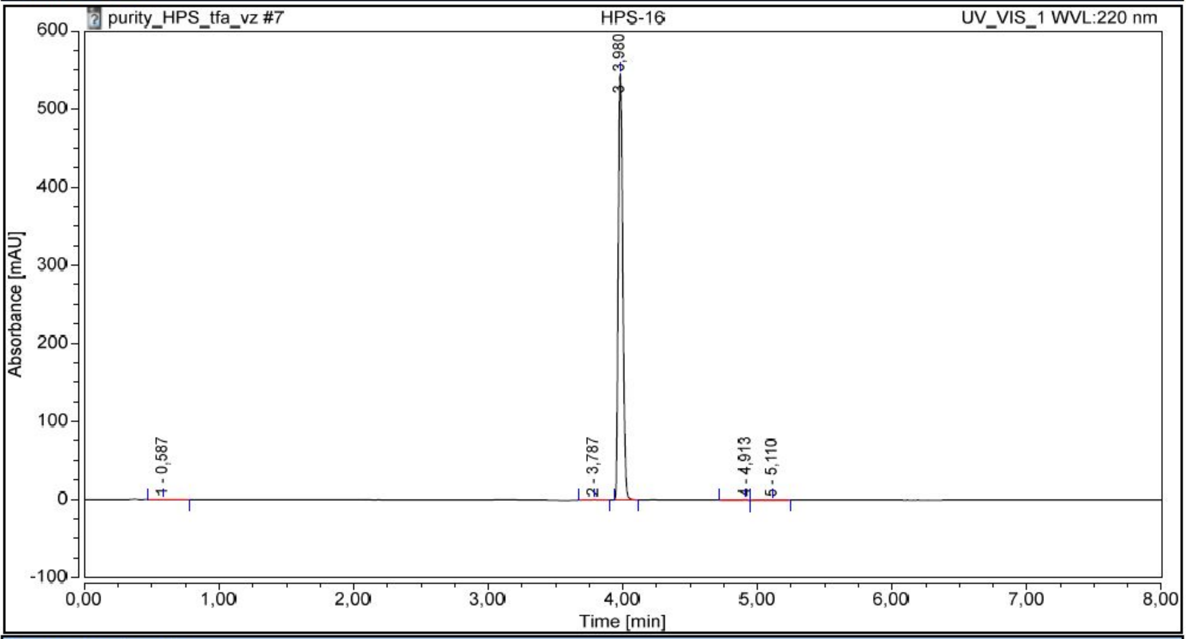

Integration Results

No.	Peak Name	Retention Time<br>min	Area<br>mAU*min	Height<br>mAU	Relative Area<br>%	Relative Height<br>%	Amount<br>n.a.
1		0,587	0,036	0,196	0,17	0,04	n.a.
2		3,787	0,046	0,677	0,21	0,12	n.a.
3		3,980	21,300	546,194	98,93	99,59	n.a.
4		4,913	0,073	1,041	0,34	0,19	n.a.
5		5,110	0,076	0,341	0,35	0,06	n.a.
**Total:**			**21,531**	**548,449**	**100,00**	**100,00**	

HRMS: (ESI–), *m/z* calc. for $C_{12}H_{11}O_5N_2Cl_2S$ $[M–H]^-$ 364.97712, found 364.97718.

Potassium (2*S*,3*S*)-3-(2-(4-fluorophenyl)acetamido)-2-methyl-4-oxoazetidine-1-sulfonate (**12**)

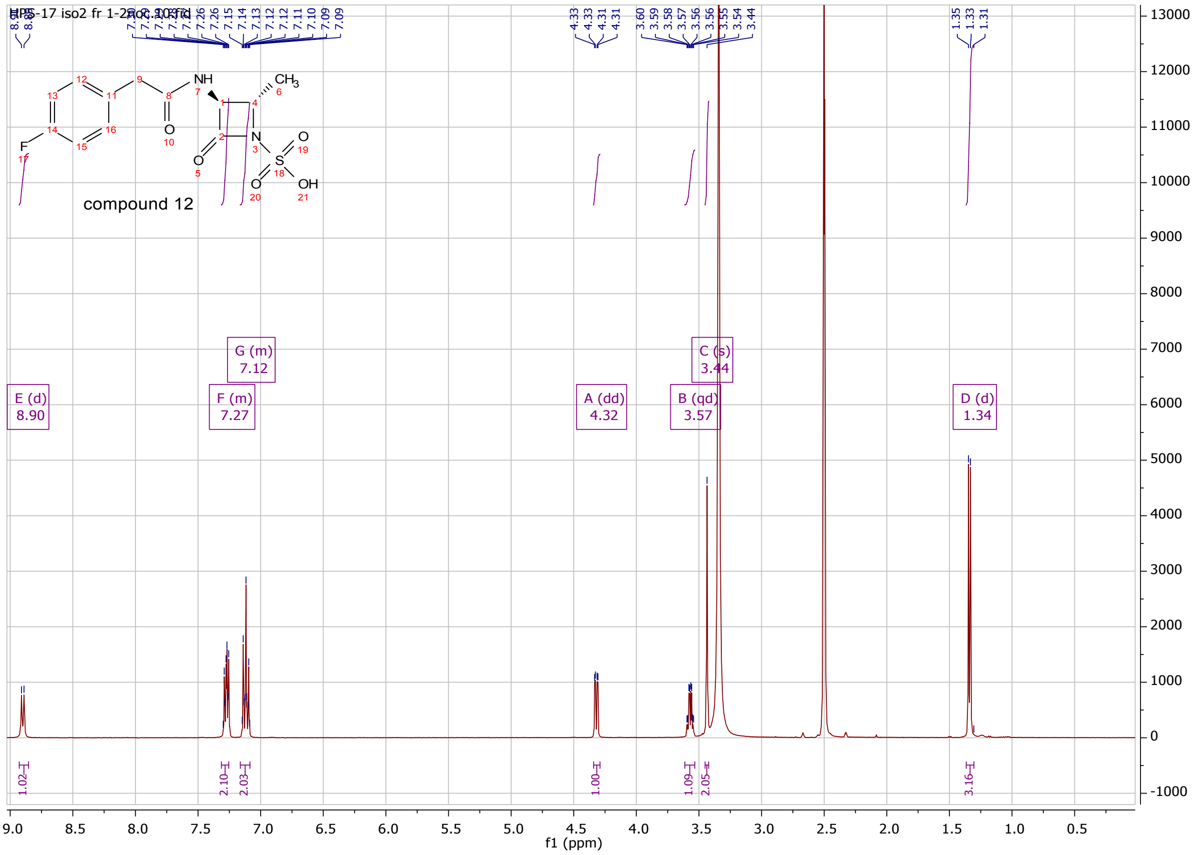

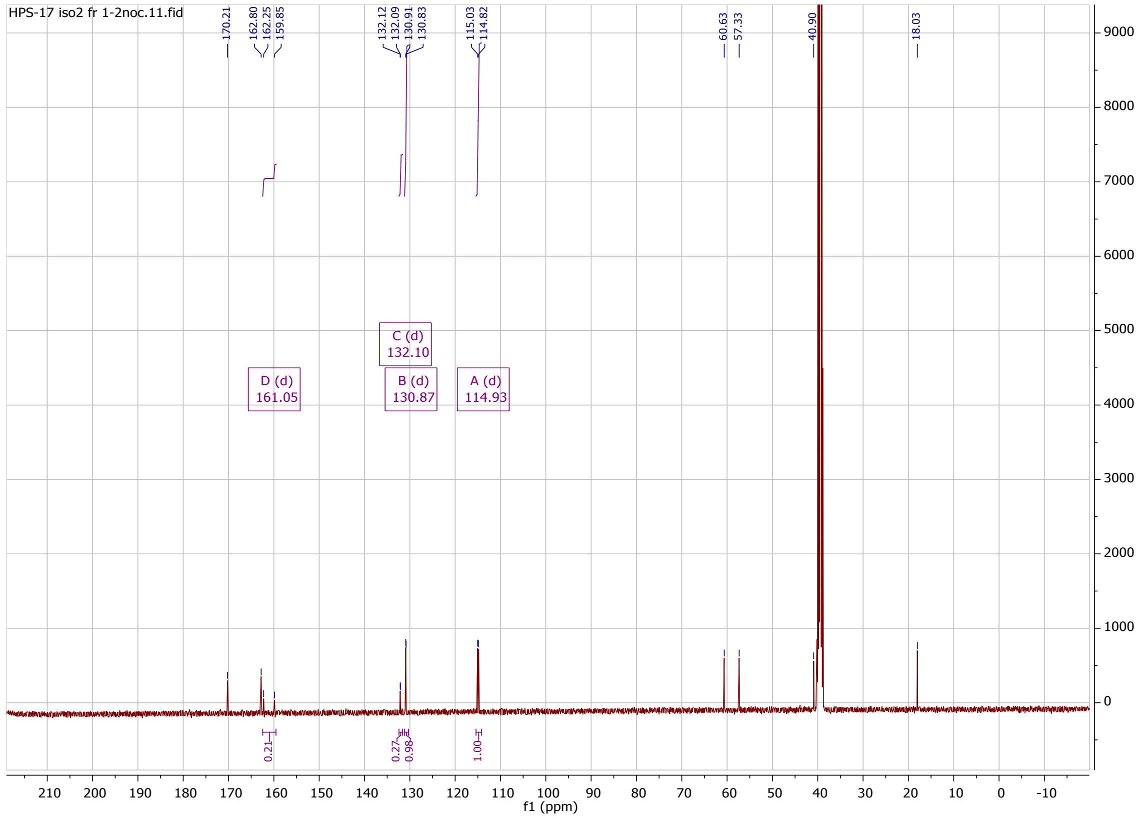

HRMS: (ESI-), *m/z* calc. for $C_{12}H_{12}O_5N_2FS$ $[M-H]^-$ 315.04564, found 315.04570.

Potassium (2*S*,3*S*)-3-(2-(4-chlorophenyl)acetamido)-2-methyl-4-oxoazetidine-1-sulfonate (13)

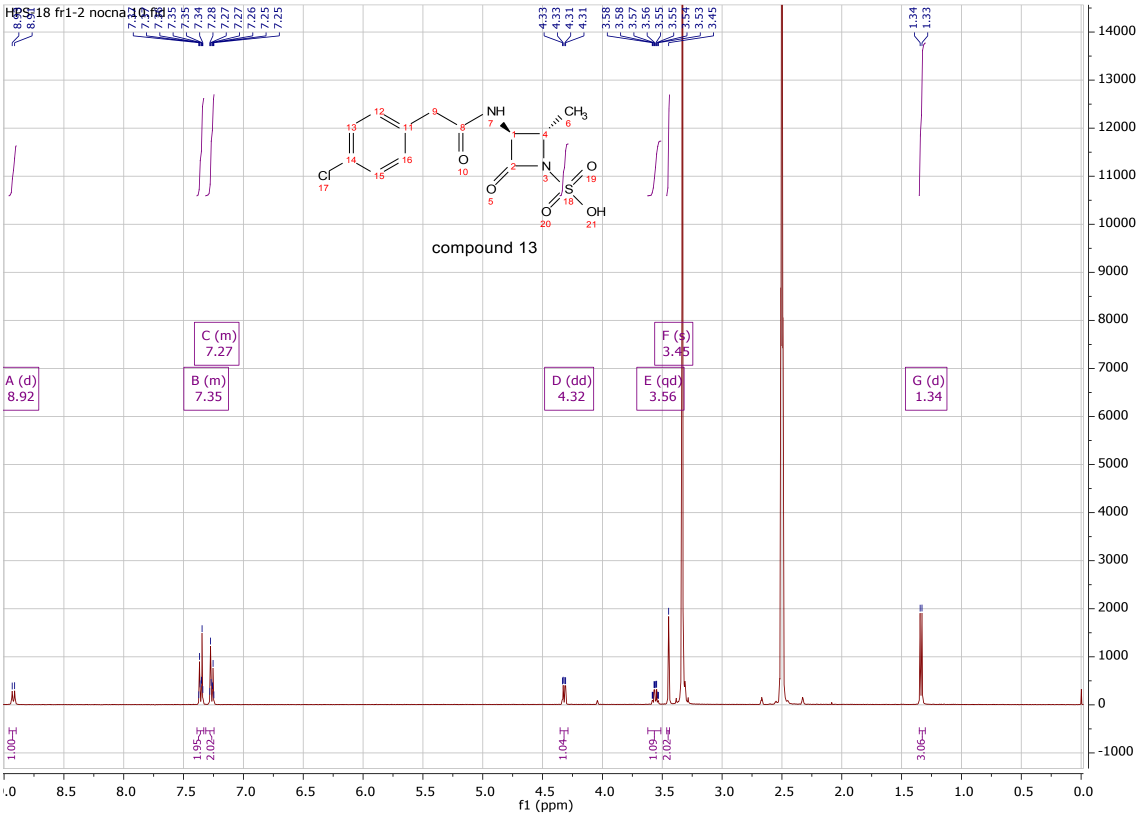

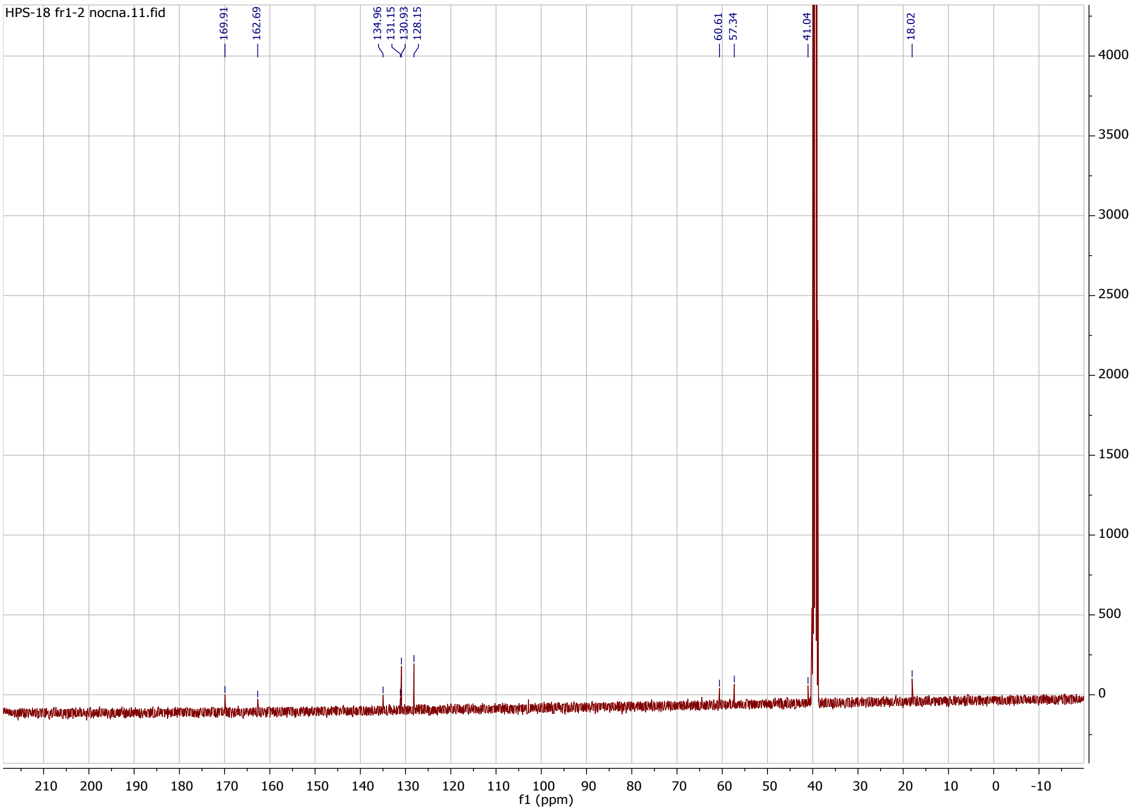

HRMS: (ESI–), *m/z* calc. for $C_{12}H_{12}O_5N_2ClS$ $[M–H]^-$ 331.01609, found 331.01639.

Potassium (2*S*,3*S*)-3-(2-(4-bromophenyl)acetamido)-2-methyl-4-oxoazetidine-1-sulfonate (14)

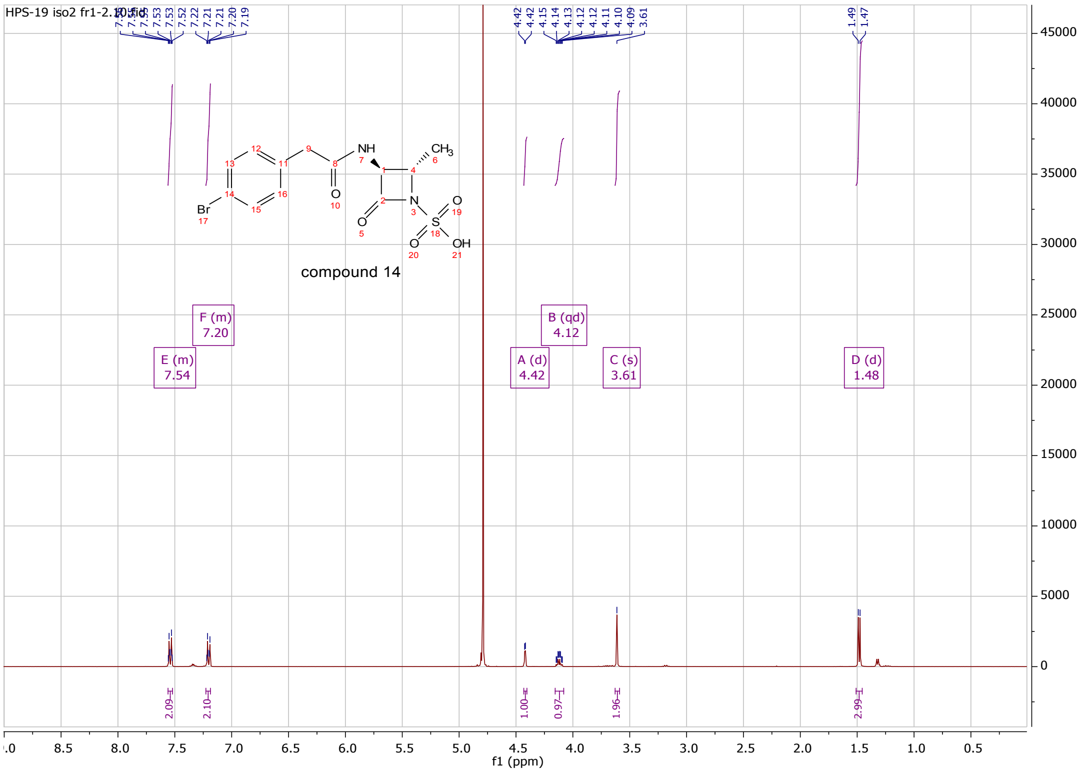

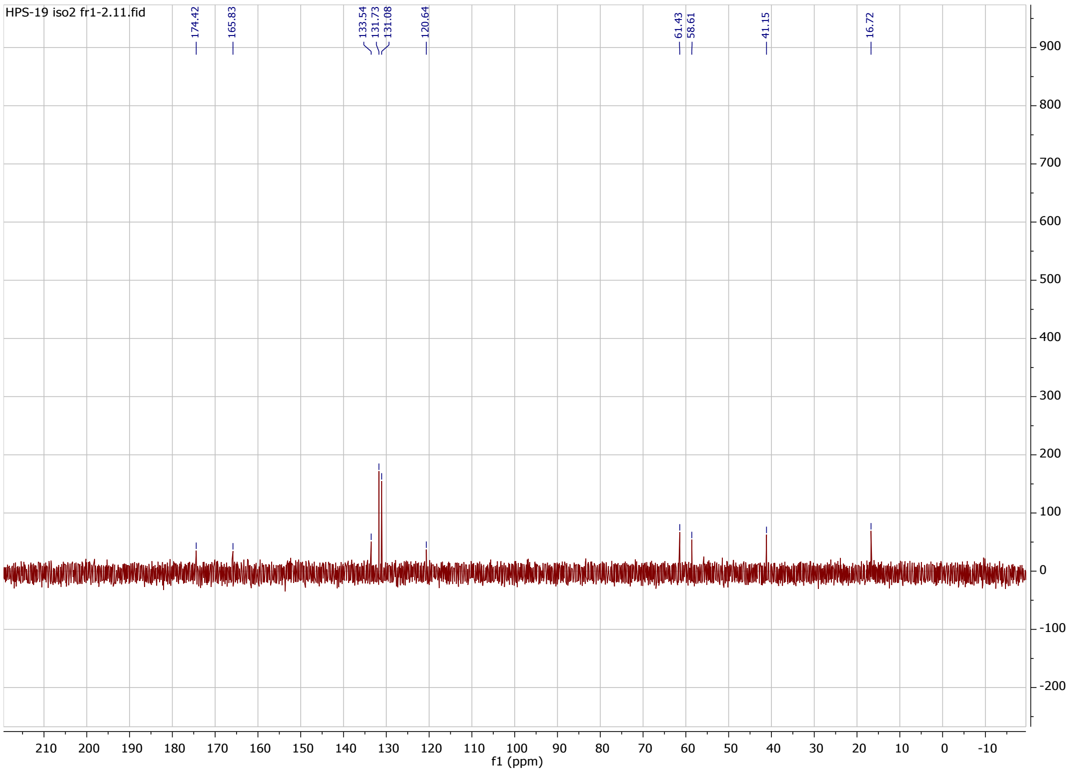

HRMS: (ESI–), *m/z* calc. for $C_{12}H_{12}O_5N_2BrS$ $[M–H]^-$ 374.96558, found 374.96564.

Potassium (2*S*,3*S*)-3-(2-methoxy-2-phenylacetamido)-2-methyl-4-oxoazetidine-1-sulfonate (**15**)

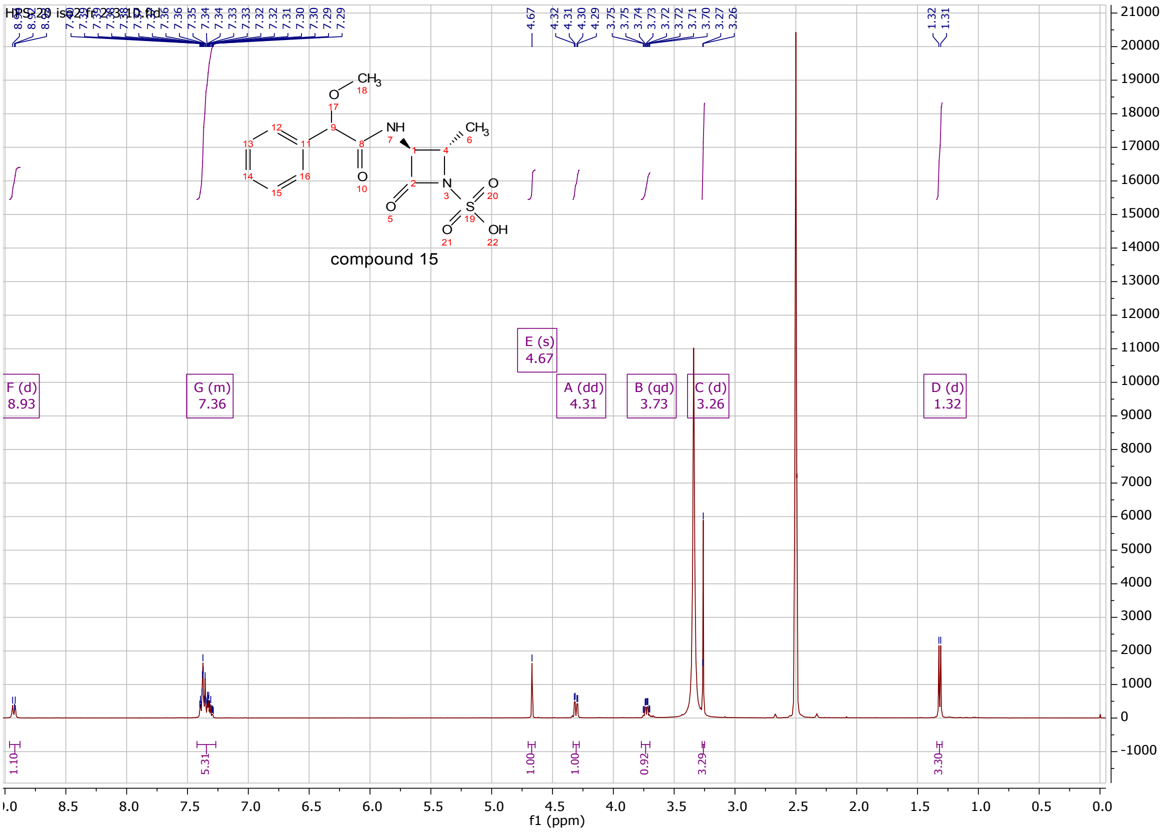

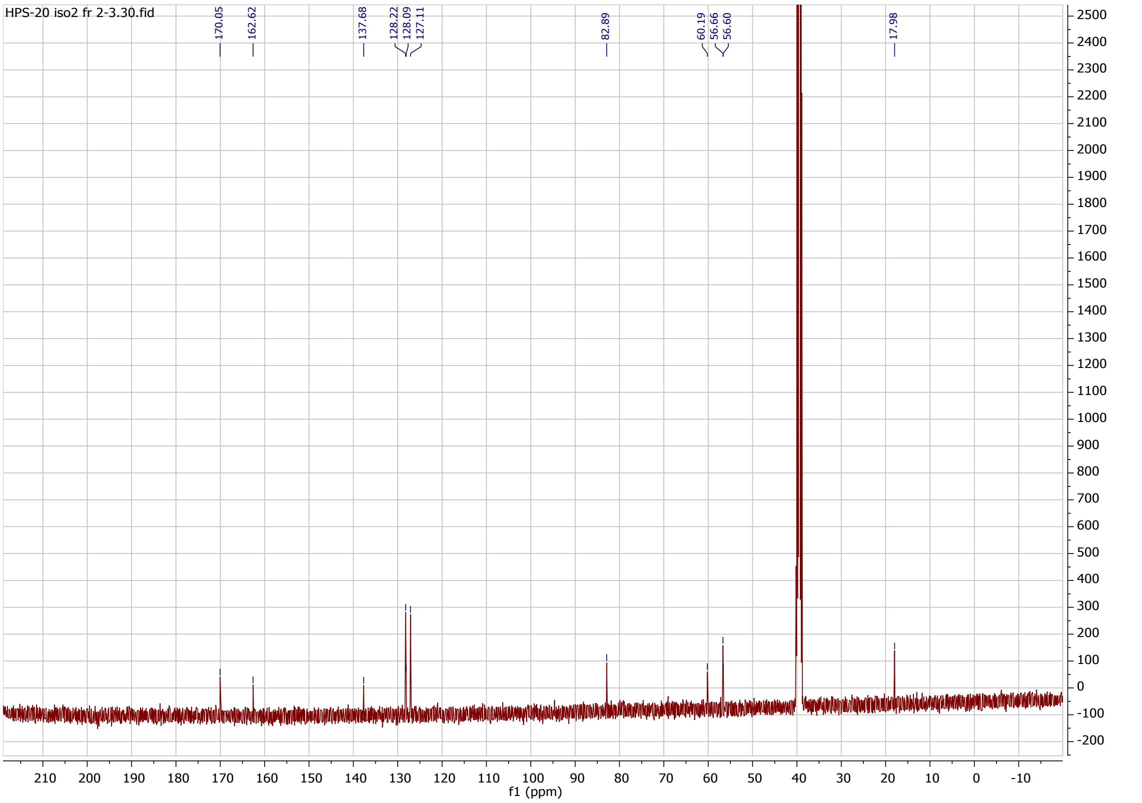

HRMS: (ESI–), *m/z* calc. for $C_{13}H_{15}O_6N_2S$ $[M–H]^-$ 327.06563, found 327.06557.

Potassium (2*S*,3*S*)-3-(2-(3,4-dimethoxyphenyl)acetamido)-2-methyl-4-oxoazetidine-1-sulfonate (**16**)

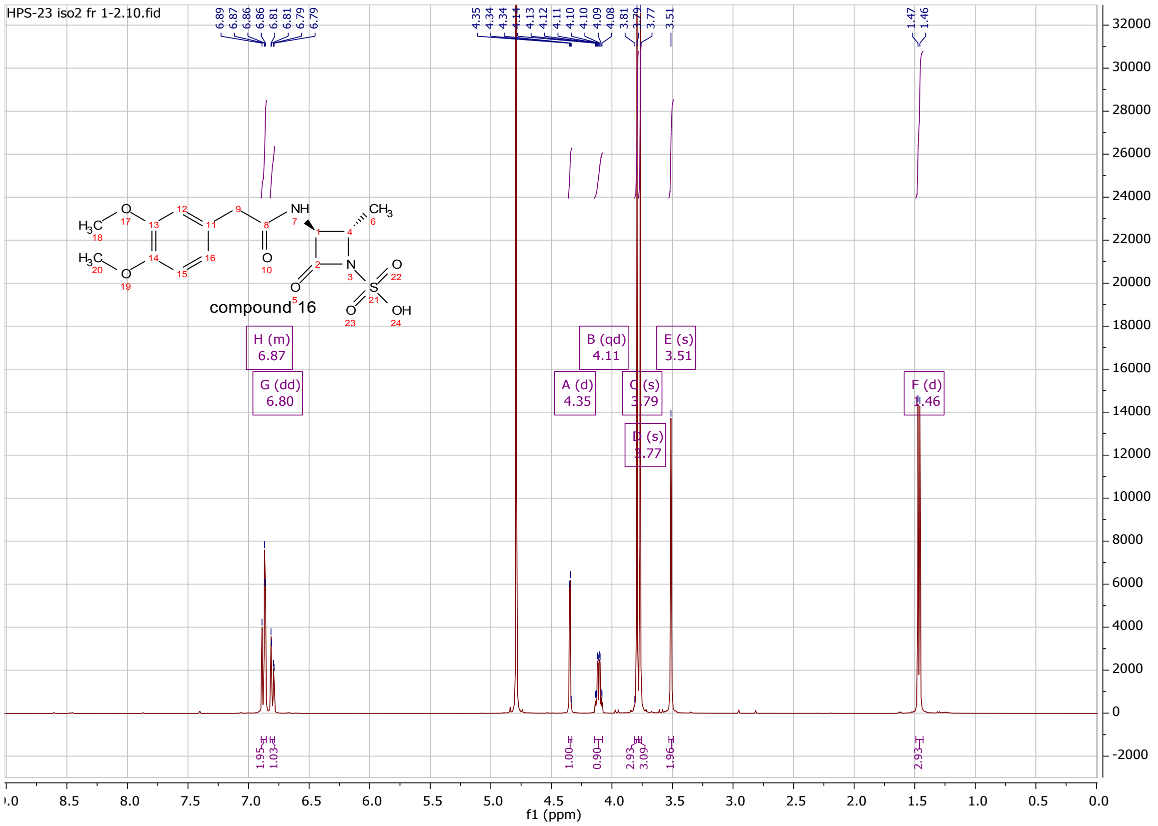

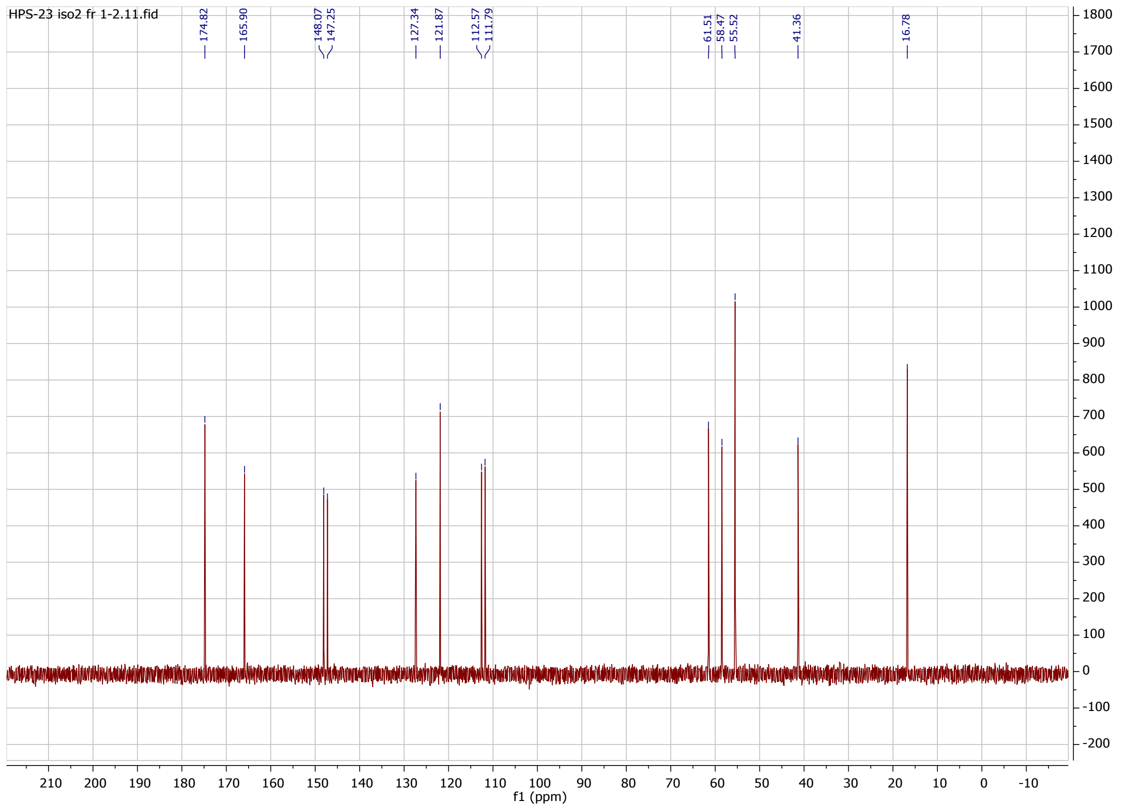

HRMS: (ESI–), *m/z* calc. for $C_{14}H_{17}O_7N_2S$ $[M–H]^-$ 357.07619, found 357.07619.

Potassium (2*S*,3*S*)-2-methyl-4-oxo-3-(2-(thiophen-2-yl)acetamido)azetidine-1-sulfonate (**17**)

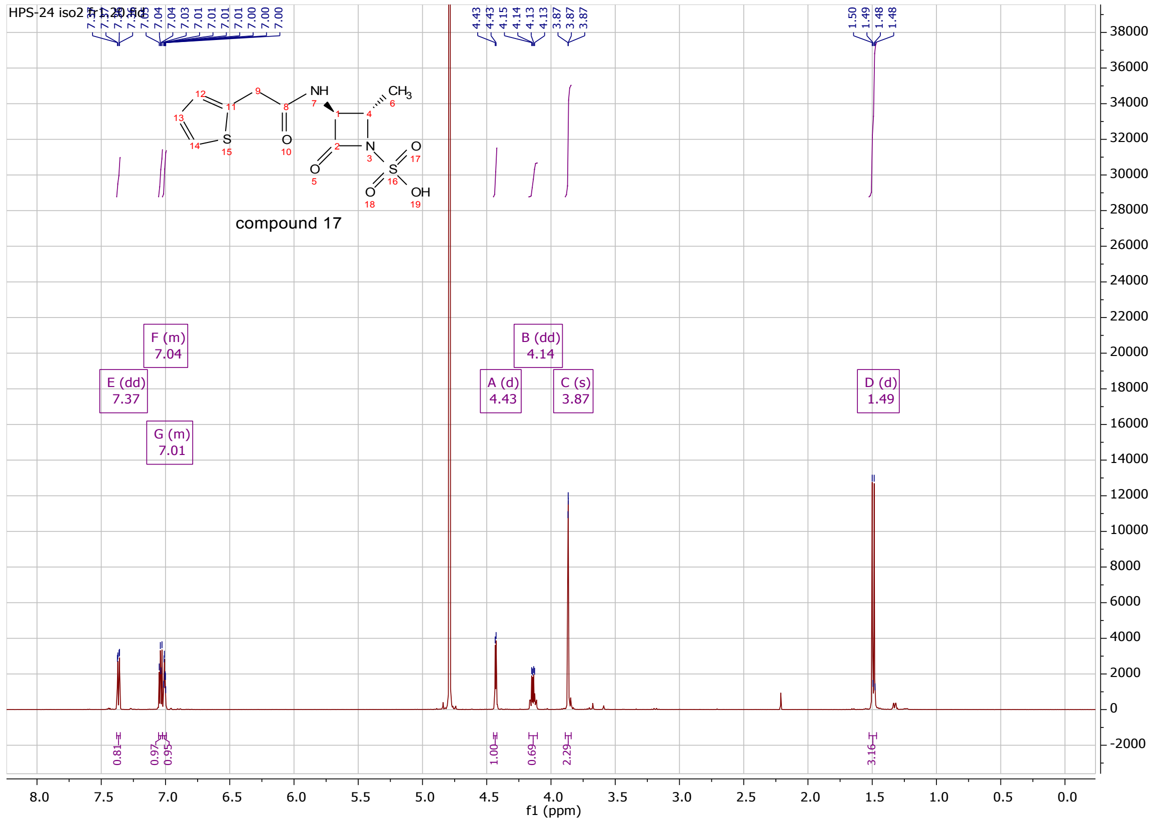

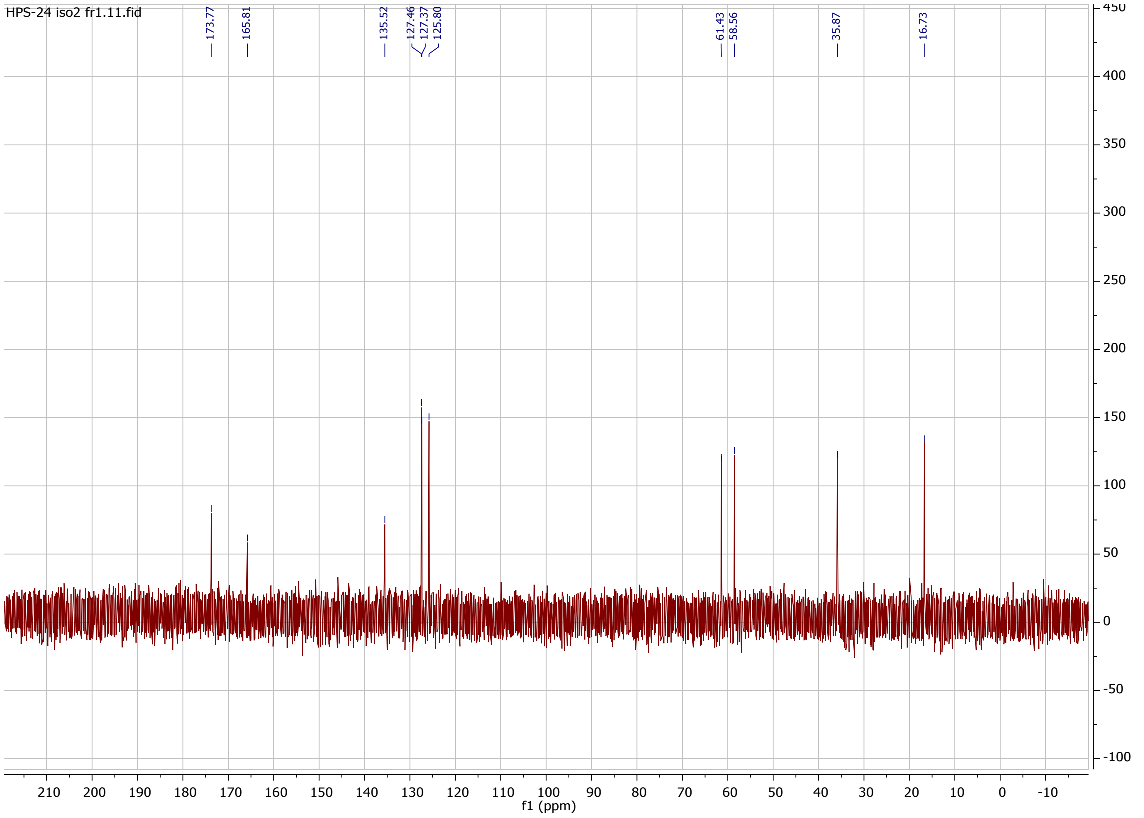

HRMS: (ESI–), *m/z* calc. for $C_{10}H_{11}O_5N_2S_2$ $[M–H]^-$ 303.01149, found 303.01159.

Potassium (2*S*,3*S*)-2-methyl-4-oxo-3-(2-(pyridin-4-yl)acetamido)azetidine-1-sulfonate (**18**)

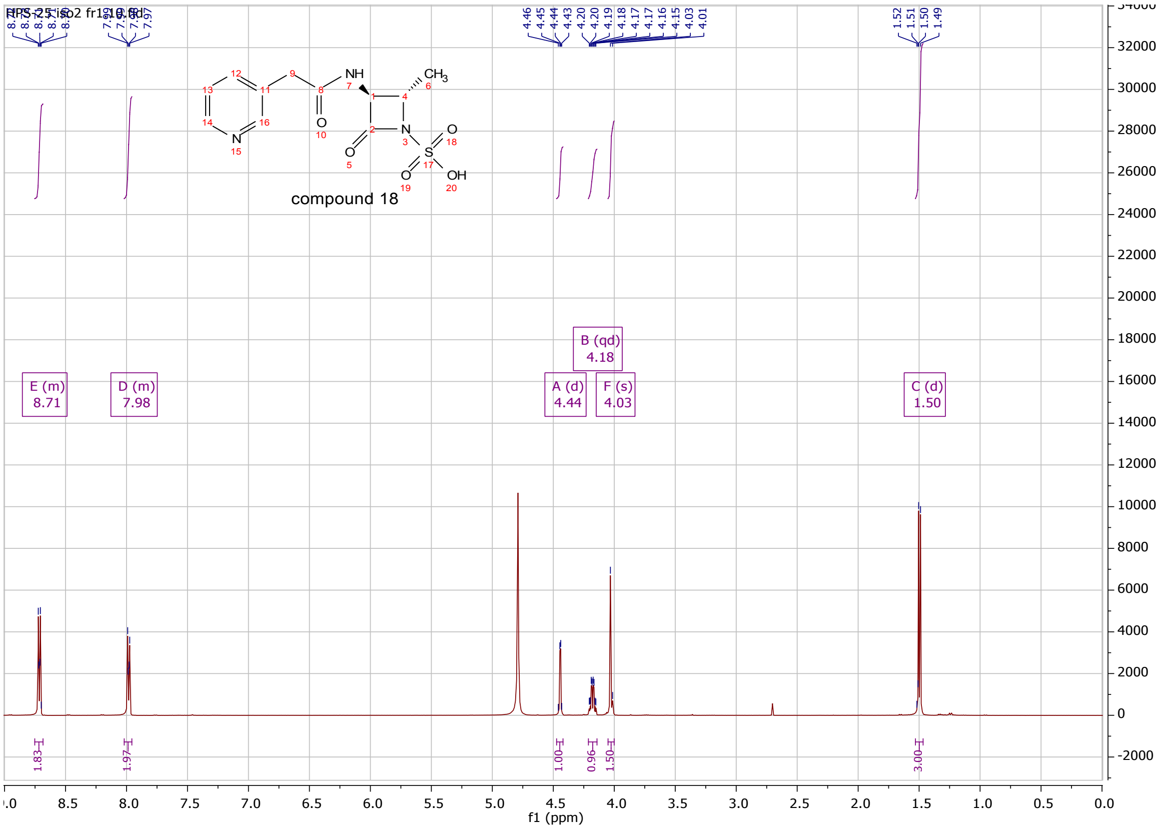

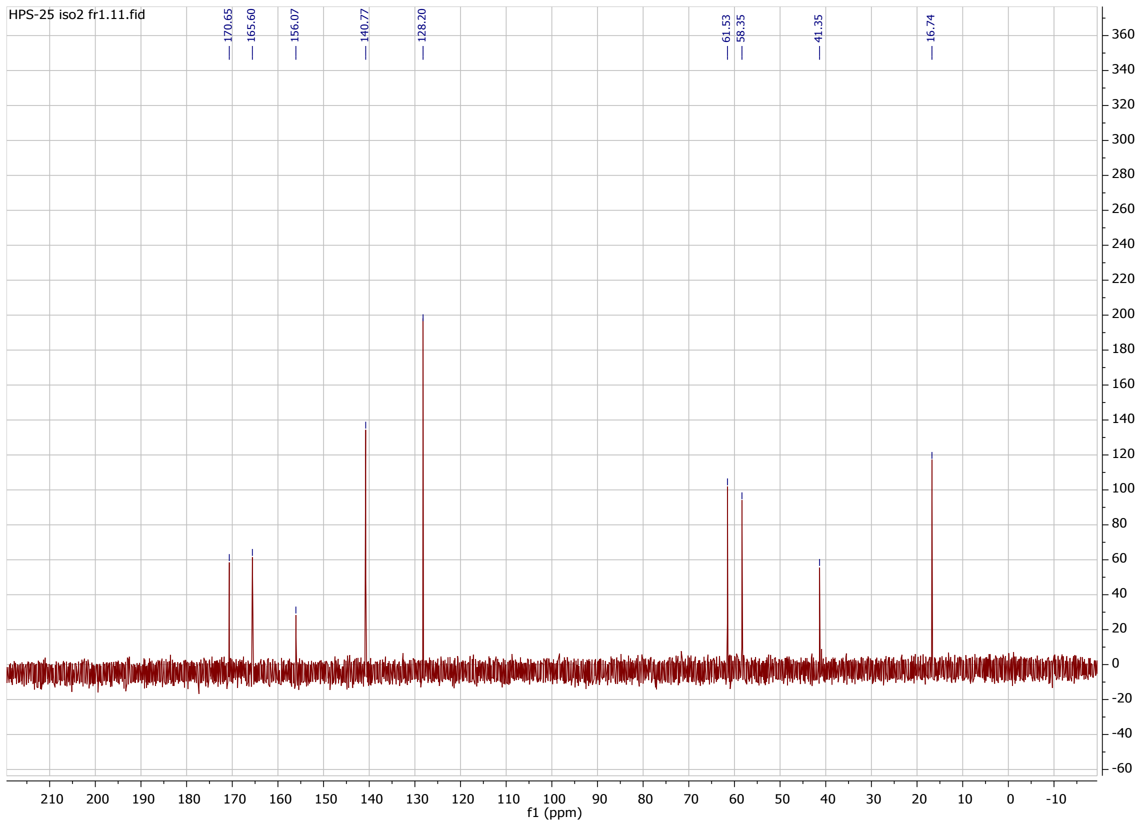

HRMS: (ESI–), *m/z* calc. for $C_{11}H_{12}O_5N_3S$ $[M–H]^-$ 298.05031, found 298.05060.

Potassium (2*S*,3*S*)-3-(2-((tert-butoxycarbonyl)amino)-2-phenylacetamido)-2-methyl-4-oxoazetidine-1-sulfonate (**19**)

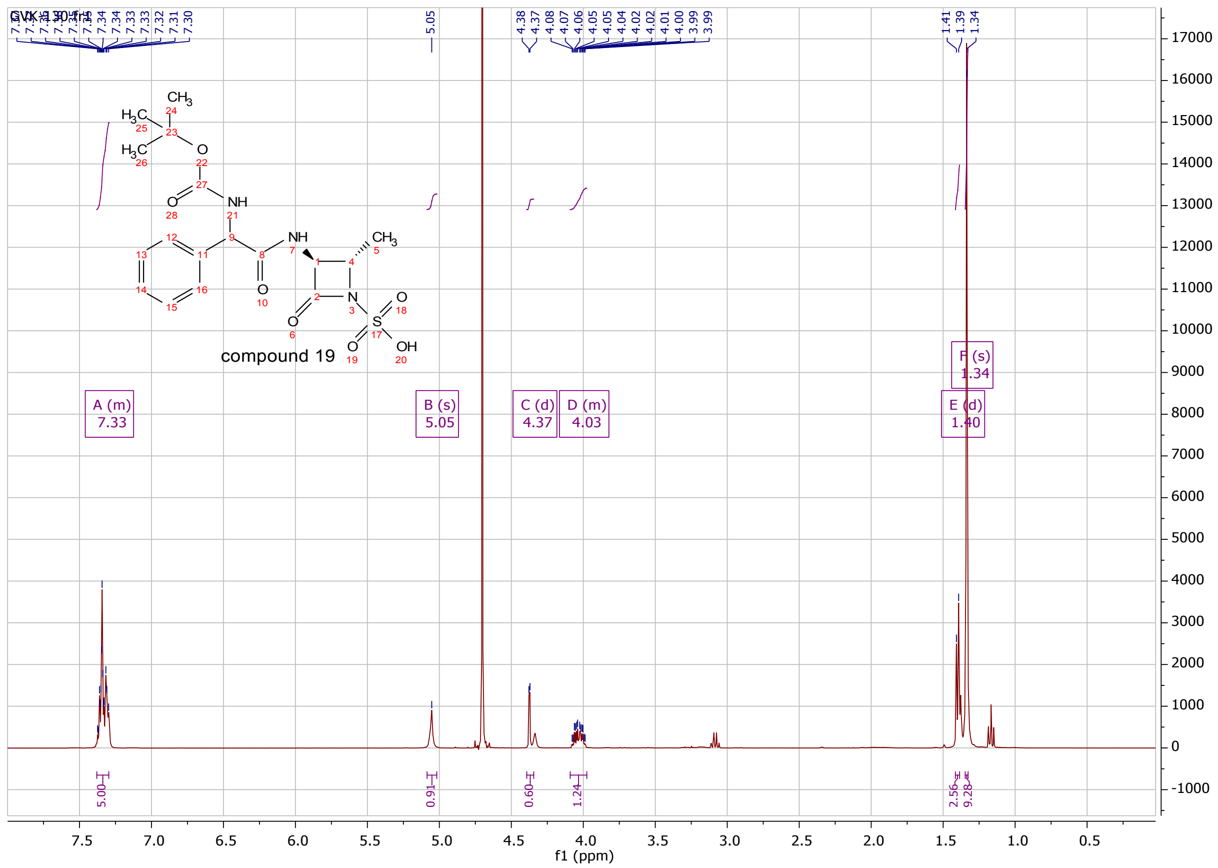

HRMS: (ESI–), *m/z* calc. for $C_{17}H_{22}O_7N_3S$ $[M–H]^-$ 412.11839, found 412.11781.

Potassium (2*S*,3*S*)-3-(2-amino-2-phenylacetamido)-2-methyl-4-oxoazetidine-1-sulfonate (**20**)

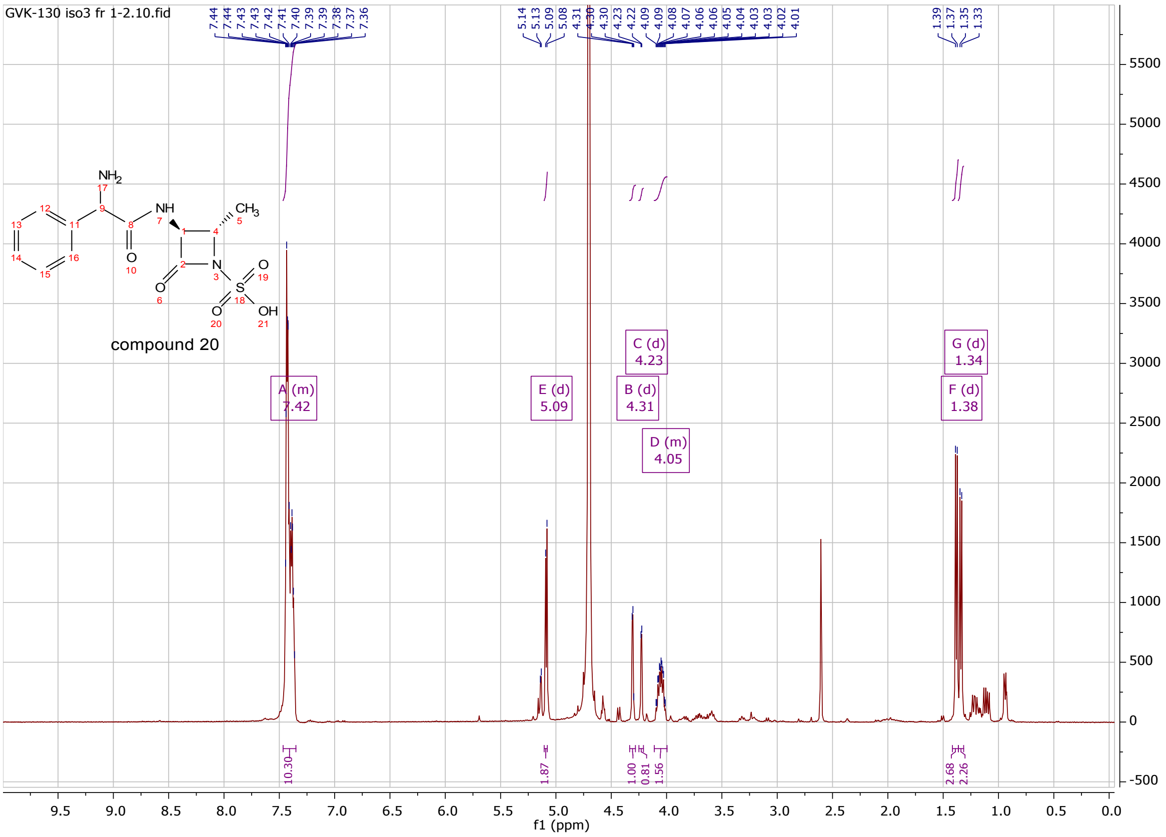

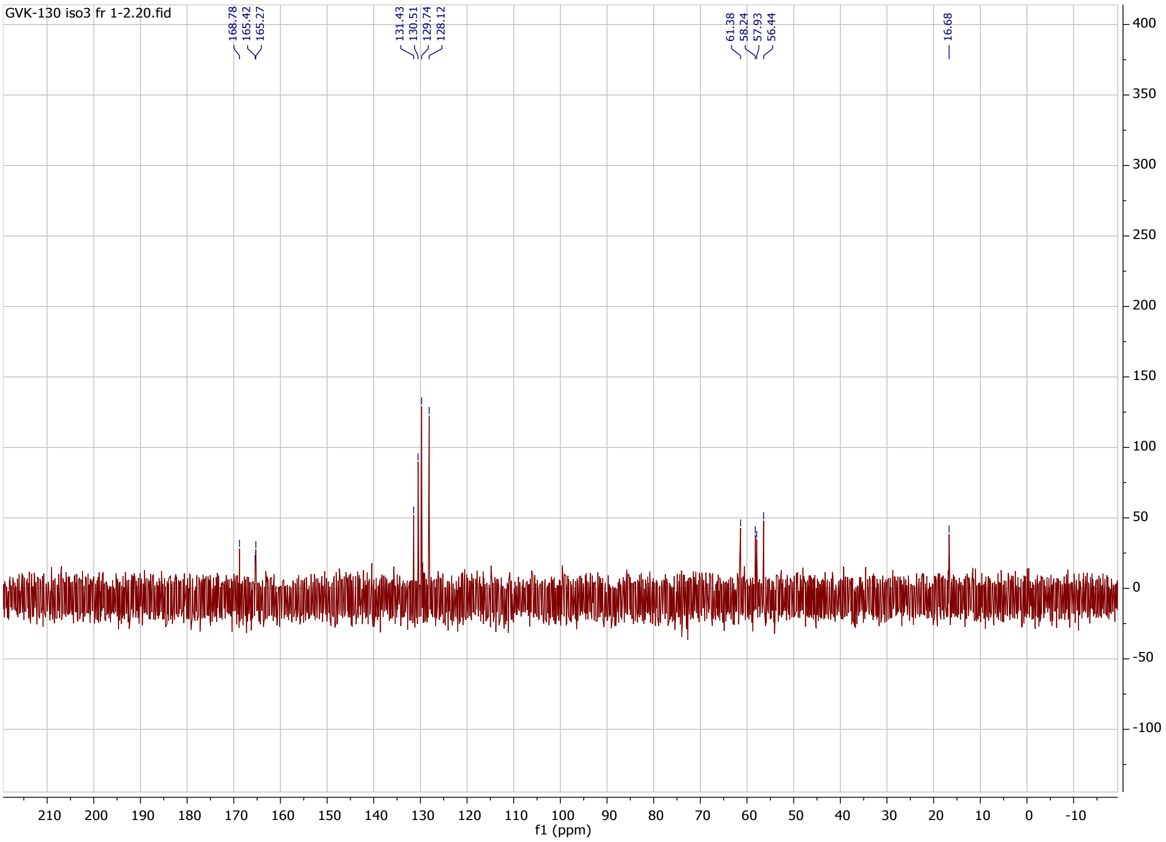

HRMS: (ESI–), *m/z* calc. for $C_{12}H_{14}O_5N_3S$ $[M–H]^-$ 312.06596, found 312.06561.

Potassium (2*S*,3*S*)-2-methyl-4-oxo-3-(2-phenylacetamido)azetidine-1-sulfonate (**21**)

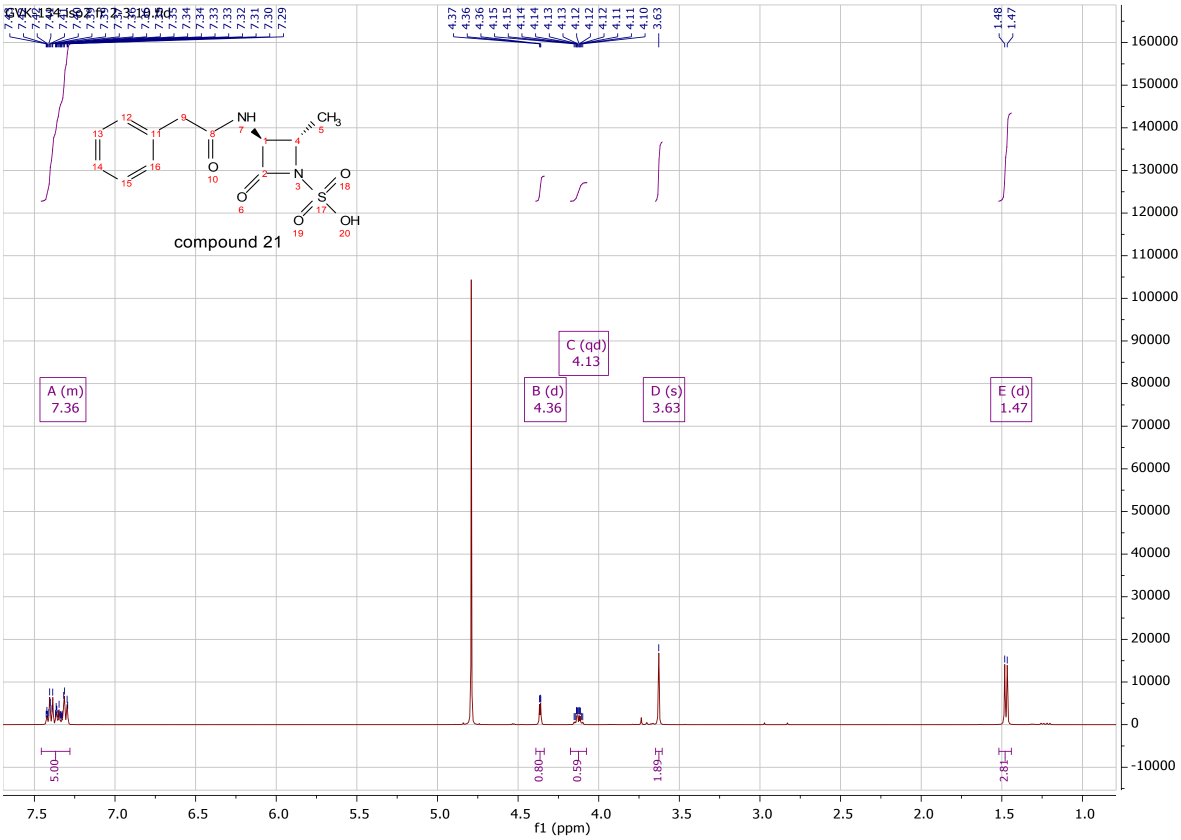

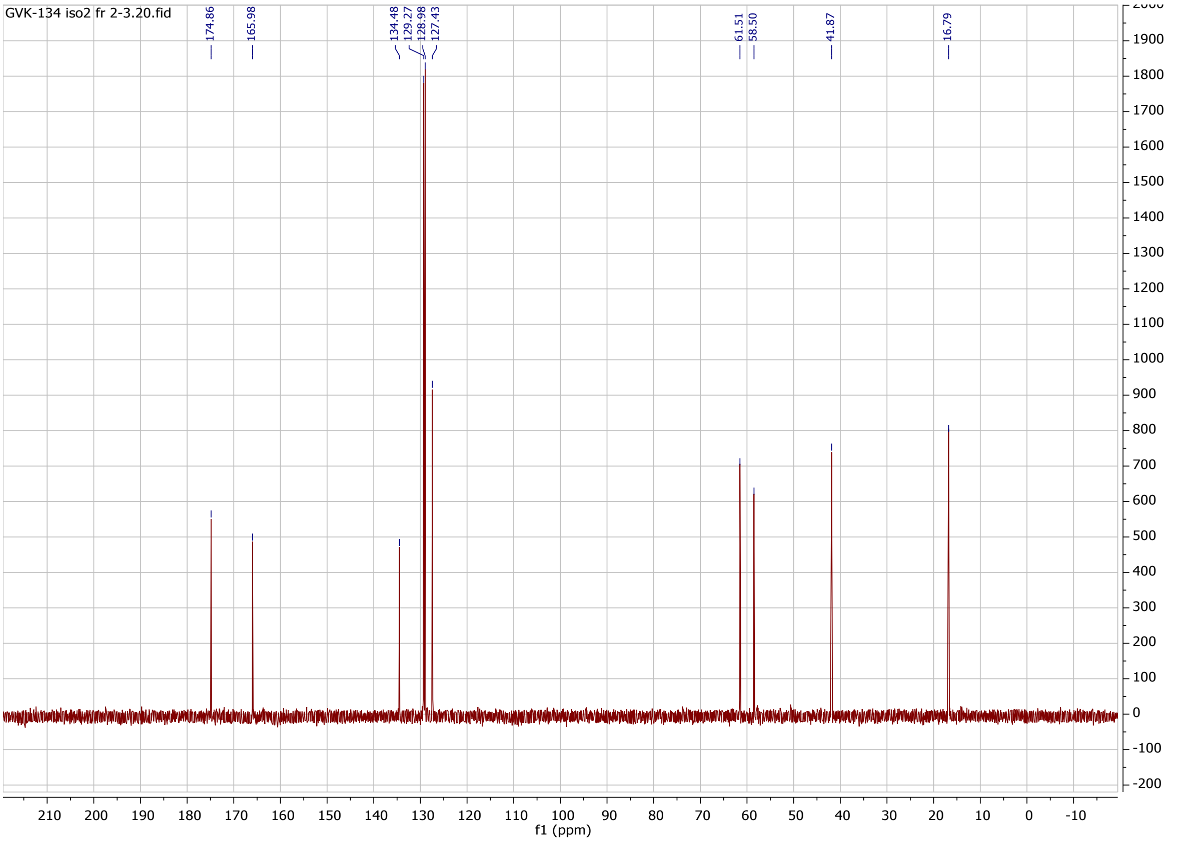

HRMS: (ESI–), calc. for $C_{12}H_{13}O_5N_2S$ $[M–H]^-$ 297.05397, found 297.05450.

(2*S*,3*S*)-3-((*Z*)-2-(2-aminothiazol-4-yl)-2-((2-(*tert*-butoxy)-2-oxoethoxy)imino)acetamido)-2-methyl-4-oxoazetidine-1-sulfonic acid, DIPEA salt (**22**)

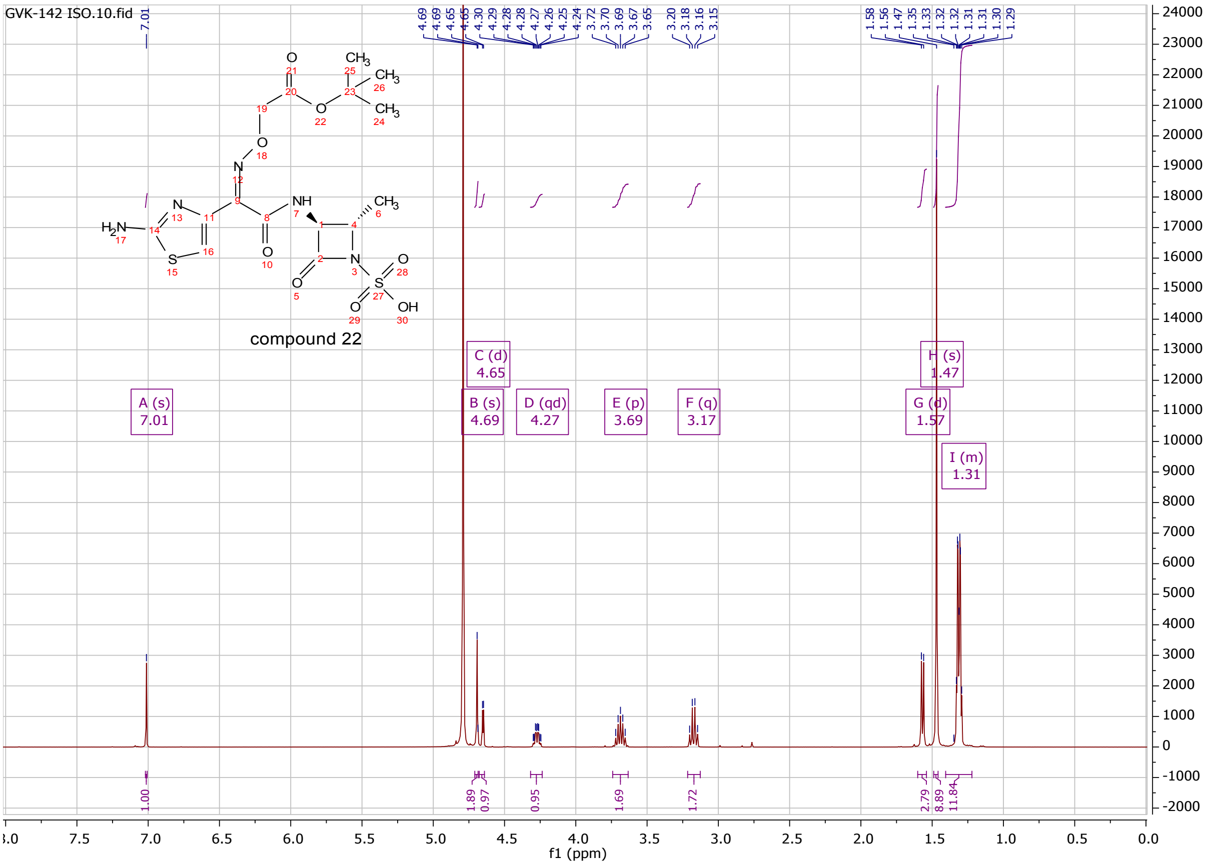

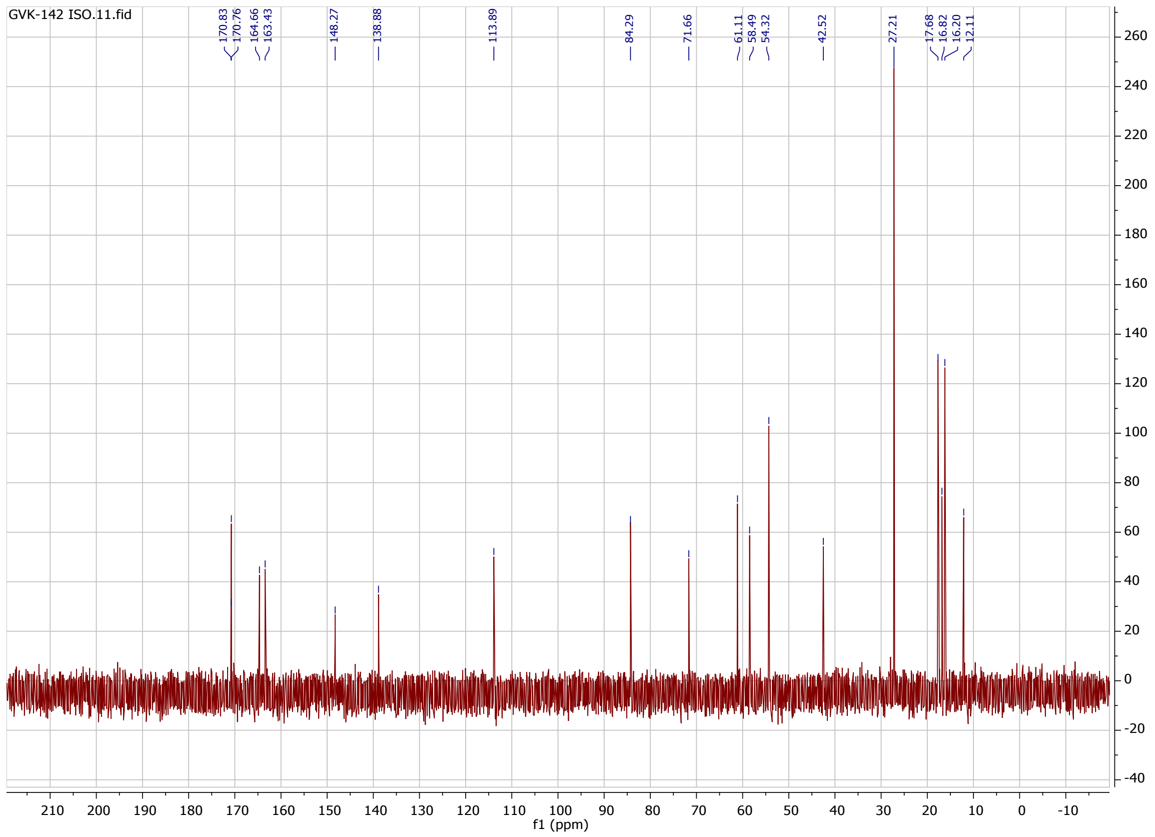

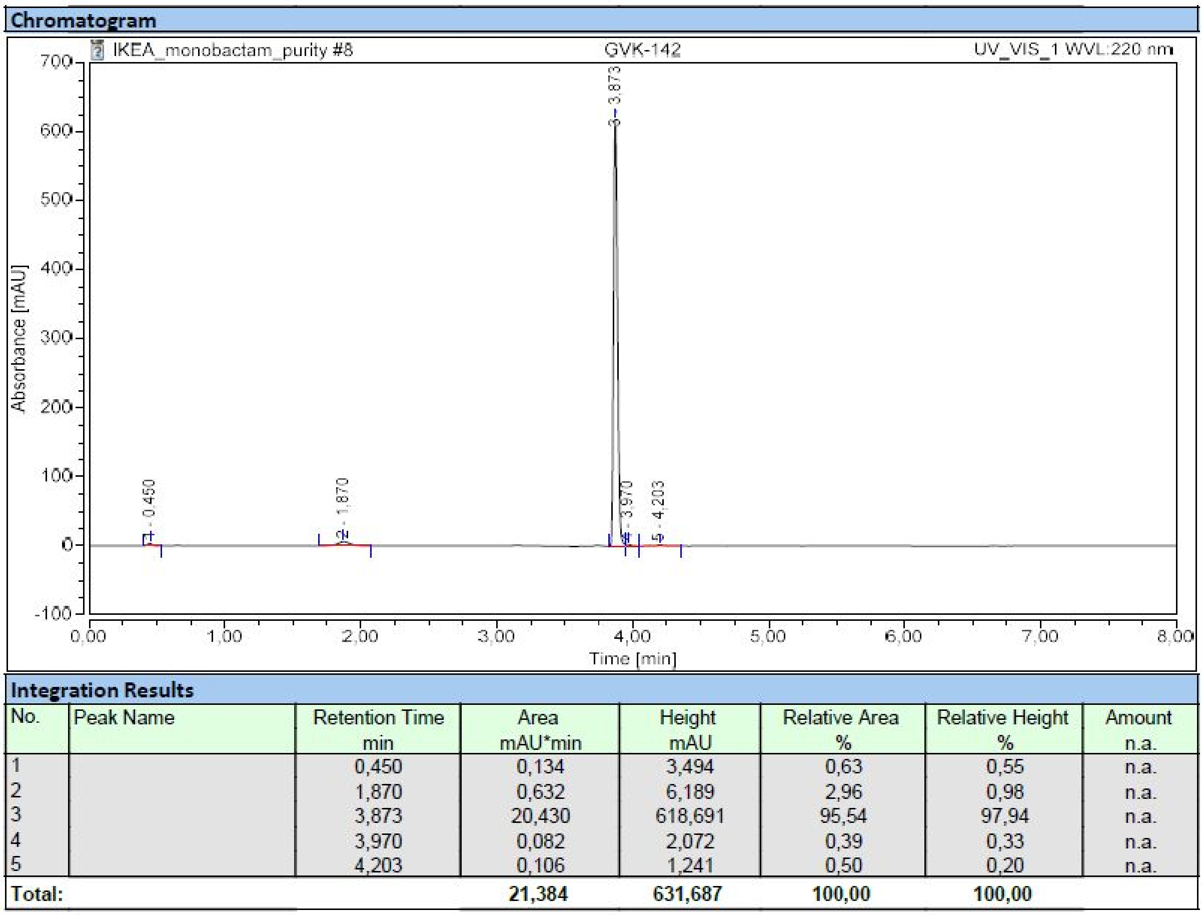

Integration Results

No.	Peak Name	Retention Time<br>min	Area<br>mAU*min	Height<br>mAU	Relative Area<br>%	Relative Height<br>%	Amount<br>n.a.
1		0,450	0,134	3,494	0,63	0,55	n.a.
2		1,870	0,632	6,189	2,96	0,98	n.a.
3		3,873	20,430	618,691	95,54	97,94	n.a.
4		3,970	0,082	2,072	0,39	0,33	n.a.
5		4,203	0,106	1,241	0,50	0,20	n.a.
**Total:**			**21,384**	**631,687**	**100,00**	**100,00**	

HRMS: (ESI–), calc. for $C_{15}H_{21}O_8N_5S_2$ $[M–H]^-$ 462.07478, found 462.07642.

Potassium (2*S*,3*S*)-3-(2-(2,5-dimethylthiazol-4-yl)acetamido)-2-methyl-4-oxoazetidine-1-sulfonate (**23**)

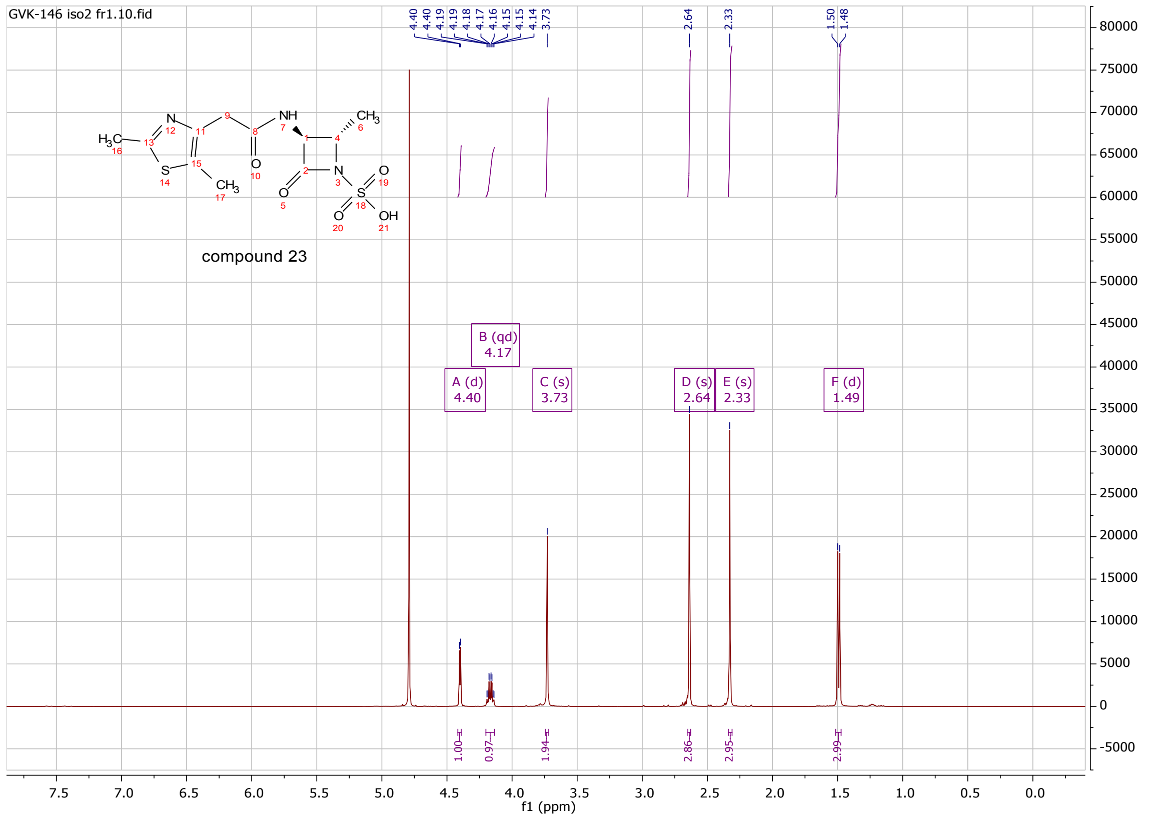

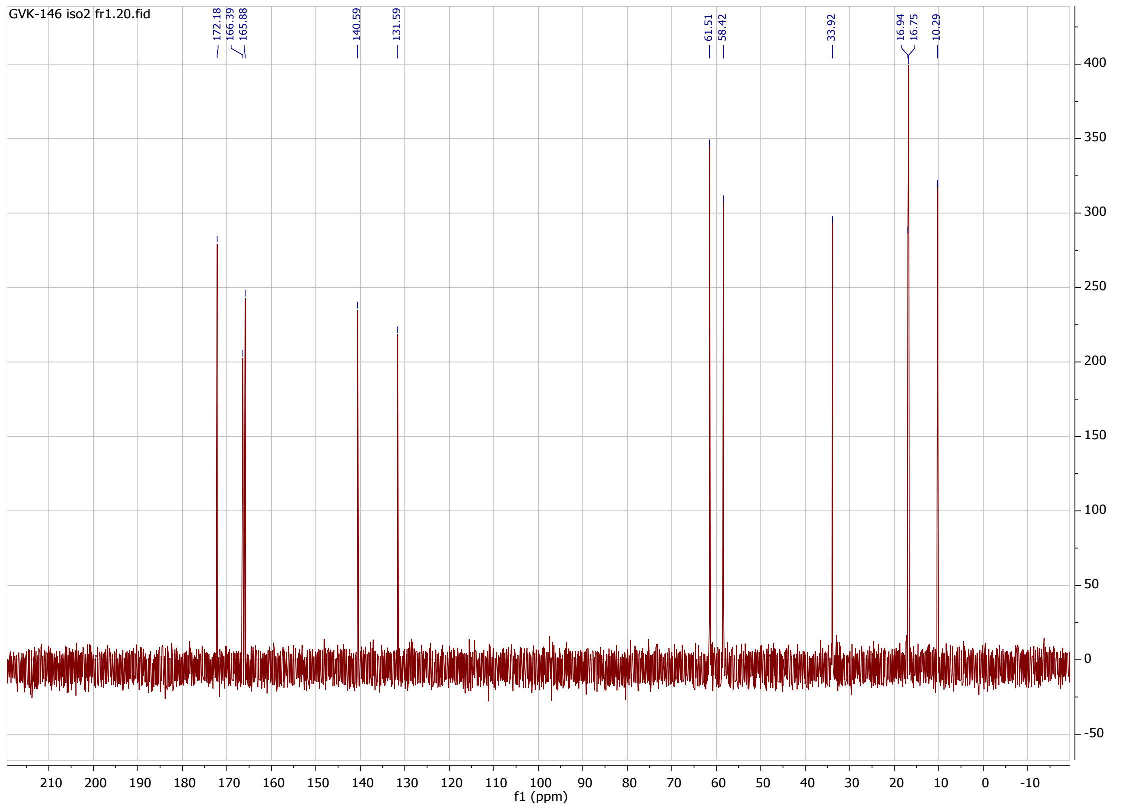

Chromatogram

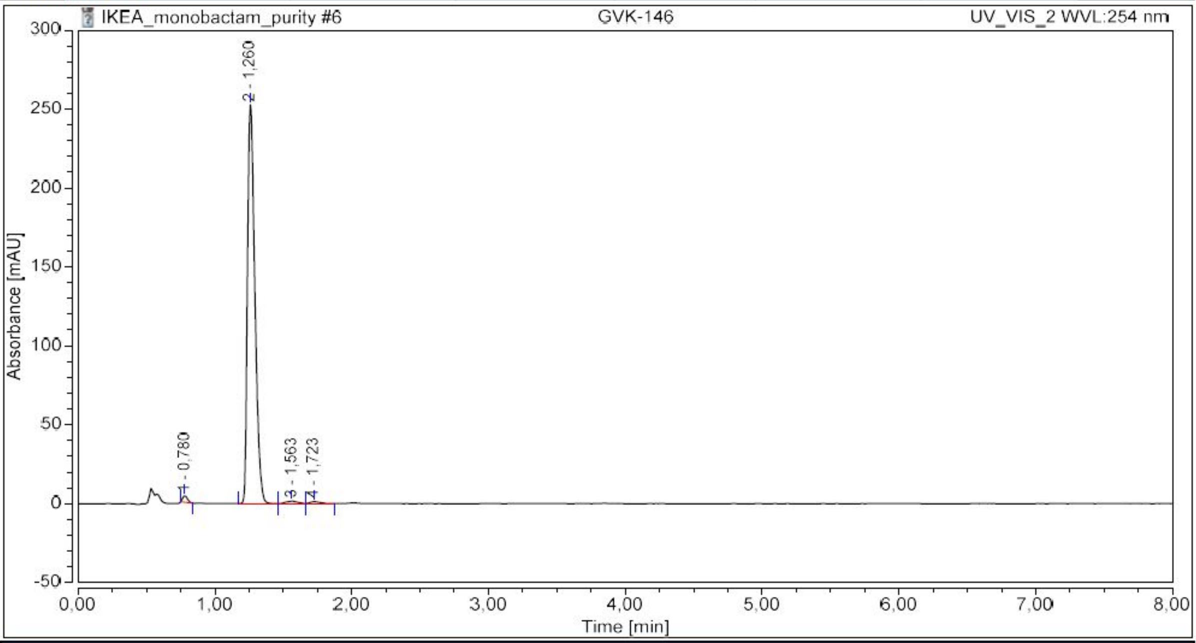

Integration Results

No.	Peak Name	Retention Time min	Area mAU*min	Height mAU	Relative Area %	Relative Height %	Amount n.a.
1		0,780	0,168	4,131	1,05	1,59	n.a.
2		1,260	15,654	252,772	97,49	97,37	n.a.
3		1,563	0,138	1,538	0,86	0,59	n.a.
4		1,723	0,097	1,150	0,60	0,44	n.a.
**Total:**			**16,058**	**259,591**	**100,00**	**100,00**	

HRMS: (ESI–), calc. for $C_{11}H_{14}O_5N_3S_2$ $[M–H]^-$ 332.03804, found 332.03760.

(*Z*)-3-(2-(5-amino-1,2,4-thiadiazol-3-yl)-2-(ethoxyimino)acetamido)-2-methyl-4-oxoazetidine-1-sulfonic acid, DIPEA salt (**24**)

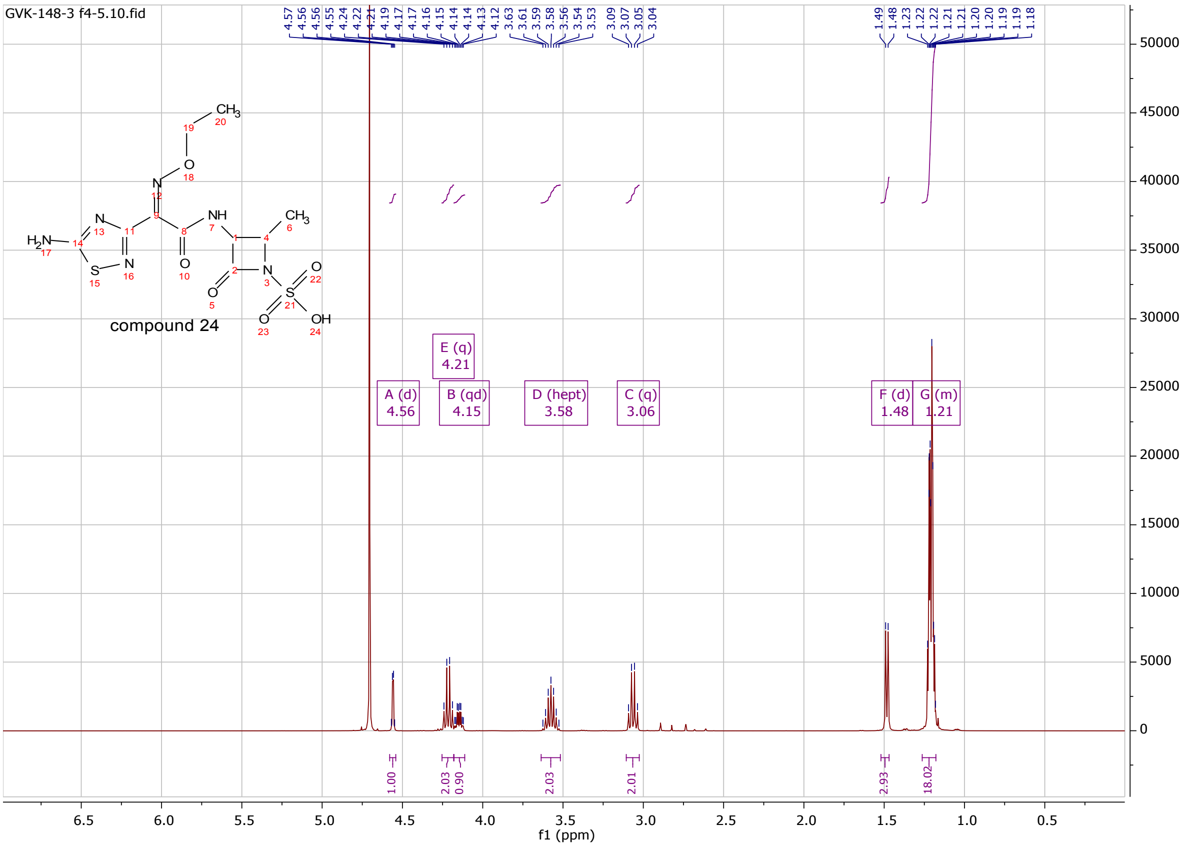

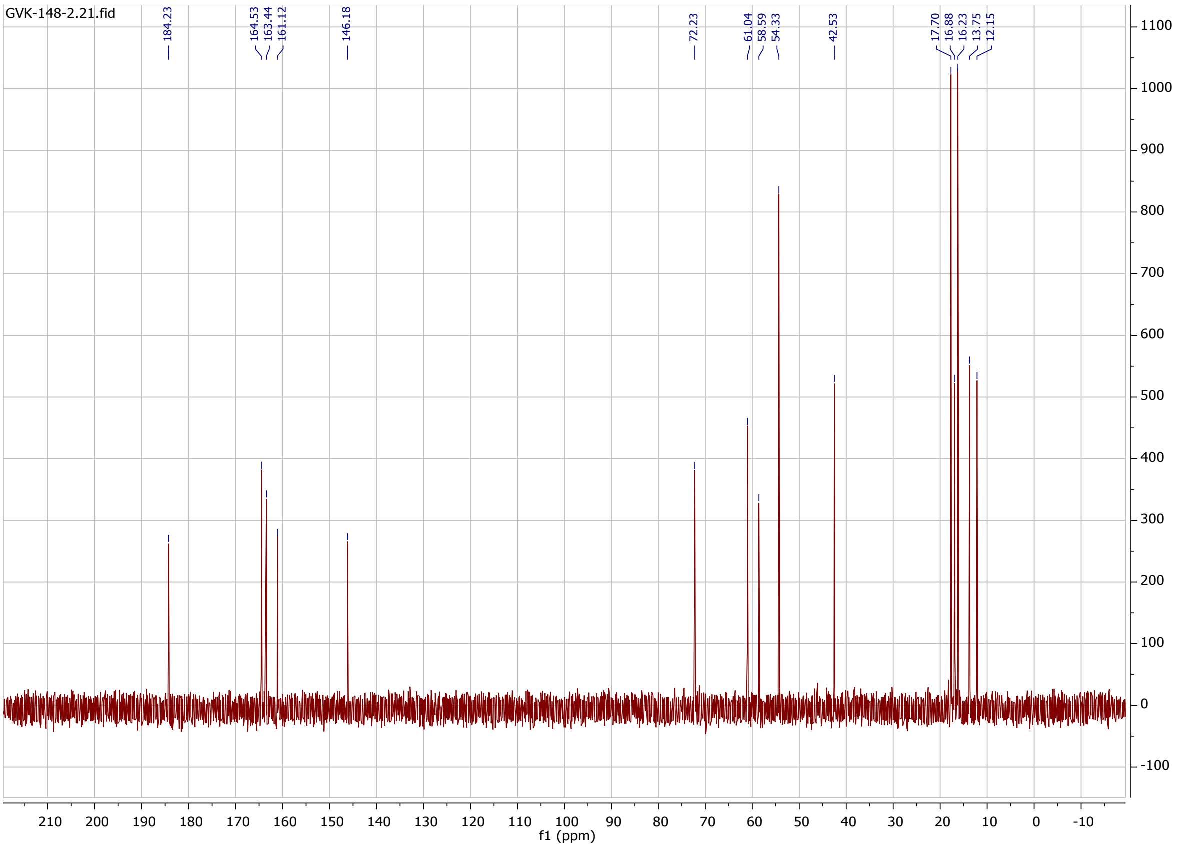

Chromatogram

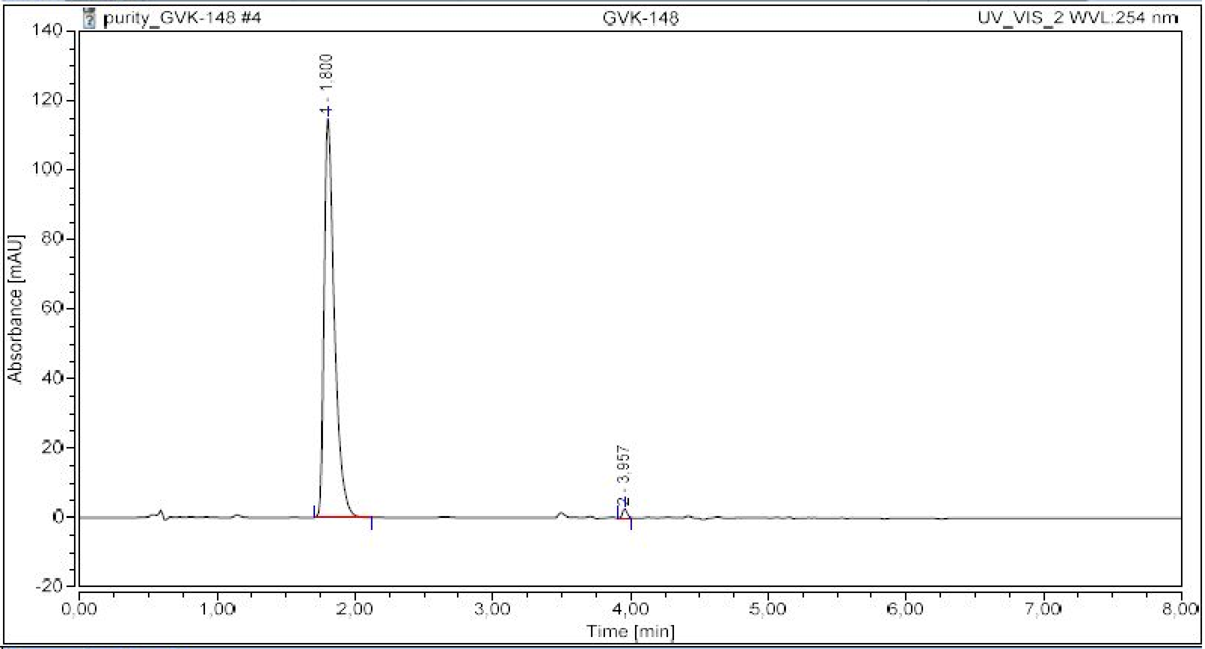

Integration Results

No.	Peak Name	Retention Time<br>min	Area<br>mAU*min	Height<br>mAU	Relative Area<br>%	Relative Height<br>%	Amount<br>n.a.
1		1,800	9,904	115,007	99,03	97,74	n.a.
2		3,957	0,097	2,660	0,97	2,26	n.a.
**Total:**			**10,001**	**117,667**	**100,00**	**100,00**	

HRMS: (ESI–), calc. for $C_{10}H_{13}O_6N_6S_2$ $[M–H]^-$ 377.03325, found 377.03488.

Potassium (2*S*,3*S*)-2-methyl-3-(2-(2-methylthiazol-4-yl)acetamido)-4-oxoazetidine-1-sulfonate (**25**)

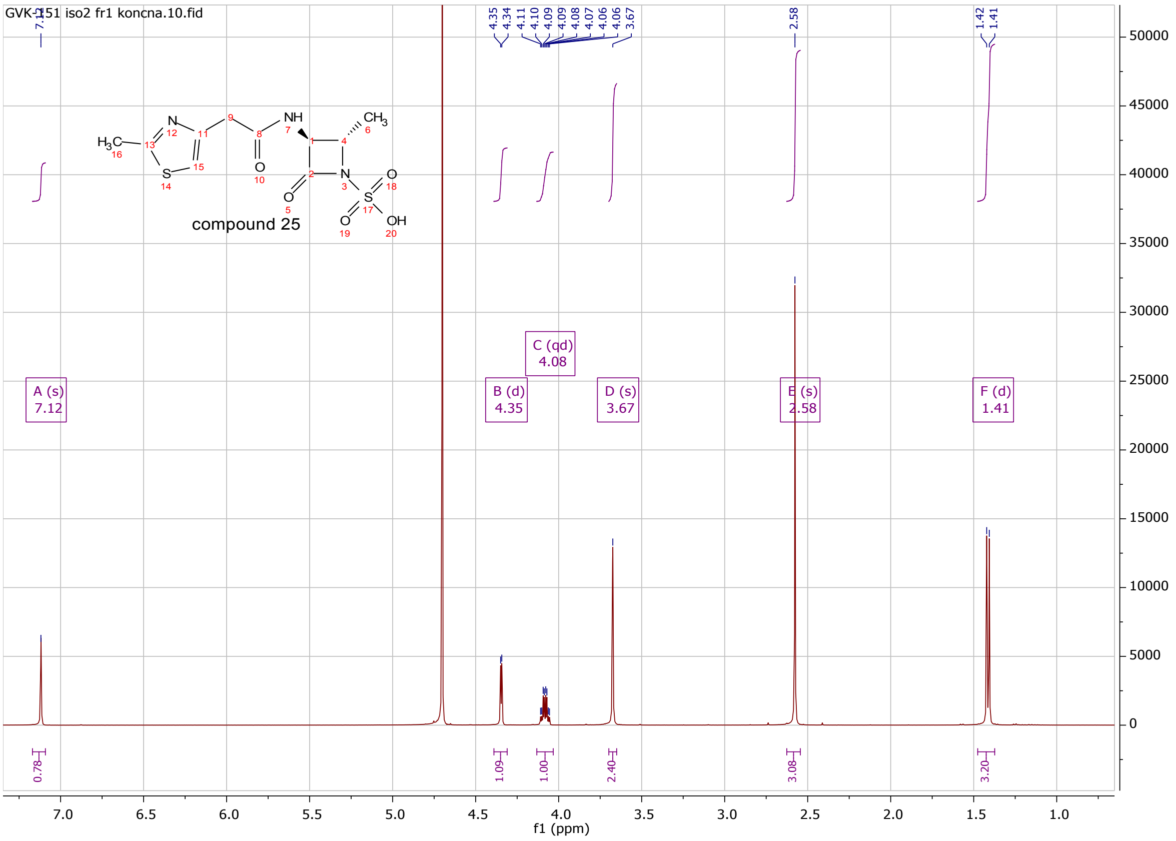

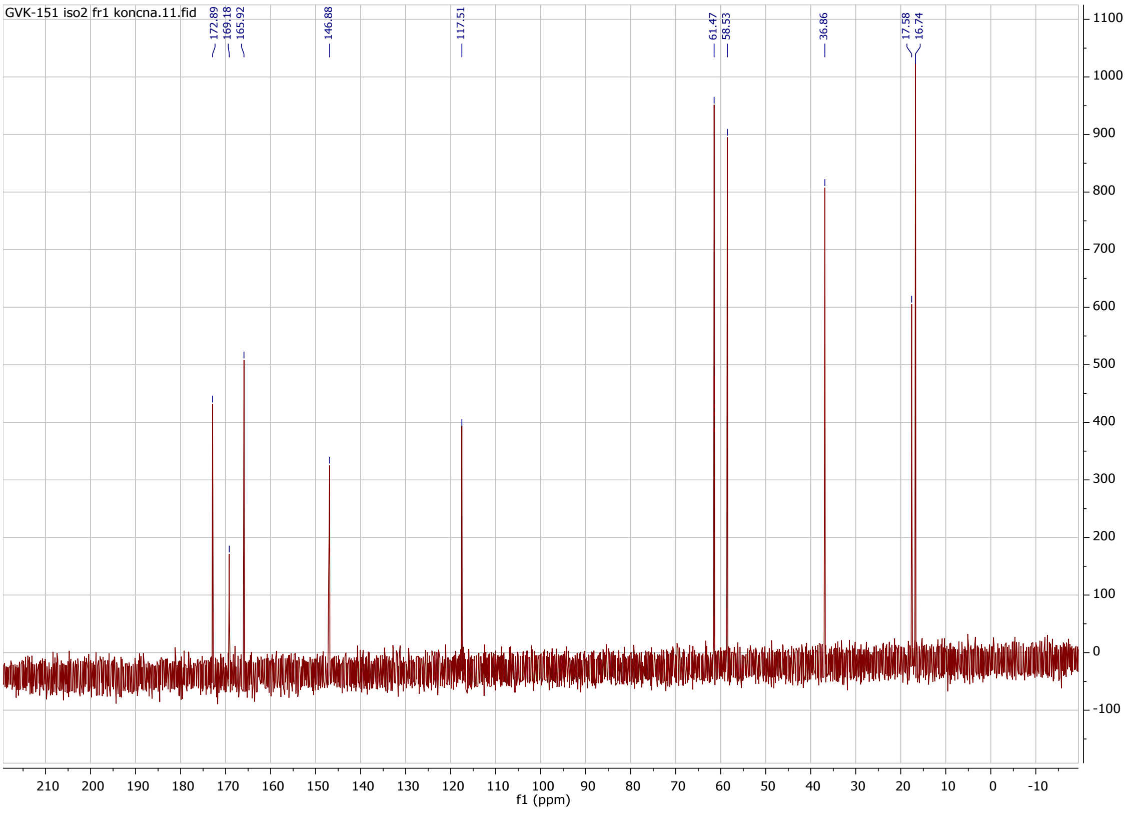

HRMS: (ESI–), calc. for $C_{10}H_{12}O_5N_3S_2$ $[M–H]^-$ 318.02239, found 318.02190.

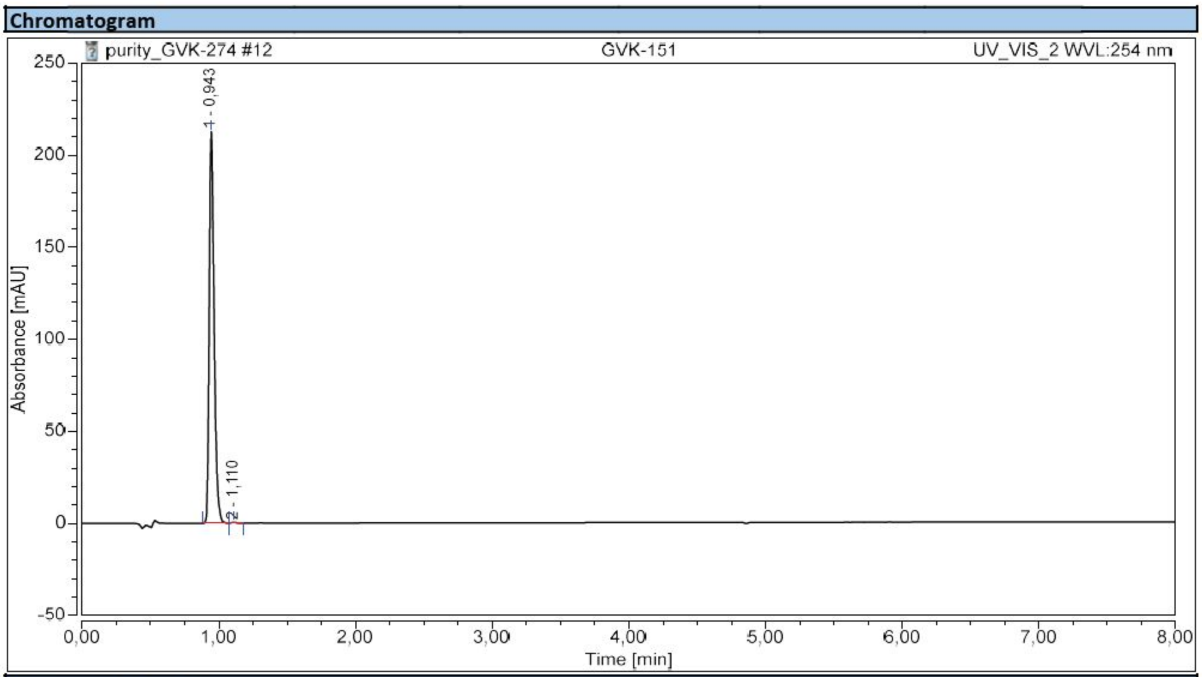

Integration Results

No.	Peak Name	Retention Time min	Area mAU*min	Height mAU	Relative Area %	Relative Height %	Amount n.a.
1		0,943	8,823	212,828	99,73	99,76	n.a.
2		1,110	0,024	0,516	0,27	0,24	n.a.
**Total:**			**8,847**	**213,344**	**100,00**	**100,00**	

Potassium (2*S*,3*S*)-3-((*Z*)-2-(2-aminothiazol-4-yl)-2-((2-methoxy-2-oxoethoxy)imino)acetamido)-2-methyl-4-oxoazetidine-1-sulfonate (**26**)

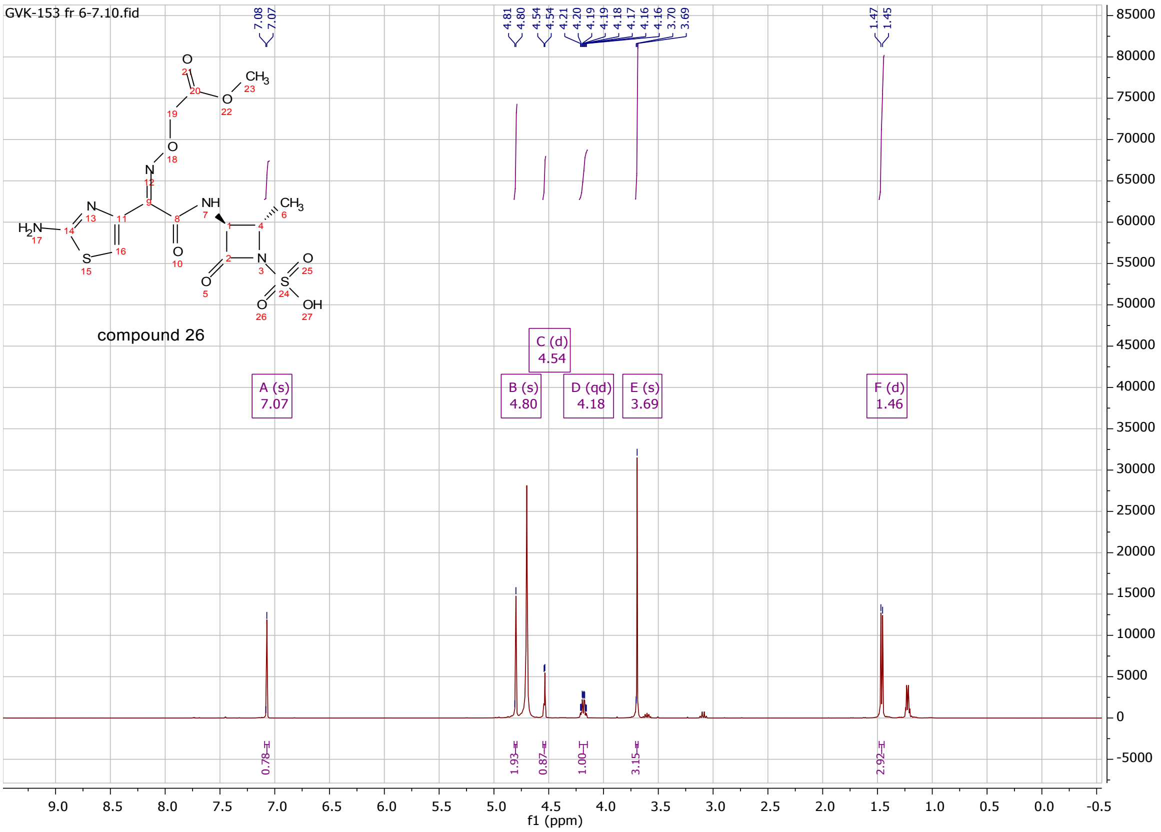

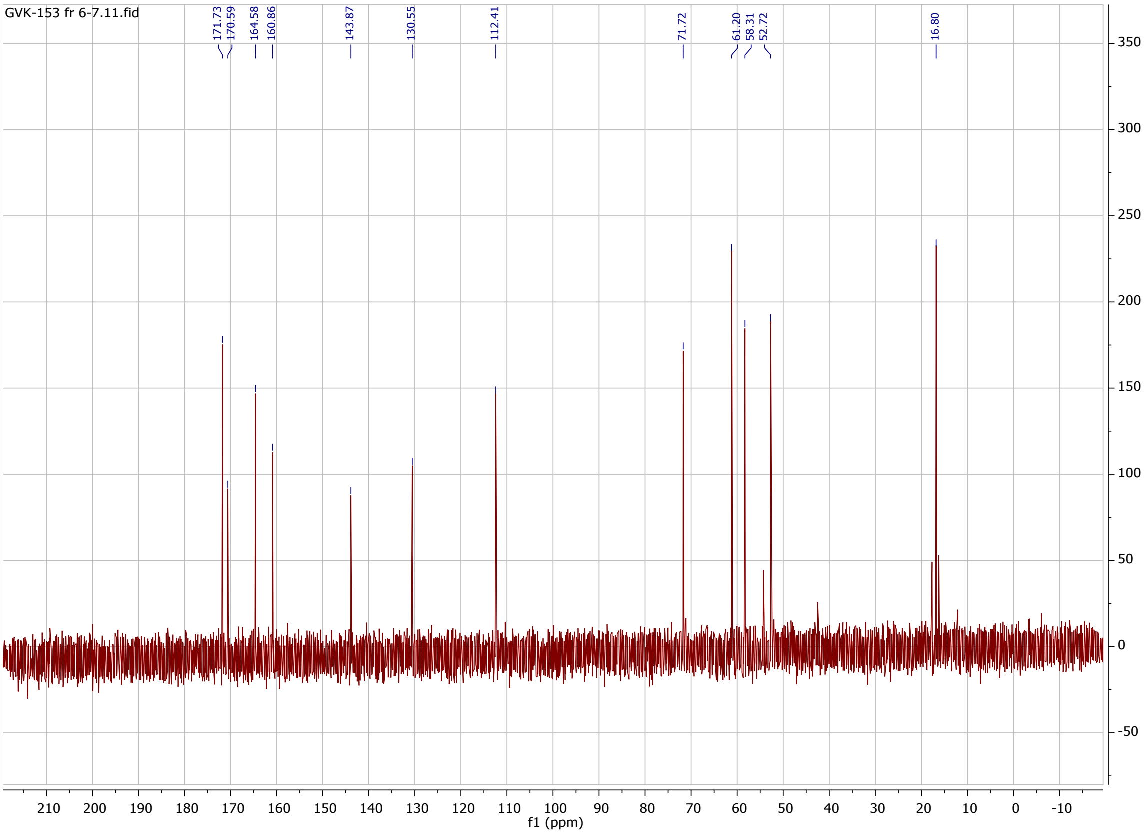

Chromatogram

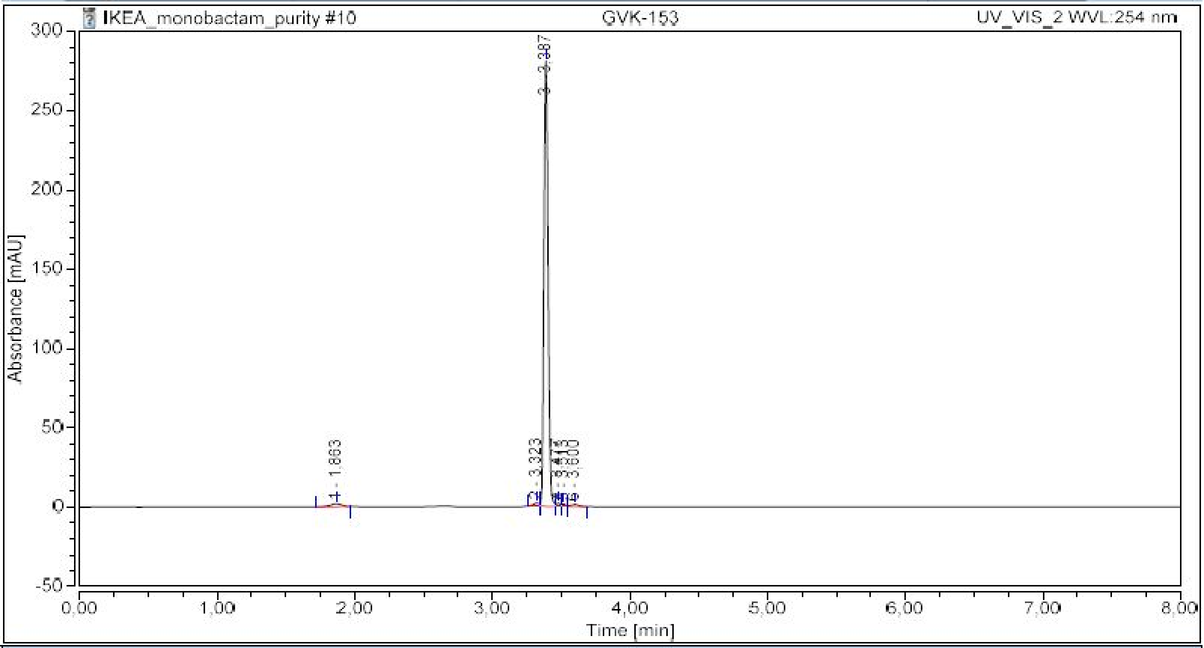

Integration Results

No.	Peak Name	Retention Time min	Area mAU*min	Height mAU	Relative Area %	Relative Height %	Amount n.a.
1		1,863	0,179	1,945	1,73	0,67	n.a.
2		3,323	0,097	2,196	0,94	0,76	n.a.
3		3,387	9,892	280,904	95,32	96,81	n.a.
4		3,477	0,066	2,131	0,63	0,73	n.a.
5		3,513	0,056	1,583	0,54	0,55	n.a.
6		3,600	0,087	1,407	0,84	0,48	n.a.
**Total:**			**10,378**	**290,166**	**100,00**	**100,00**	

HRMS: (ESI–), calc. for $C_{12}H_{14}O_8N_5S_2$ $[M–H]^-$ 420.02893, found 420.02856.

(2*S*,3*S*)-3-((*Z*)-2-(2-aminothiazol-4-yl)pent-2-enamido)-2-methyl-4-oxoazetidine-1-sulfonic acid, DIPEA salt (**27**)

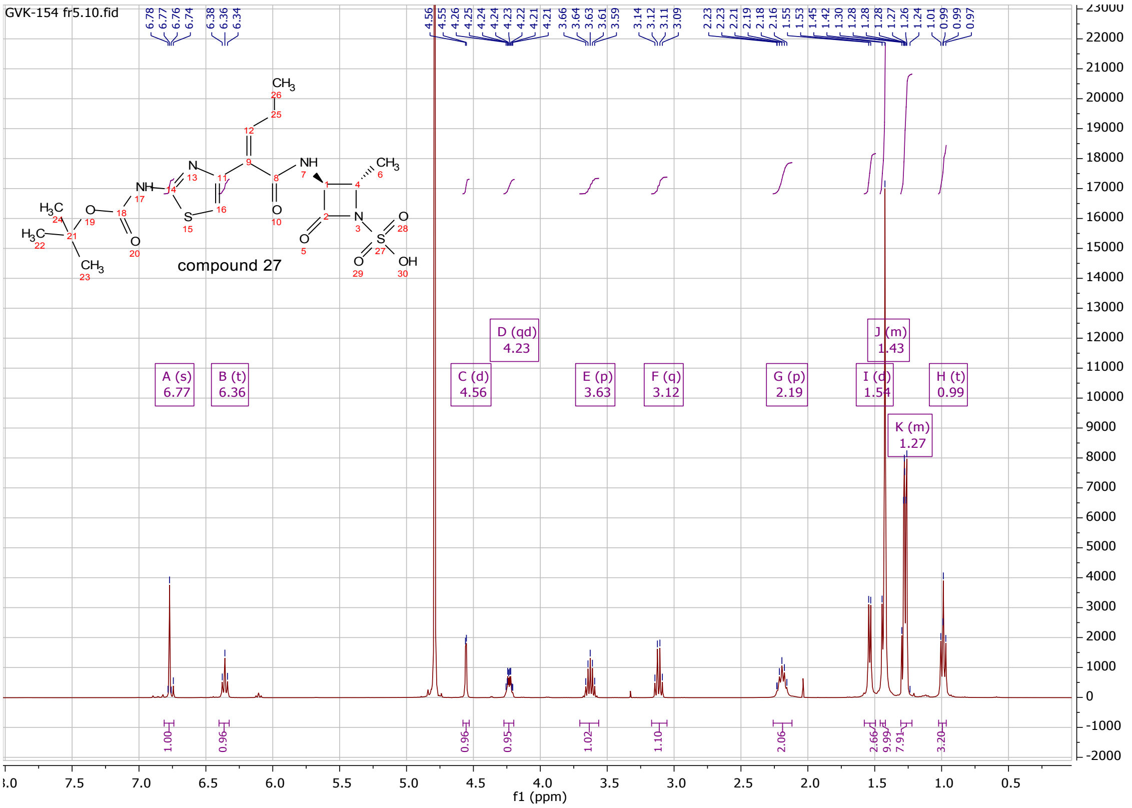

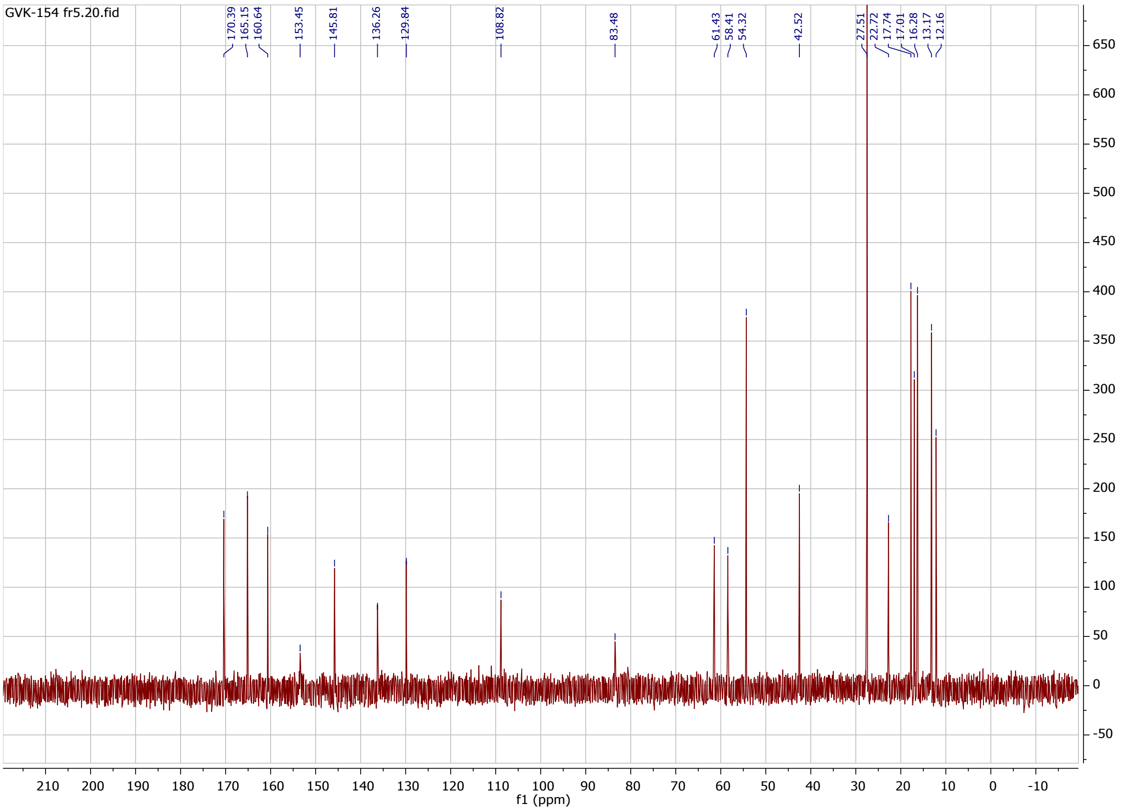

Chromatogram

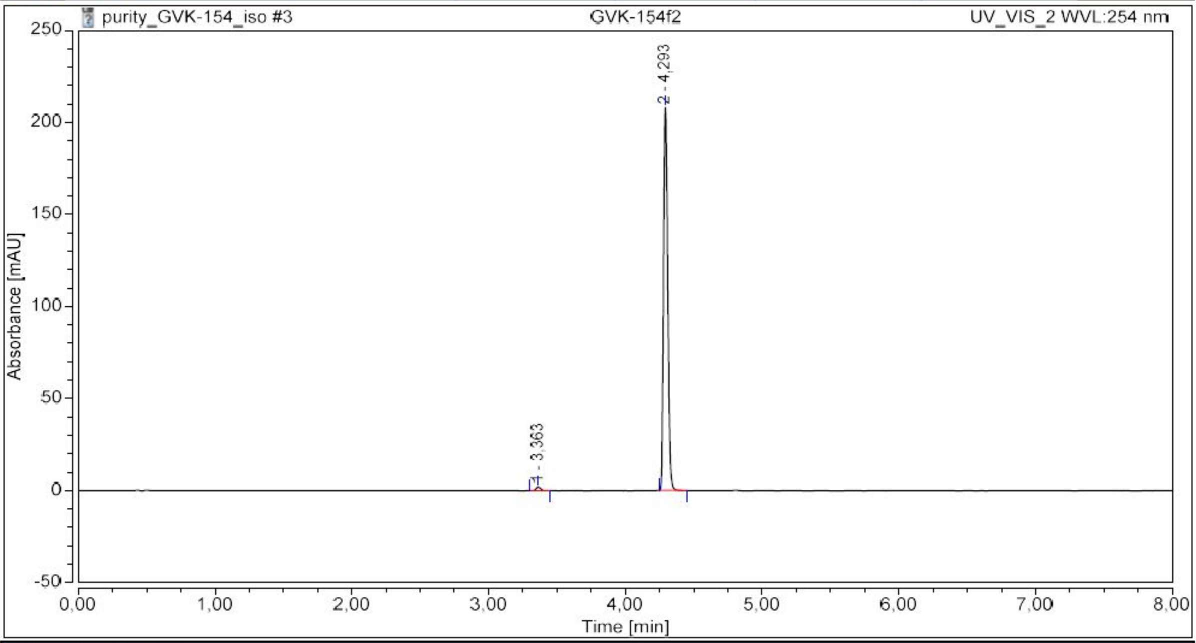

Integration Results

No.	Peak Name	Retention Time min	Area mAU*min	Height mAU	Relative Area %	Relative Height %	Amount n.a.
1		3,363	0,070	1,972	0,95	0,94	n.a.
2		4,293	7,336	208,063	99,05	99,06	n.a.
**Total:**			**7,406**	**210,034**	**100,00**	**100,00**	

HRMS: (ESI–), calc. for $C_{17}H_{23}O_7N_4S_2$ $[M–H]^-$ 459.10137, found 459.10172.

INTERACTIONS OF SYNTHETIZED COMPOUNDS

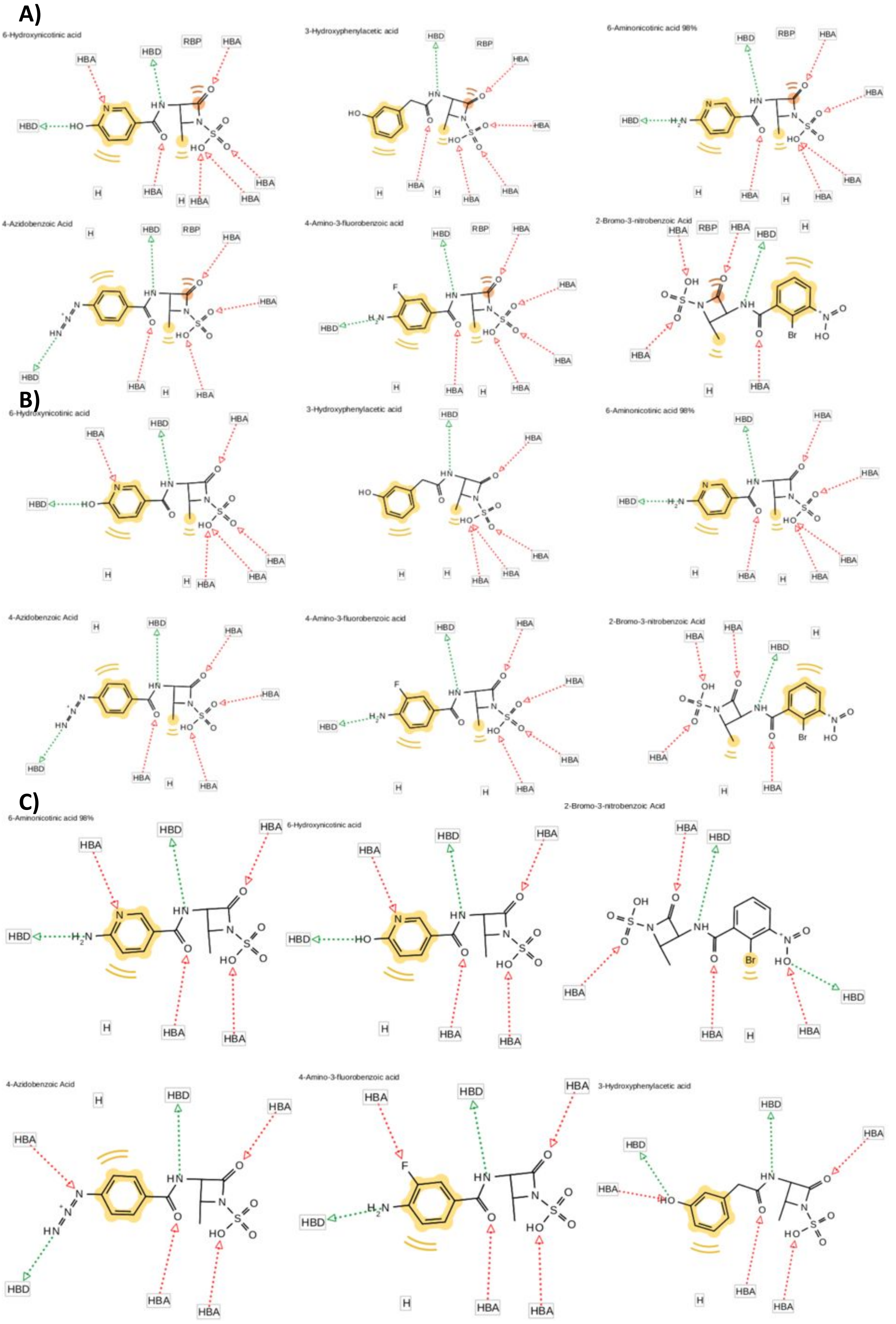

Figure S1. Interactions of synthetized compounds based on three distinct pharmacophore models A–C.

Table S1: Structures and scores for the compounds fitting all three models.

Name	Smiles	Score (bond)	Mol. Index	Source Database	Score (no_bond)	Score (no_bond _val)	CombinedScore	Structure
3-Bromo-6-fluoro-1H-indazole-4-carboxylic acid	Brc1[nH]nc2c1c(cc(F)c2)C(=O)N[C@H]3[C@H](N(S(=O)(=O)O)C3=O)C	115,9549	494	BL_screening_database.ldb	115,3883	75,80937	307,15	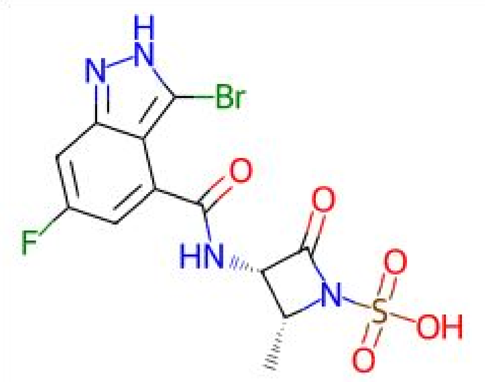
6-Aminonicotinic acid 98%	S(=O)(=O)(O)N2[C@H]([C@H](NC(=O)c1cnc(N)cc1)C2=O)C	116,9597	394	BL_screening_database.ldb	106,906	75,65255	299,52	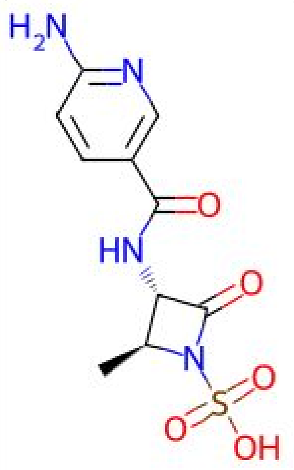

6-Hydroxynicotinic acid	S(=O)(=O)(O)N2[C@H]([C@H](NC(=O)c1cnc(O)cc1)C2=O)C	116,9104	8	BL_screening_database.ldb	106,8535	75,70515	299,47	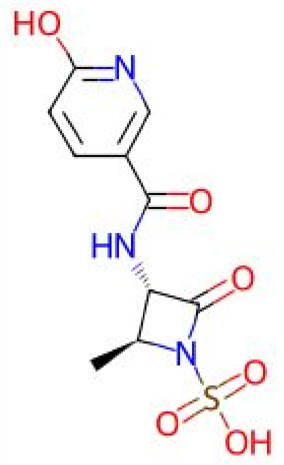
2-amino-?-(methoxyimino)-4-thiazoleacetic acid	s1c(nc(c1)C(=NOC)C(=O)N[C@H]2[C@@H](N(S(=O)(=O)O)C2=O)C)N	116,4396	297	BL_screening_database.ldb	106,4565	76,70313	299,6	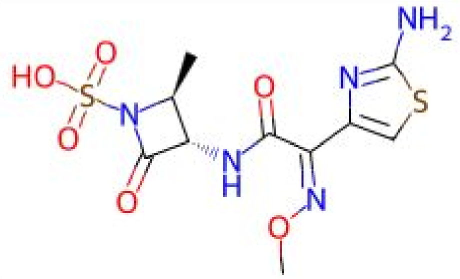

4-Amino-3-fluorobenzoic acid	S(=O)(=O)(O)N2[C@H]([C@H](NC(=O)c1cc(F)c(N)cc1)C2=O)C	116,2311	487	BL_screening_database.ldb	106,1759	75,62978	298,04	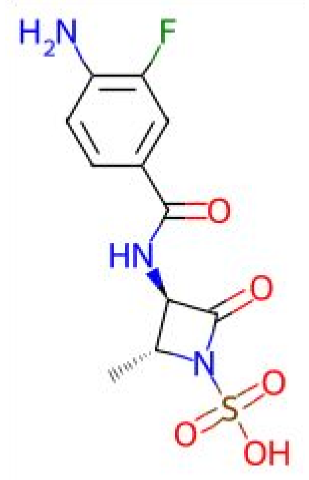
3,4-Dihydroxybenzoic acid, 97%	S(=O)(=O)(O)N2[C@H]([C@H](NC(=O)c1cc(O)c(O)cc1)C2=O)C	115,8604	174	BL_screening_database.ldb	105,921	75,35054	297,13	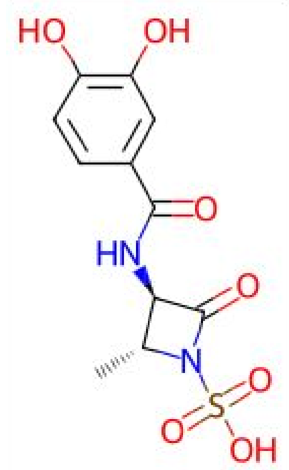

3-hydroxy-2-methylbenzoic acid	S(=O)(=O)(O)N2[C@@H]([C@H](NC(=O)c1c(c(O)ccc1)C)C2=O)C	115,9737	500	BL_screening_database.ldb	105,8904	75,85316	297,71	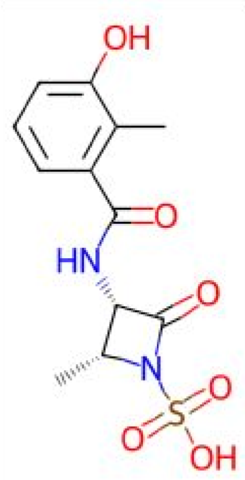
2-Chloro-3,4-dihydroxybenzoic acid	Clc1c(O)c(O)ccc1C(=O)N[C@H]2[C@@H](N(S(=O)(=O)O)C2=O)C	116,4866	490	BL_screening_database.ldb	105,7605	75,69177	297,94	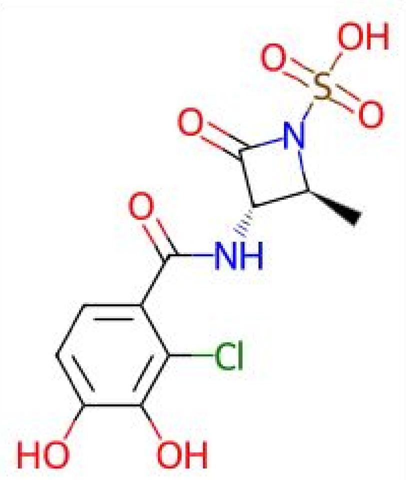

2-Amino-thiazole-4-carboxylic acid	s1c(nc(c1)C(=O)N[C@H]2[C@@H](N(S(=O)(=O)O)C2=O)C)N	115,7788	515	BL_screening_database.ldb	105,7311	75,33313	296,84	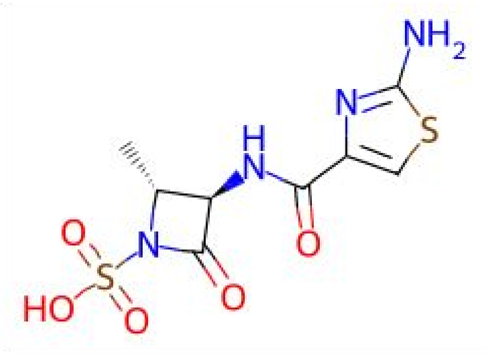
2,3-Dihydroxybenzoic acid, 99%	S(=O)(=O)(O)N2[C@H]([C@H](NC(=O)c1c(O)c(O)ccc1)C2=O)C	115,8048	176	BL_screening_database.ldb	105,608	75,70724	297,12	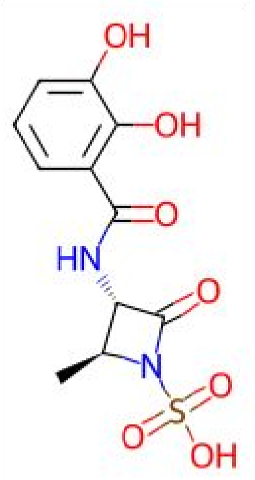

3-Hydroxybenzoic acid	S(=O)(=O)(O)N2[C@H]([C@H](NC(=O)c1cc(O)ccc1)C2=O)C	115,7245	6	BL_screening_database.ldb	105,5688	75,51288	296,8	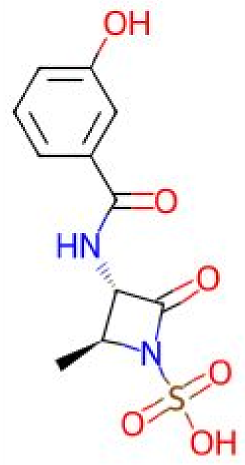
2-Bromo-3-nitrobenzoic Acid	Brc1c([N+](=O)O)cccc1C(=O)N[C@H]2[C@@H](N(S(=O)(=O)O)C2=O)C	115,985	533	BL_screening_database.ldb	105,3924	75,61292	296,98	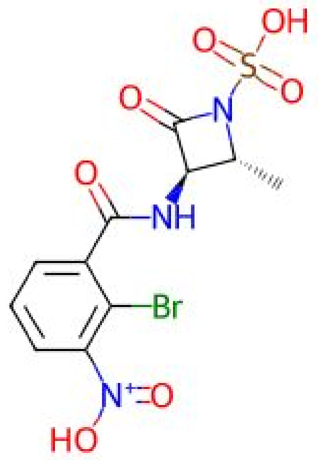

3-Carboxybenzene boronic acid	S(=O)(=O)(O)N2[C@H]([C@H](NC(=O)c1cc(ccc1)B(O)O)C2=O)C	115,5333	395	BL_screening_database.ldb	105,3604	75,81979	296,71	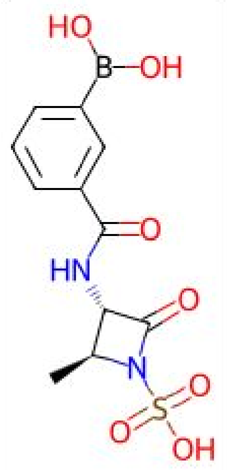
4-Hydroxybenzoic acid	S(=O)(=O)(O)N2[C@H]([C@H](NC(=O)c1ccc(O)cc1)C2=O)C	106,7746	4	BL_screening_database.ldb	96,66576	75,23294	278,67	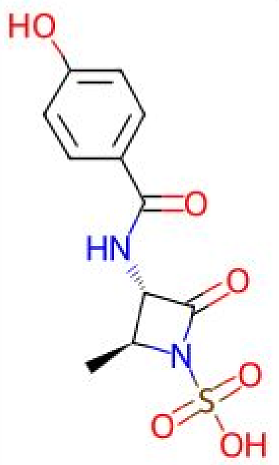

2,4-dihydroxybenzoic acid	S(=O)(=O)(O)N2[C@H]([C@H](NC(=O)c1c(O)cc(O)cc1)C2=O)C	106,7773	336	BL_screening_database.ldb	96,66399	75,29996	278,74	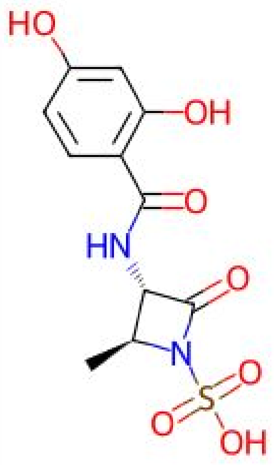
4-hydroxy-3-methoxybenzoic acid	S(=O)(=O)(O)N2[C@H]([C@H](NC(=O)c1cc(OC)c(O)cc1)C2=O)C	106,5882	318	BL_screening_database.ldb	96,4759	74,90981	277,98	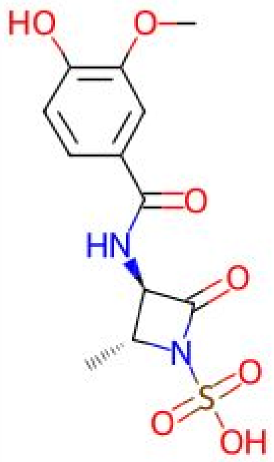

3-ACETYL-4-HYDROXY-BENZOIC ACID	S(=O)(=O)(O)N2[C@H]([C@H](NC(=O)c1cc(c(O)cc1)C(=O)C)C2=O)C	106,5253	496	BL_screening_database.ldb	96,42832	75,18633	278,15	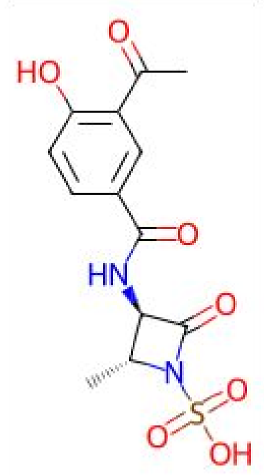
3-chloro-4-hydroxybenzoic acid hemihydrate	Clc1c(O)ccc(c1)C(=O)N[C@H]2[C@@H](N(S(=O)(=O)O)C2=O)C	106,5067	316	BL_screening_database.ldb	96,38745	75,34717	278,25	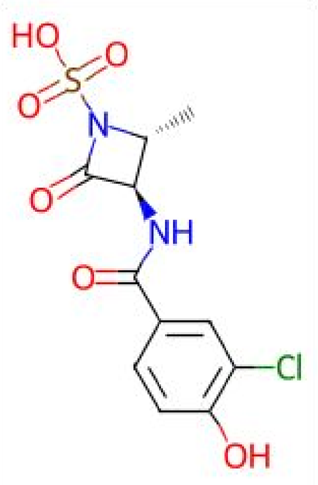

3-Bromo-4-hydroxybenzoic Acid	Brc1c(O)ccc(c1)C(=O)N[C@H]2[C@@H](N(S(=O)(=O)O)C2=O)C	106,4737	662	BL_screening_database.ldb	96,32895	75,21504	278,02	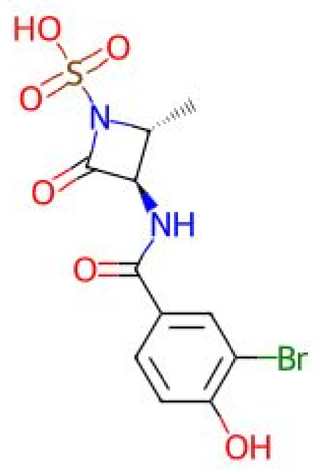
4-hydroxy-3-nitrobenzoic acid	S(=O)(=O)(O)N2[C@H]([C@H](NC(=O)c1cc([N+](=O)O)c(O)cc1)C2=O)C	106,4374	13	BL_screening_database.ldb	96,31241	75,21501	277,97	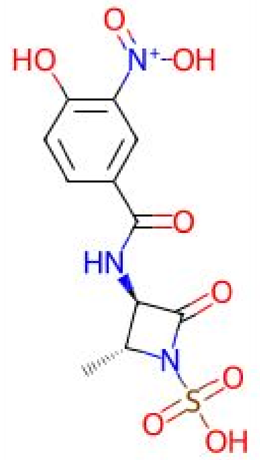

4-Amino-3-mercaptobenzoic acid, 97%	S(=O)(=O)(O)N2[C@H]([C@H](NC(=O)c1cc(S)c(N)cc1)C2=O)C	115,5829	633	BL_screening_database.ldb	96,27585	75,23895	287,1	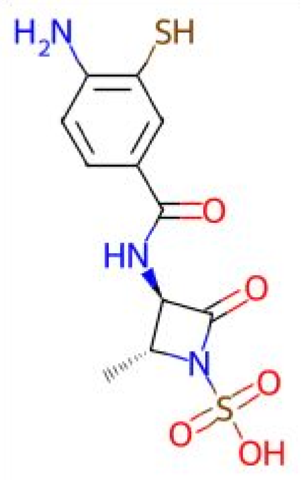
4-Azidobenzoic Acid	S(=O)(=O)(O)N2[C@H]([C@H](NC(=O)c1ccc(N=[N+]=N)cc1)C2=O)C	106,3281	410	BL_screening_database.ldb	96,19705	75,64928	278,18	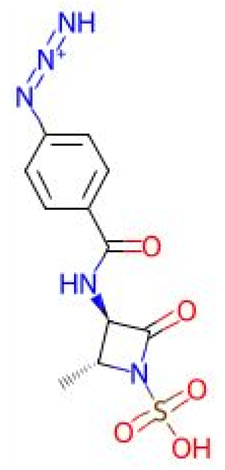

6-Aminopyridine-2-carboxylic acid	S(=O)(=O)(O)N2[C@H]([C@H](NC(=O)c1nc(N)ccc1)C2=O)C	105,7997	342	BL_screening_database.ldb	95,73414	75,02539	276,56	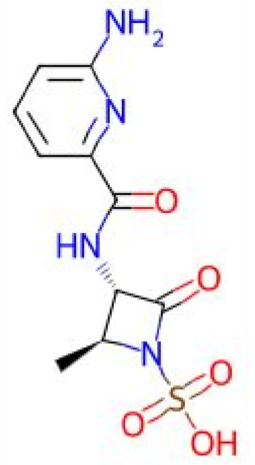
2-Amino-1,3-benzothiazole-6-carboxylic acid	s1c(nc2c1cc(cc2)C(=O)N[C@H]3[C@H](N(S(=O)(=O)O)C3=O)C)N	105,8476	451	BL_screening_database.ldb	95,6778	75,13715	276,67	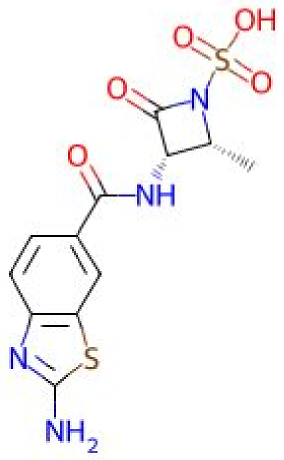

3-Hydroxyphenylacetic acid	S(=O)(=O)(O)N2[C@H]([C@H](NC(=O)Cc1cc(O)ccc1)C2=O)C	96,1676	16	BL_screening_database.ldb	86,31283	75,16903	257,65	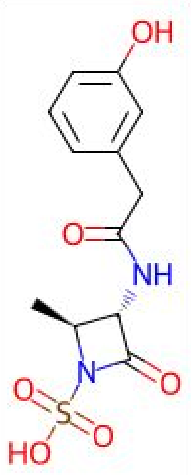
4-Bromo-3-nitrobenzoic acid	Brc1c([N+](=O)O)cc(cc1)C(=O)N[C@H]2[C@@H](N(S(=O)(=O)O)C2=O)C	105,6884	486	BL_screening_database.ldb	86,28152	75,74358	267,71	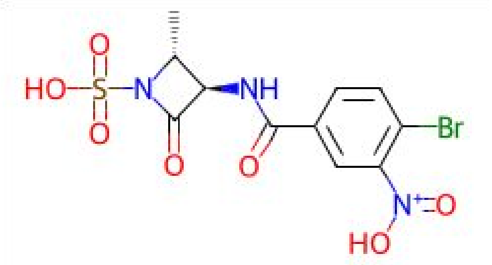

ENZYME INHIBITION CURVES AND MIC DETERMINATION AGAINST ESKAPE PATHOGENS

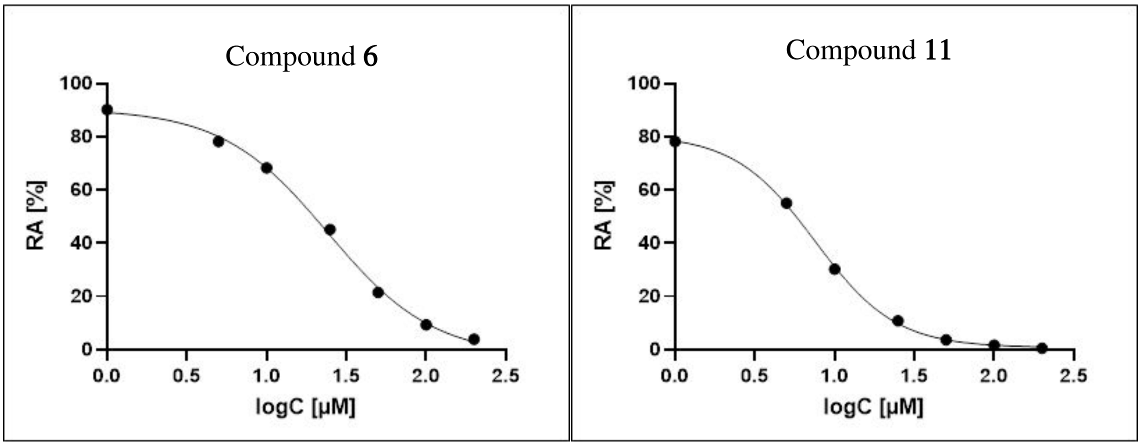

Figure S2: Determined IC_{50} curves for compounds **6** and **11** on PBP3 of *E. coli*.

Table S2. Minimum inhibitory concentration of first series monobactams against *S. aureus* ATCC 29213, *E. coli* ATCC 25922, *E. coli* N43 (CGSC no. 5583), *E. coli* D22 (CGSC no. 5163), *A. baumannii* 8C6 GES-14 (strain obtained from a European reference laboratory, EURL-AMR, DTU, Copenhagen, Denmark), *P. aeruginosa* RDK 184 (DSM 939; ATCC 15442), *K. pneumoniae* (RDK 070A; ATCC 51503), MRSA QA-11.7, *E. faecalis* ATCC 29212 and *E. faecium* (30088/46).

Cpd	MIC [µg/mL]									
	S. aureus	*E. coli*	ECD 22	EC N43	*A. baumannii*	*P. aeruginosa*	*K. pneumoniae*	MRSA	*E. faecalis*	*E. faecium*
**2**	>128	>128	>128	>128	>128	>128	>128	>128	>128	>128
**3**	>128	>128	>128	>128	>128	>128	>128	>128	>128	>128
**4**	>128	>128	>128	>128	>128	>128	>128	>128	>128	>128
**5**	>128	>128	>128	>128	>128	>128	>128	>128	>128	>128
**6**	64	128	128	128	>128	>128	>128	>128	>128	>128
**7**	>128	>128	>128	>128	>128	>128	>128	>128	>128	>128
**8**	>128	>128	64	128	128	>128	128	>128	>128	>128
**9a**	128	>128	>128	>128	>128	>128	>128	>128	>128	>128
**9b**	128	>128	>128	>128	>128	>128	>128	>128	>128	>128
**10a**	16	>128	>128	128	>128	>128	>128	>128	>128	>128
**10b**	64	>128	>128	>128	>128	>128	>128	>128	>128	>128
**11**	64	>128	>128	64	>128	>128	>128	>128	>128	>128
**12a**	128	>128	>128	128	>128	>128	>128	>128	>128	>128
**12b**	>128	>128	>128	>128	>128	>128	>128	>128	>128	>128

**13a**	64	>128	>128	128	>128	>128	>128	>128	>128	>128
**13b**	128	>128	>128	>128	>128	>128	>128	>128	>128	>128
**14a**	32	>128	>128	>128	>128	>128	>128	>128	>128	>128
**14b**	64	>128	>128	>128	>128	>128	>128	>128	>128	>128
**15a**	>128	>128	>128	>128	>128	>128	>128	>128	>128	>128
**15b**	>128	>128	>128	>128	>128	>128	>128	>128	>128	>128
**16a**	>128	>128	>128	>128	>128	>128	>128	>128	>128	>128
**16b**	>128	>128	>128	>128	>128	>128	>128	>128	>128	>128
**17**	128	32	32	16	>128	>128	>128	>128	>128	>128
**18**	>128	128	128	64	128	>128	>128	>128	>128	>128
**19**	>128	>128	>128	64	>128	>128	>128	>128	>128	>128
**21**	>128	128	64	64	>128	>128	>128	>128	>128	>128

Table S3. Minimum inhibitory concentration of second series monobactams MRSA QA-11.7, *E. faecalis* ATCC 29212 and *E. faecium* (30088/46).

**Compound**	**MIC [µg/mL]**		
	MRSA	*E. faecalis*	*E. faecium*
**22**	>128	>128	>128
**23**	>128	>128	>128
**24**	>128	>128	>128
**25**	>128	>128	>128
**26**	>128	>128	>128
**27**	N.D.	>128	>128
**28**	N.D.	>128	>128
aztreonam	N.D.	>128	>128

N.D. – not determined

Table S4. Minimum inhibitory concentration for compounds with different counterions calculated in µmol/L.

**Cpd**	**Counterion**	**MIC *S. aureus* (µM)**
**9a**	K+	340,9
**9b**	DIPEA	274,3
**10a**	K+	41,9
**10b**	DIPEA	135,4
**12a**	K+	361,2
**12b**	DIPEA	>287.3
**13a**	K+	172,6
**13b**	DIPEA	277,1
**14a**	K+	77,1
**14b**	DIPEA	126,4

**15a**	K+	>349,3
**15b**	DIPEA	>279,7
**16a**	K+	>322,8
**16b**	DIPEA	>262,5

STATISTICAL PARAMETERS OF CRYSTAL STRUCTURES

Table S5. Data collection, phasing and structure refinement statistics.

**DIFFRACTION DATA**	**6**	**9**	**10**	**11**	**15**
Wavelength (Å)	**0.965459**	**0.965459**	**0.965459**	**0.965459**	**0.965459**
Scan-range (°)	104	82	113	110	101
Oscillation (°)	0.05	0.05	0.05	0.05	0.05
Space group	$C222_1$	$C222_1$	$C222_1$	$C222_1$	$C222_1$
a (Å)	96.66	96.94	96.48	97.02	96.40
b (Å)	148.24	148.82	149.56	148.68	148.53
c (Å)	98.83	98.64	99.47	98.65	98.61
$\alpha=\beta=\gamma$ (°)	90	90	90	90	90
Mosaicity (°)	0.124	0.108	0.139	0.160	0.091
Overall resolution (Å)	44.00-1.88	41.11-1.57	44.30-1.74	44.14/1.53	44.05/1.81
Observed/unique reflections	218226/56578	294314/95014	299479/71443	420866/102722	235856/61904
High resolution shell (Å)	1.99-1.88	1.66-1.57	1.84-1.74	1.63-1.53	1.92-1.81
Completeness (%) (last shell)	97.6 (98.0)	95.6 (97.5)	96.7 (97.7)	95.8 (93.4)	95.8 (96.2)
R_{sym} (last shell)	6.6 (292.5)	4.0 (139.9)	5.8 (209.3)	5.1 (180.0)	5.2 (139.3)
$I/\sigma(I)$ (last shell)	8.39 (0.48)	11.51 (0.81)	9.88 (0.53)	10.50 (0.65)	11.31 (1.13)
$CC_{1/2}$	99.8 (9.1)	99.9 (24.5)	99.9 (20.6)	99.9 (24.4)	99.8 (49.1)
Wilson plot B-factor (Å^2)	56.70	39.01	48.21	39.27	49.02
**BUILDING**					
PBP1b Mol/ASU	1	1	1	1	1
Phaser LLG	8860	15474	10743	13177	12487
ARP/wARP residues	428	465	446	466	447
**REFINEMENT**					
Initial R_{work}/R_{free} (%)	21.21/26.50	18.48/20.20	19.90/23.18	19.70/22.21	19.28/22.14
Final R_{work}/R_{free} (%)	18.33/23.28	17.10/18.71	18.11/20.91	18.24/20.49	16.89/19.07
TLS R_{work}/R_{free} (%)	17.79/22.17	16.14/17.42	17.54/20.15	17.62/19.51	16.01/18.09
Mean B-factor (Å^2)	65.82	39.32	51.83	38.30	51.29
**STEREOCHEMICAL QUALITY**					
RMSD, bond lengths (Å)	0.007	0.008	0.008	0.008	0.009

RMSD, bond angles (°)	1.294	1.292	1.298	1.286	1.289
No. of protein residues	451	469	455	469	455
No. of atoms	3745	4207	3959	4231	3968
No. of protein atoms	3494	3662	3556	3665	3541
No. of monobactam atoms	21	46	46	22	44
No. of water molecules	200	465	329	511	356
No. of ions molecules	27	27	29	33	27
Residues in most favoured/allowed region of Ramachandran plot (%)	99.2	99.3	99.2	99.2	99.0
**DIFFRACTION DATA**	**21**	**22**	**24**	**26**	**Aztreaonam**
Wavelength (Å)	0.965459	0.965459	0.965459	0.965459	0.965459
Scan-range (°)	100	104	104	89	130
Oscillation (°)	0.05	0.05	0.05	0.05	0.05
Space group	$C222_1$	$C222_1$	$C222_1$	$C222_1$	$C222_1$
a (Å)	97.16	97.47	97.62	97.24	97.31
b (Å)	149.08	148.15	147.67	148.30	147.18
c (Å)	98.46	97.96	97.90	98.14	98.12
$\alpha=\beta=\gamma$ (°)	90	90	90	90	90
Mosaicity (°)	0.074	0.049	0.079	0.076	0.094
Overall resolution (Å)	48.59-1.50	48.74-1.52	43.96-1.51	43.57-1.57	41.91-1.65
No. observed/unique reflections	413886/110122	389348/101655	414676/106199	318856/94733	388180/82070
High resolution shell (Å)	1.59-1.50	1.61-1.52	1.61-1.51	1.66-1.57	1.76-1.65
Completeness (%) (last shell)	96.6 (98.5)	93.5 (81.6)	96.0 (93.3)	95.9 (97.0)	98.7 (95.5)
R_{sym} (last shell)	5.2 (156.4)	3.9 (188.1)	4.0 (291.7)	5.3 (172.5)	9.2 (334.7)
$I/\sigma(I)$ (last shell)	10.26 (0.75)	13.28 (0.57)	12.38 (0.42)	10.29 (0.73)	8.03 (0.55)
$CC_{1/2}$	99.8 (27.2)	99.9 (20.2)	99.9 (13.7)	99.8 (22.7)	99.6 (20.2)
Wilson plot B-factor (Å^2)	35.46	40.41	41.71	42.78	41.43
**MOLECULAR REPLACEMENT**					
PBP1b Mol/ASU	1	1	1	1	1
Phaser LLG	14394	14784	13641	15299	12648
ARP/wARP (residues)	462	459	443	462	448
**REFINEMENT**					
Initial R_{work}/R_{free} (%)	18.44/21.89	21.19/23.62	21.53/24.72	20.14/23.47	20.72/22.24

Final R_{work}/R_{free} (%)	17.43/20.78	19.25/21.86	19.31/22.25	18.71/22.25	18.86/20.93
TLS R_{work}/R_{free} (%)	16.69/19.55	18.30/20.10	18.33/21.08	17.67/20.52	17.35/18.98
Mean B-factor (Å^2)	35.04	43.16	46.63	44.83	46.27
**STEREOCHEMICAL QUALITY**					
RMSD, bond lengths (Å)	0.009	0.008	0.008	0.009	0.008
RMSD, bond angles (°)	1.299	1.294	1.294	1.294	1.294
No. of protein residues	470	470	463	470	448
No. of atoms	4139	4197	4093	4097	3962
No. of protein atoms	3660	3658	3579	3656	3496
No. of monobactam atoms	40	30	24	27	28
No. of water molecules	406	477	459	377	404
No. of ions molecules	33	32	31	38	34
Residues in most favoured/allowed region of Ramachandran plot (%)	99.3	99.3	99.0	99.3	99.2
